# Cross-talk of m^6^A methylation modification and the tumor microenvironment composition in esophageal cancer

**DOI:** 10.3389/fimmu.2025.1572810

**Published:** 2025-07-07

**Authors:** Pan Song, Jinmao Ye, Haiyang Zhang, Yishu Li, Ruizhi Cao, Yang Feng, Lei Zhang, Min Sun

**Affiliations:** ^1^ Department of General Surgery, Taihe Hospital, Hubei University of Medicine, Shiyan, China; ^2^ Department of Radiation Oncology, Shanghai Ninth People’s Hospital, Shanghai Jiaotong University School of Medicine, Shanghai, China; ^3^ Department of Head, Neck and Pediatric Oncology, Zhongnan Hospital of Wuhan University, Wuhan, China; ^4^ Hubei Key Laboratory of Embryonic Stem Cell Research, Taihe Hospital, Hubei University of Medicine, Shiyan, China

**Keywords:** esophagus cancer, m^6^A, tumor microenvironment, immunotherapy, prognosis, immune infiltration

## Abstract

**Background:**

Esophageal cancer (EC) remains a significant clinical challenge, characterized by its aggressive nature and poor prognosis. Current therapeutic strategies, including targeted therapies, have limitations due to the complex interplay between tumor heterogeneity and the tumor microenvironment (TME). However, the specific contributions of N6-methyladenosine (m^6^A) methylation to the TME in EC are yet to be fully elucidated.

**Methods:**

Through comprehensive bioinformatics analyses, a detailed examination of m^6^A regulators were conducted in EC using datasets from The Cancer Genome Atlas (TCGA) and Gene Expression Omnibus (GEO). Single-cell RNA sequencing (scRNA-seq) and a consensus clustering algorithm was employed to classify m^6^A modification patterns and analyze their relationships with immune cell infiltration and clinical outcomes. Additionally, an m^6^A scoring system was developed based on principal component analysis to assess the prognostic value of identified m^6^A modification patterns.

**Results:**

The findings revealed two distinct m^6^A modification clusters associated with divergent TME characteristics and immune infiltration profiles. Patients exhibiting the immune-inflamed phenotype (m^6^A cluster B) demonstrated significantly improved survival compared to those with the immune-excluded phenotype (m^6^A cluster A). Notably, m^6^A scores correlated positively with immune cell presence and related with adverse prognostic outcomes, indicating their potential as predictive biomarkers for immunotherapy responses. A low m^6^A score indicated a better response to immunotherapy.

**Conclusion:**

This study highlights the critical role of m^6^A methylation in shaping the TME and influencing immune dynamics in EC. The m^6^A score developed herein provides a novel quantitative tool for predicting tumor behavior and treatment efficacy, paving the way for more personalized immunotherapeutic strategies in clinical practice. This scoring system illustrates a strong correlation of EC with TME immune cell composition, suggesting potential as a biomarker for targeted therapeutic strategies for EC.

## 1 Introduction

N6-methyladenosine (m^6^A) has been recognized as a crucial RNA modification. m^6^A modifications are dynamically regulated by various regulators, including methyltransferase complex writers such as METTL3, METTL14, METTL16, RBM15, RBM15B, VIRMA, WTAP and ZC3H13, and several binding proteins, such as FMR1, HNRNPC, HNRNPA2B1, IGFBP1/2/3, LRPPRC, RBMX, YTHDC1/2, and YTHDF1/2/3, have been identified as readers, as well as demethylases erasers such as FTO and ALKBH5. Numerous studies have demonstrated that aberrant expression of m^6^A core modification and reading proteins is implicated in diverse physiological and pathological processes, including biological growth and development, DNA damage repair, biological rhythms, angiogenesis, and various types of tumors (1). In recent years, substantial progress has been achieved in m^6^A epitranscriptomics, underscoring its pivotal roles in cancer initiation and progression by modulating RNA stability, mRNA splicing, microRNA processing, and mRNA translation (2). Unlike genetic events, m^6^A modifications are reversible, making epigenetic regulation particularly interesting for the development of new therapeutic technologies for cancer treatment.

Esophageal cancer has the sixth highest cancer-related mortality rate, but research data on this disease are limited compared to other cancers (3–5). Esophageal cancer is characterized by its aggressive nature and dismal 5-year survival rate, which stands at only 18% (6). Recent advances in the identification of molecular markers specific to esophageal cancer have led to the development of novel targeted therapy approaches by targeting these markers (7–12). However, inhibitors have the potential to cause primary or acquired resistance in patients who receive these treatments (13–16). Furthermore, in esophageal cancer, a diverse array soluble immunosuppressive factors and cells with immunosuppressive properties can interfere with immune effector cells, thereby creating a distinct immunosuppressive microenvironment.

Multiple factors influence the outcome of multi-modality treatments. An individual tumor’s intrinsic features are crucial to shaping its immune microenvironment and affecting the effectiveness of immunotherapy in esophageal cancer (17). As our understanding of the tumor microenvironment deepens, we increasingly recognize the significance of immune cell subsets in tumor development and the identification of potential therapeutic targets. The microenvironment in esophageal cancer is complex, comprising of various components such as NK cells, tumor-associated macrophages, dendritic cells (DCs), myeloid-derived suppressor cells (MDSCs), neutrophils, mast cells (MCs), eosinophils, endothelial cells, tumor angiogenesis, and cancer-associated fibroblasts (CAFs) (18, 19). Extensive exploration has been conducted on the utilization of clinical immunotherapy approaches that target innate immune cells as adjuvant therapies in conjunction with surgical resection and chemoradiotherapy for the treatment of diverse cancers. The strategies encompass the utilization of immune checkpoint inhibitors and Chimeric Antigen Receptor T-Cell Immunotherapy (20, 21). Analyzing the characteristics of cells within the tumor microenvironment to predict immune infiltration is crucial for exploring new immunization strategies and studying responses to existing immune checkpoint inhibitors (22, 23). Recent research has categorized the microenvironments of tumors in cancer patients into three fundamental immune profiles: tumors that are immune inflamed (“hot”), immune excluded, and immune desert (“cold”). These profiles suggest different treatment options, excluding esophageal cancer, and provide valuable insights for effective therapeutic interventions (24, 25). To conclude, a meticulous and all-encompassing examination of the esophageal cancer tumor microenvironment, coupled with the determination of the corresponding tumor immunophenotype, can prove to be a valuable approach in directing immunotherapy and forecasting its effectiveness (20, 21, 23).

Several studies have substantiated the significant involvement of m^6^A modification in the development of tumor microenvironment (TME) diversity and complexity, a phenomenon that cannot be entirely elucidated by the RNA degradation mechanism (26). m^6^A modulators affected inflammation infiltrates and neovascularization of tumor tissues in human abdominal aortic aneurysm samples, where the markers METT14, FTO, and YTHDF3 are strongly colocalized with CD45+ leukocytes and CD3+ T cells, as well as CD68+ macrophages (27). Similarly, a METTL3/FTO-m^6^A methylation-mediated generation of M1/M2 macrophages from murine bone marrow-derived macrophages (BMDMs) has been described (28, 29). A new class of drugs targeting DNA methyltransferases (DNMTs) has been shown to successfully restore coordinated immune responses in solid tumors by triggering MHC 1 and interferon (IFN)-triggered immune-related signaling (30, 31). However, most studies, which are constrained by the state of technology, focus on just one or two m^6^A regulators, which is insufficient to describe the intricate functions of regulators in tumors. These research were made feasible by the ongoing development and collection of transcriptomics and genomic data, which offer a wealth of tools and resources for the study of m^6^A regulators and immune modulation (32).

In current study, we conducted a comprehensive analysis of m^6^A modifications and identified two distinct patterns of modifications, termed m^6^A clusters. These patterns were associated with different survival advantages and exhibited characteristics relevant to the TME, immune cell infiltration, and transcriptome (33). The observation that the TME characteristics linked with each m^6^A modification pattern closely corresponded with the manifestations and features of immune exclusion and immune inflammation, respectively, was of significant interest. This indicates a significant influence of m^6^A modification on individual tumor microenvironments (34, 35). Furthermore, a scoring system was devised to evaluate singular m^6^A modifications, facilitating the prediction of prognosis and the efficacy of immunosuppressive therapy. The strong correlation between m^6^A modifications and TME immune cell infiltration suggests that these modifications could serve as important prognostic markers and guide immunotherapy decisions in esophageal cancer.

## 2 Materials and methods

### 2.1 Esophageal cancer data acquisition and preprocessing

The **Supplementary Figure S1** depicts the workflow employed in this study. The esophageal cancer samples’ transcriptional and clinical feature data were obtained from Gene Expression Omnibus (GEO, https://www.ncbi.nlm.nih.gov/geo/) databases and The Cancer Genome Atlas (TCGA, 2022.12.01, https://portal.gdc.cancer.gov/). Two distinct cohorts of esophageal cancer (ESCA), namely TCGA-ESCA and GSE13898, were used for further analysis. The RNA-Seq data obtained from the TCGA cohort underwent additional processing, resulting in the conversion of the data into transcripts per kilobase (TPM). Retrieve the normalization matrix file from the GEO database and employ R’s SVA package to address batch effects across distinct datasets. Obtain the survival duration and survival outcome data of two cohorts, with the exclusion of samples with survival periods less than 31 days and incomplete survival information. The Cancer Genome Atlas database was utilized to obtain somatic mutations, and copy number variation data for esophageal cancer were obtained from the UCSC Xena database (http://xena.ucsc.edu/) (36).

### 2.2 Classification according to 23 m^6^A regulators

These regulators include eight writers (METTL3, METTL14, METTL16, RBM15, RBM15B, VIRMA, WTAP and ZC3H13), 13 readers (FMR1, HNRNPC, HNRNPA2B1, IGFBP1/2/3, LRPPRC, RBMX, YTHDC1/2 and YTHDF1/2/3), and two erasers (FTO and ALKBH5). These modulators have been reported to affect or modulate the performance of RNA (**Supplementary Figure S2**). The expression levels of these 23 m^6^A regulators were utilized for unsupervised clustering analysis to identify distinct subtypes of m^6^A methylation modifications and classify patients for further analysis. The consensus clustering technique, implemented with the R package ConsensusClusterPlus, was utilized to calculate the number of clusters and assess their stability (37, 38).

### 2.3 scRNA-seq data processing

We analyzed the dataset GSE196756 about Esophageal Squamous Cell Carcinoma (ESCC) cells from the GEO repository (39). The data were sourced from Homo sapiens, with the data platform being GPL24676. We picked specific ESCC samples (GSM5900215,GSM5900216,GSM5900217,GSM5900218,GSM5900219,GSM5900220) for analysis. The R package: “Seurat” was used to analyze the transcript count matrix for quality control and preliminary data exploration (40). The filtering threshold was set as follows: Excluding genes detected in less than 3 cells, excluding cells with < 200 genes detected, Excluding cells with > 20% mitochondrial gene expression. We addressed batch differences using LogNormalize, Harmony and Principal Component analysis (PCA) helped us cluster cells based on variable genes via Seurat’s “FindNeighbors” and “FindClusters” functions. Uniform t-distributed Stochastic Neighbor Embedding (t-SNE) helped visualize this.

### 2.4 Estimation of immune infiltrating cells in TME

The R software package GSVA was utilized to conduct GSVA enrichment analysis to look at variations in m^6^A modification patterns in biological processes. The GSVA technique is a nonparametric and unsupervised approach that is predominantly employed to assess alterations in the activity of biological processes and pathways within samples (41). The gene sets from “c2.cp.kegg.v7.2.symbols” were downloaded from the MSigDB database for performing GSVA analysis. The present study utilized Single Sample Gene Set Enrichment Analysis (ssGSEA) in the R software package GSVA to assess the infiltration rates of 24 immune cells across various m^6^A regulator clusters. The differences between different m^6^A regulator clusters were assessed using the Wilcox test, and survival analysis was conducted to examine their association with patient outcomes.

### 2.5 Gene expression differences among phenotypes modified with m^6^A

Using a consensus clustering algorithm, we were able to divide esophageal cancer patients into two distinct subtypes according to m^6^A regulator expression. We revealed that the relationship between the two m^6^A clusters and immune landscape by CIBERSORT, EPIC, MCPCOUNTER, QUANTISEQ, TIMER, and XCELL algorithms (42). The differentially expressed genes (DEGs) between these two m^6^A-modified clusters were subsequently identified using the Limma package. The significance criterion for determining differential genes was set at a p-value < 0.05.

### 2.6 Differentially expressed genes enriched in functional pathways and functions

An important bioinformatics tool for gene annotation and analysis is the Gene Ontology (GO). It encompasses three categories: cellular component (CC), biological process (BP), and molecular function (MF). The Kyoto Encyclopedia of Genes and Genomes (KEGG) database serves as an integrative platform for genomic, chemical, and system function data, enabling the correlation of gene catalogs with higher-level system functions across various levels, including the cell, species, and ecosystem. To annotate the DEGs and gain insights into their biological functions, we utilized the clusterProfiler package, a widely used R package for functional enrichment analysis. The clusterProfiler package offers convenient functions to perform GO and KEGG enrichment analyses. For the study to be meaningful, the p-value must be less than 0.05 and the q-value must be less than 0.05.

### 2.7 The construction of the m^6^A score

We created a scoring system for m^6^A based on PCA to measure the patterns of m^6^A change in specific esophageal cancer patients. Genes demonstrating significant prognostic effects were selected from the different m^6^A modification clusters, based on which clustering of samples and construction of m^6^A scores were performed using a univariate Cox regression model. We determined the number of gene clusters and ensured stability using the consensus clustering algorithm. Marker scores for m^6^A-related genes were generated using PCA, and the first and second principal components were extracted as the marker scores. The method emphasizes the scores based on the collective behavior of highly correlated or inversely correlated genes within significant gene clusters, while minimizing the impact of genes that do not align with other members of the cluster. PCA is a dimensionality reduction method typically used to reduce the dimensionality of a dataset by transforming a large number of variables into fewer variables that still contain most of the information in the set (43, 44). We used the following method to define m^6^A scores: m^6^Ascore = Σ(PCA1i + PCA2i), the variable “i” denotes the final gene expression linked to the m^6^A phenotype (45, 46).

### 2.8 Evaluate the m^6^A scoring model

To evaluate the clinical applicability and reliability of the m^6^A score, receiver operating characteristic (ROC) curves were utilized to predict the outcomes at 1 year, 3 years, and 5 years. Initially, the ROC curve was constructed using all samples, followed by a separate analysis focusing on the TCGA-ESCA cohort to compare the prognostic predictive performance of the m^6^A score against other clinical variables. Correlations between the m^6^A score, clinical variables, and prognosis were examined using both univariate and multivariate Cox regression analyses. The purpose of the study was to examine the potential of the m^6^A score as a standalone predictive marker for esophageal cancer. Significance at the p < 0.05 level is usually used to determine statistical significance in a forest plot diagram. Furthermore, a nomogram was constructed using eight indicators (age, gender, tumor grade, stage T, N, M, pathologic stage, and m^6^A score) to anticipate the patient’s 1-, 3-, and 5-year survival rates. The predictive performance of the nomogram was evaluated using ROC curves. The R packages timeROC, rms, survival and survminer were employed for the necessary calculations and graphical representation.

### 2.9 Data research on genome mutations

The frequency of copy number variation (CNV) for the 23 m^6^A regulators in the TCGA-ESCA cohort was computed to assess the occurrence of copy number increases or losses. Copy number variation plots were generated using the R package Circos to visualize CNV patterns of m^6^A regulators of human chromosomes. The Tumor Mutation Burden (TMB) was computed by quantifying the aggregate count of nonsynonymous mutations present in the TCGA-ESCA cohort. The R package maftools was employed to create an oncoprint, which visually represents the gene mutation landscape. Using these approaches, the copy number variation map and oncoprint provide insights into the copy number alterations and mutation profiles of the m^6^A regulators in esophageal cancer based on the TCGA-ESCA cohort.

### 2.10 Tumor immune dysfunction and exclusion prediction and IC50 estimation

The Tumor Immune Dysfunction and Exclusion (TIDE) model, developed by researchers (47, 48), is used to evaluate the clinical efficacy of immune checkpoint inhibition therapy. The TIDE model provides prediction scores that reflect the likelihood of a patient’s response to immune checkpoint inhibition. Higher TIDE prediction scores are associated with a poorer response to immune checkpoint inhibition therapy. This model helps clinicians and researchers assess the potential effectiveness of immune checkpoint inhibition in individual patients.

### 2.11 Collect critical information for ICI-based cohorts

A systematic search was conducted to identify publicly available gene expression profiles of patients undergoing immune checkpoint inhibitor (ICI) therapy. The search aimed to identify datasets that included detailed clinical and pathological information. Ultimately, three immunotherapeutic cohorts were included in our study: metastatic melanoma patients treated with nivolumab (anti-PD-1 monoclonal antibody) (49) or ipilimumab (anti-CTLA-4 monoclonal antibody) (50), and patients who have been diagnosed with metastatic urothelial carcinoma (mUC) and have received treatment with the anti-PD-L1-targeting drug Atezolizumab (51). The ESCA-specific immunotherapy-treated cohort GSE165252 was found (n=45 ESCA patients treated with anti-PD-1 monoclonal antibody Atezolizumab), which contains the binary information on immune therapy response (response and non-response groups). We curated gene expression profiles from pre-therapy biopsy samples and transformed them into TPM (Transcripts Per Million) format. These datasets provide valuable information for our study on the response to ICI therapy and associated gene expression patterns.

### 2.12 Sensitivity analysis of anticancer drugs

For the study of molecular therapies for cancer and gene mutations, relevant data from the Genomics of Cancer Drug Sensitivity (GDSC) database were downloaded (52). This database offers a valuable resource for studying drug sensitivity in various cancer types. We utilized the pRRophetic package to obtain cell line gene mutation data and IC50 values associated with various anticancer drugs from GDSC, allowing us to analyze the correlation between patients with high- and low-risk scores and their sensitivity to different anticancer drugs. Through this analysis, we were able to examine the correlation between patients exhibiting high- and low-risk m^6^A scores and their responsiveness to a diverse array of anticancer medications (53). By leveraging these resources, we aimed to gain insights into the association between m^6^A modification patterns and the response to specific anticancer therapies, further enhancing our understanding of personalized cancer treatment approaches.

### 2.13 Cell transfection and cell line establishment

Esophageal carcinoma cell lines KYSE510 and TE-1 were cultured in RPMI 1640 medium supplemented with 10% fetal bovine serum (FBS) at a temperature of 37 degrees Celsius and under a 5% CO_2_ atmosphere. In preparation for cell transfection, these cell lines were seeded into 6-well culture plates and incubated overnight to allow for attachment and initial growth. On the subsequent day, once the cells had reached a confluence of 20%-30%, transfection was performed with siRNAs at a final concentration of 50 nmol/L using Lipofectamine 2000 (Invitrogen), following the protocol provided by the manufacturer.

### 2.14 Explore and validate potential oncogenic functions of RBMX In ESCA

Supplemental Experimental Procedures include the following information: Western blot for protein expression, Plate clone formation assay, EdU assay for cell proliferation detection, Wound healing assay for assessing cellular migration, Transwell migration/invasion assay (**Supplementary Data Sheet S1**: Supplemental Experimental Procedures).

### 2.15 Statistical analyses

Statistical analyses and graphical representations were conducted using R version 4.3.1. The Wilcoxon rank sum test, a statistical method, is useful for assessing and contrasting dissimilarities between two groups. The correlation between m^6^A regulators and prognosis was assessed with univariate Cox regression models and Kaplan-Meier survival analysis. The selection of cutoff points for the m^6^A score was performed by repeatedly testing all possible cutoffs using the survminer package in R, aiming to identify the maximum rank statistic. Partition the samples into groups based on their m^6^A scores, with one group consisting of high scores and the other of low scores. Prognosis was assessed using the Kaplan-Meier method, and the log-rank test was assessed for both cohorts. At the same time, there are other statistical methods for targeted analysis. Heatmaps were generated using the pheatmap package in R. All statistical tests were two-tailed, and a p-value less than 0.05 was considered statistically significant.

## 3 Results

### 3.1 Mutation of m^6^A regulators, immune infiltration, and construction of the prognostic landscape

**Figure 1:**
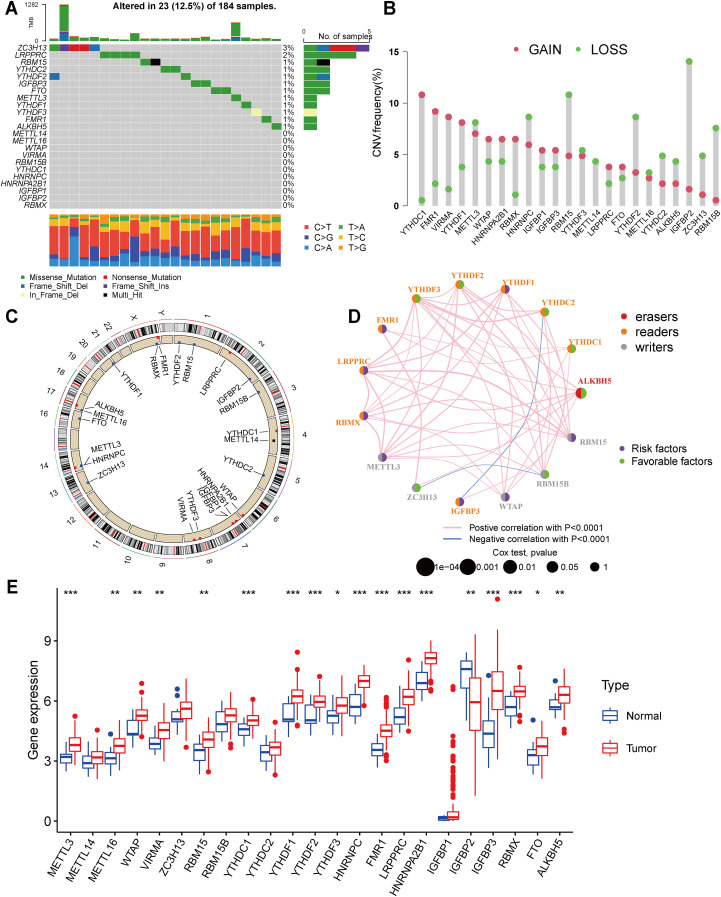
Landscape of genetic and expression variation of m^6^A regulators in esophagus cancer **(A)** Mutations of 23 m^6^A regulators in the TCGA-ESCA cohort. Each column represented individual patients. The upper barplot showed TMB, the number on the right indicated the mutation frequency in each regulator. The right barplot showed the proportion of each variant type. The stacked barplot below showed fraction of conversions in each sample. **(B)** In an TCGA-ESCA cohort, we looked at the CNV mutation rates of 23 m^6^A regulators. The findings were represented by red and green dots to represent increased and absent frequencies, respectively. **(C)** The precise chromosomal locations of CNVs in m^6^A regulators across all 23 chromosomes. **(D)** Interactions and prognostic implications of 23 m^6^A regulators in ESCA. The three types of m^6^A regulatory modifiers are represented by different colors: eraser in red, reader in orange, and writer in gray. The size of the circles corresponds to the prognostic relevance of each m^6^A modulator. The lines connecting the regulators indicate their interactions, with positive correlations in pink and negative correlations in blue. Prognostic risk factors are highlighted in purple, while prognostic protective factors are shown in green. **(E)** The expression of 23 m^6^A regulators between normal tissues and gastric tissues. Tumor, red; Normal, blue. The upper and lower ends of the boxes represented interquartile range of values. The lines in the boxes represented median value, and red and blue dots showed outliers. The asterisks represented the statistical p value (*P < 0.05; **P < 0.01; ***P < 0.001).

Our study included 23 m^6^A regulators. Firstly, we calculated the frequency of mutations in the 23 regulators in ESCA. The 23 m^6^A regulators exhibited low mutation frequencies, with only 23 (12.5%) out of 184 ESCA samples from the TCGA database showing genetic alterations. Mutation information for each gene in each sample was presented in the waterfall plot, with different colors and specific annotations at the bottom representing the different mutation types. Interestingly, the Oncoplot analysis revealed that ZC3H13 displayed the highest mutation rate, predominantly characterized by nonsense mutations, while YTHDC2 had a mutation frequency of 2% (**Figure 1A**).

Moreover, our analysis of copy number variations (CNVs) in the 23 m^6^A regulators highlighted the prevalence of CNV mutations in ESCA. Notably, YTHDC1, FMR1, VIRMA, YTHDF1, METTL3, WTAP, HNRNPA2B1, RBMX, HNRNPC, and IGFBP1 exhibited a high frequency of CNV amplification, while HNRNPC, RBM15, YTHDF2, IGFBP2, and RBM15B displayed extensive CNV deletions (**Figure 1B**). The chromosomal alterations associated with these CNVs are visually depicted in **Figure 1C**. In order to evaluate the influence of m^6^A regulators on patient prognosis, a Kaplan-Meier survival analysis was conducted. The findings demonstrated significant correlations between the prognosis of ESCA patients and 8 m^6^A regulators (**Supplementary Figure S3**). Additionally, seventeen modifiers with prognostic value in ESCA patients were identified using univariate Cox regression analysis (**Supplementary Table S1**). Furthermore, our analysis of the m^6^A network revealed the intricate interactions, connectivity, and prognostic significance of m^6^A regulators in ESCA (**Figure 1D**). Our findings indicate noteworthy correlations between the expression levels of m^6^A regulatory factors within the same functional class, as well as significant associations among the three distinct types of regulatory factors. This interplay is likely to contribute to the generation of distinct m^6^A modification patterns, which play a crucial role in the initiation and progression of cancer.

As well, we identified CNV alterations as a potential underlying cause of disrupted expression of m^6^A regulatory factors. To further investigate this, we compared the gene expression levels of the 23 m^6^A regulators between normal and tumor tissues (54). In ESCA tissues, m^6^A regulatory factors’ expression exhibiting CNV amplification (such as METTL3, WTAP, VIRMA, YTHDC1, YTHDF1, HNRNPC, FMR1, and HNRNPA2B1) was significantly higher than in normal esophageal tissues, while the expression of IGFBP2 was lower (**Figure 1E**). Collectively, these analyses underscore the noteworthy diversity in the genetic and expression profiles of m^6^A regulators detected between normal and ESCA specimens. These findings emphasize the critical role of dysregulated expression of m^6^A regulators in the development and progression of ESCA.

### 3.2 scRNA-seq analysis

**Figure 2:**
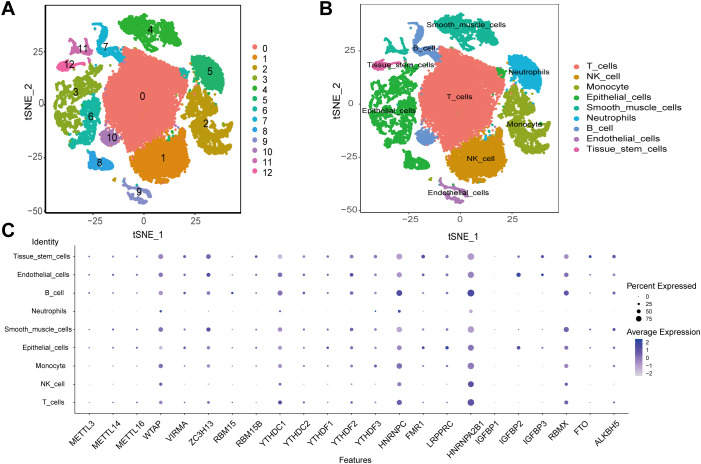
scRNA-seq data analysis. **(A)** The t-SNE algorithm divided the cells into 13 clusters by principal components. **(B)** The tSNE plot revealing 13 clusters was annotated into 9 different cell types. **(C)** The expression of 23 m^6^A regulators in 9 cell types.

Single-cell RNA sequencing (scRNA-seq) of 25,796 immune and 8,197 non - immune cells from three primary tumor and paired normal samples in ESCA patients was generated by using 10x Genomics platform. Before filtration, there were 33993 cells in the 6 ESCA samples. For GSE196756, counts were normalized and technical covariates (mitochondrial percentage) were regressed out using the LogNormalize method (default settings), and batch effects across samples (6 ESCA patients) were corrected for using Harmony with theta = 2 to preserve biological variance (55). We then performed data normalization and quality control, and finally selected the top 2000 highly expressed and variable genes for further analysis. PCA used to reduce the dimensionality of the data showed no clear tendency for cells to separate. Nonlinear dimensionality reduction was performed using the t-SNE algorithm, which successfully clustered the cells into 13 clusters (**Figure 2A**). We then annotated all clusters and identified 9 cell types (**Figure 2B**). Furthermore, the expression levels of 23 m^6^A modulators were most abundant in B cells and T cells (**Figure 2C**). WTAP, ZC3H13, YTHDC1, HNRNPC, HNRNPA2B1 and RBMX are expressed in most cell types.

### 3.3 Twenty-three regulator-mediated isoforms of m^6^A methylation

**Figure 3:**
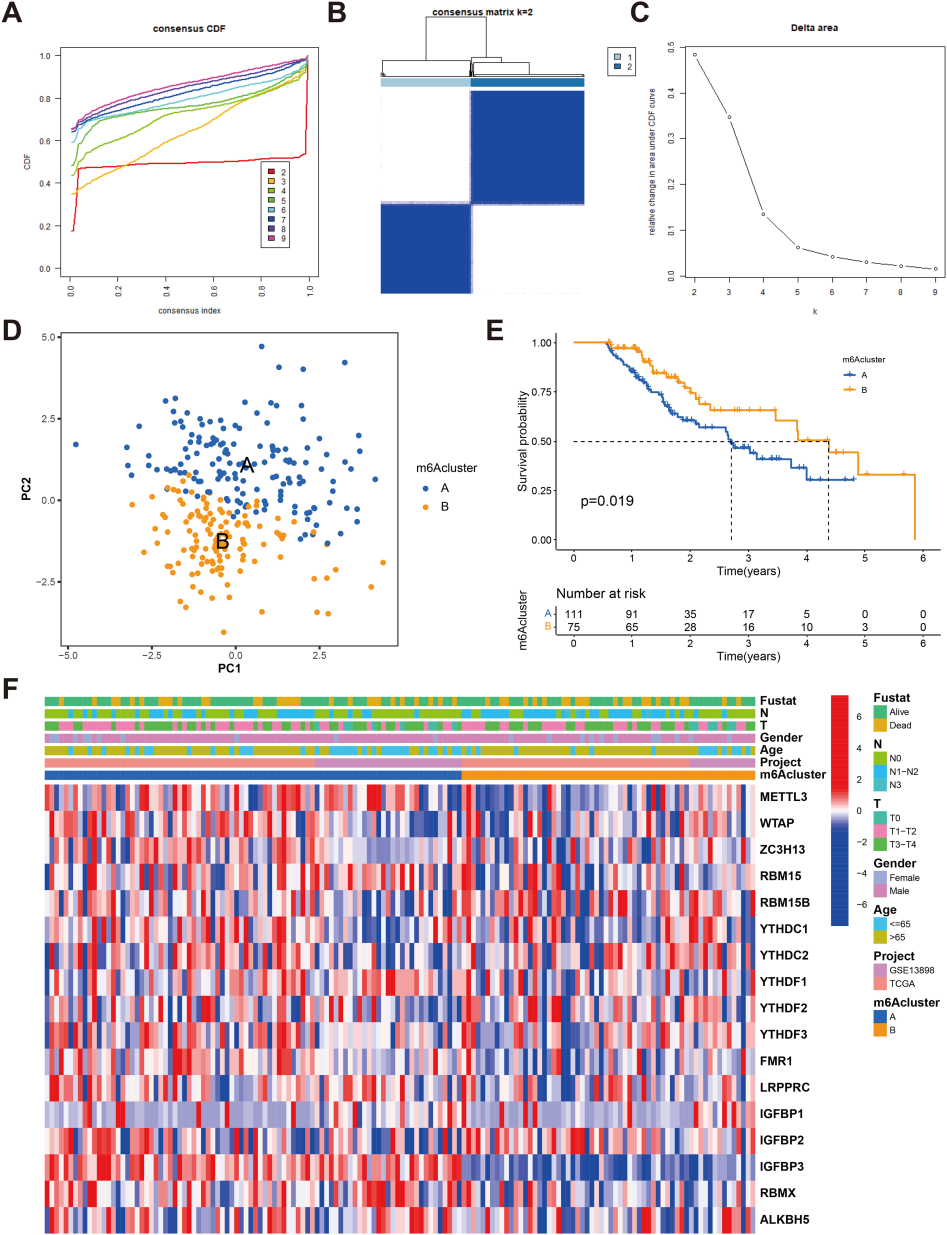
Identification of m^6^A methylation modification subtypes. **(A)** Consensus clustering and generated a CDF with the number of subtypes ranging from k = 2 to 9. **(B)** The heat map of sample clustering under k = 2 in 2 independent ESCA cohorts. **(C)** The relative change in the area under the CDF curve for values of k ranging from 2 to 9. **(D)** Principal component analysis of transcriptome profiles of two m^6^A modification patterns **(E)** Survival analyses for the two m^6^A modification patterns based on 186 patients with esophagus cancer from TCGA-ESCA and GEO cohorts (GSE13898) including 111 cases in m^6^Acluster-A, 75 cases in m^6^Acluster-B, Kaplan-Meier curves with Log-rank p value 0.019 showed a significant survival difference among two m^6^A modification patterns. The overall survival rate of cluster B in m^6^A cluster A and B subclusters is better. **(F)** Unsupervised clustering of 23 m^6^A regulators in two cohorts with heatmap analysis of m^6^A cluster, tumor stage, survival status, and age. Red is high expression, blue is low expression.

Using the ConsensusClusterPlus R software package, we performed patient classification based on the expression of the 23 m^6^A regulators to delineate distinct m^6^A-modified subtypes. Our analysis revealed two subtypes: subtype A consisting of 111 cases and subtype B consisting of 75 cases (**Figures 3A–D** and **Supplementary Table S2**). Notably, patients belonging to m^6^A regulator group B exhibited significantly longer survival compared to those in m^6^A regulator group A (P = 0.019, **Figure 3E**). We generated a heatmap to visualize the expression patterns of the 23 m^6^A regulators in the two m^6^A-modified subtypes (**Figure 3F**).

### 3.4 TME cell infiltration characteristics in distinct m^6^A modification patterns

**Figure 4:**
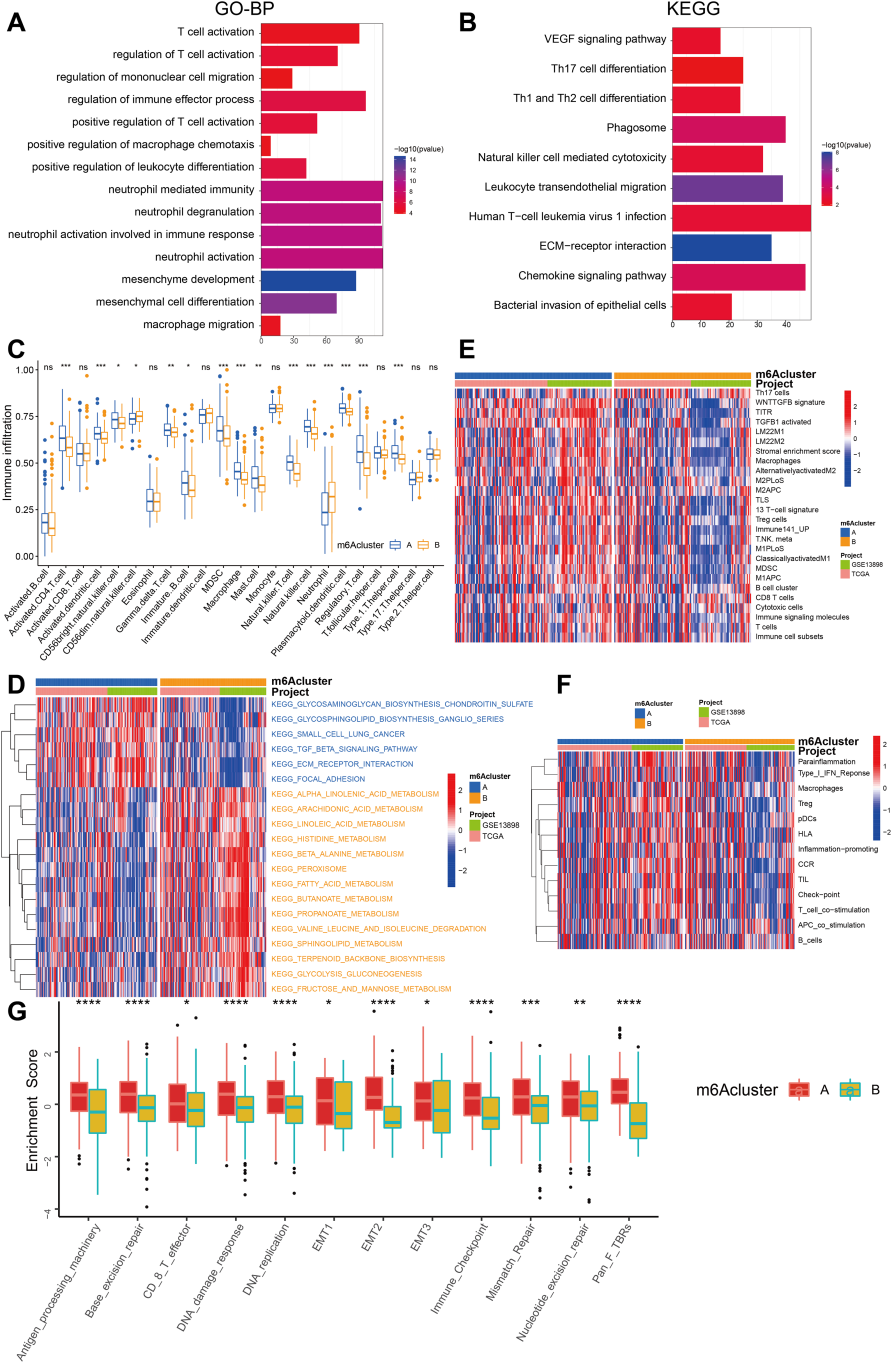
Comparison of the enrichment analysis for immune cells and immune pathways between two m^6^A clusters. **(A)** Functional annotation of the genes with different expressions between cluster A and cluster B using GO terms. **(B)** Pathway of KEGG enrichment of DEGs between two m^6^A clusters. **(C)** The abundance of each TME infiltrating cell in two m^6^A modification patterns. The upper and lower ends of the boxes represented interquartile range of values. The lines in the boxes represented median value, and the dots showed outliers. The asterisks represented the statistical p value (*P < 0.05; **P < 0.01; ***P < 0.001). **(D)** The heatmap was used to visualize these KEGG enrichment pathways, and blue represented activated pathways and yellow represented inhibited pathways. **(E)** The heatmap was used to visualize these immune cells. **(F)** The heatmap was used to visualize these immune-related pathways. **(G)** The box plot figure demonstrates the enrichment scores for clusters A (red) and B (yellow) across several biological processes, highlighting statistically significant differences (*P < 0.05; **P < 0.01; ***P < 0.001, ****P < 0.0001).

To gain insights into the underlying biomolecular signatures associated with the different m^6^A-modified phenotypes, we integrated the expression profiling data of both TCGA-ESCA and GSE13898 cohorts and performed differential expression analysis using the Limma R software package. This analysis identified 2599 DEGs, which were subsequently annotated using the clusterProfiler R package. The DEGs were found to be enriched in several important biological processes, including T cell activation, regulation of immune effector process, neutrophil-mediated immunity, mesenchyme development, mesenchymal cell differentiation, leukocyte transendothelial migration, chemokine signaling pathway, and VEGF signaling pathway (**Figures 4A, B**, **Supplementary Tables S3**, **S4**).

In order to examine the biological alterations linked to diverse m^6^A modification patterns, a comparative analysis of immune cell composition in the TME was performed. The findings indicate that the A subcluster exhibited a higher degree of infiltration by memory B cells, immature B cells, T helper 1 (Th1) cells, activated memory CD4+ T cells, and regulatory T cells (Treg). On the other hand, m^6^A cluster B exhibited significantly increased infiltration of natural killer cells and neutrophils (**Figure 4C**).

We further employed GSVA enrichment analysis to gain insights into the biological activity associated with these distinct m^6^A modification patterns. The results of our observations indicate that m^6^A cluster A exhibits noteworthy enrichment in pathways linked to stroma and cancer metastasis, including ECM-receptor interaction, focal adhesion, and others. On the other hand, m^6^A cluster B showed enrichment in metabolic pathways such as histidine metabolism, fatty acid metabolism, propanoate metabolism, glycolysis, fructose, and mannose metabolism (**Figure 4D**).

**Figure 5:**
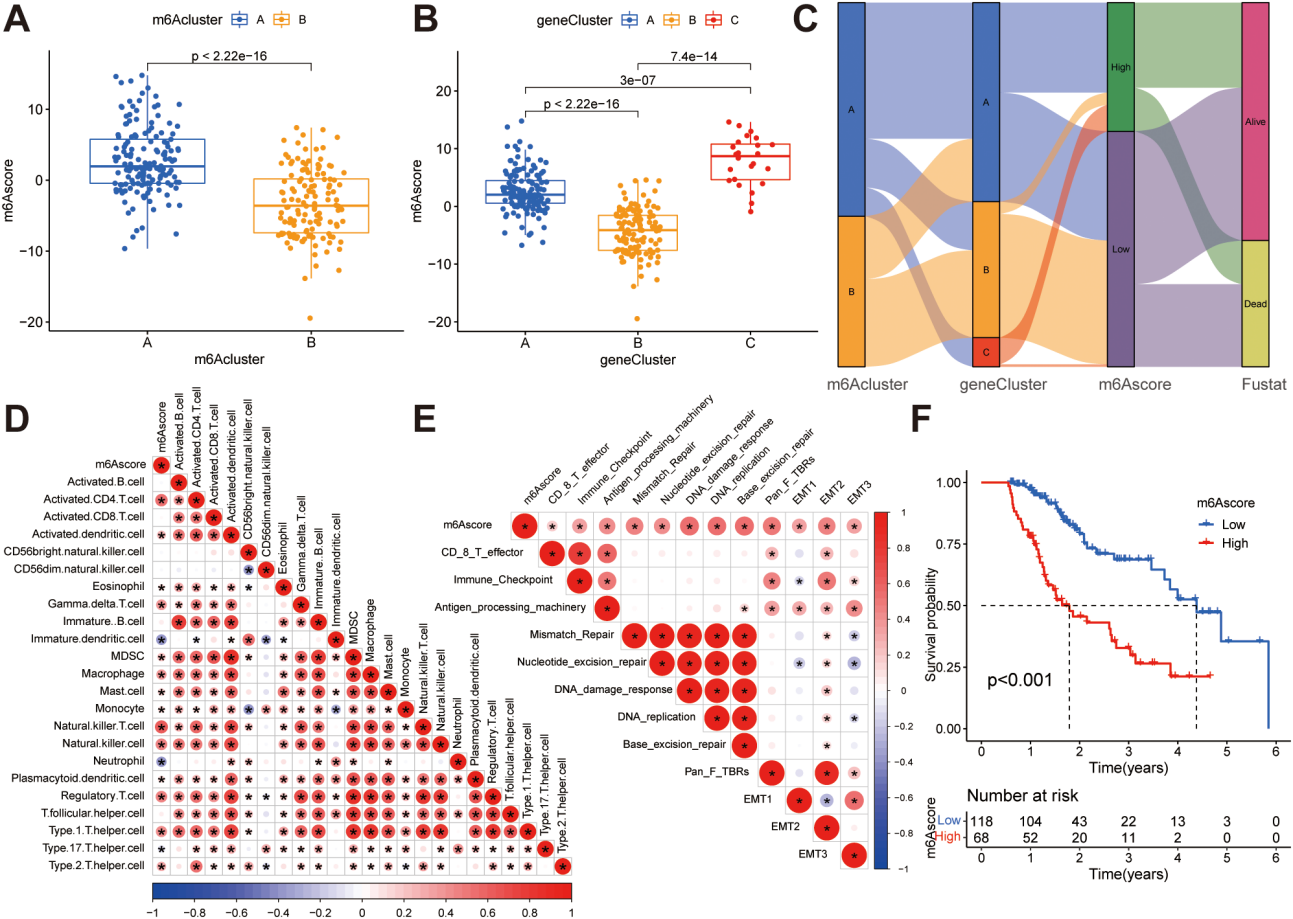
Construction of m^6^A score. **(A)** Differences in m^6^A scores between two m^6^A subclusters. **(B)** Differences in m^6^A scores between the three gene clusters. **(C)** The Sankey diagram illustrates the association between m^6^A score, m^6^A clusters, gene clusters, and survival outcomes. **(D)** Correlations between the m^6^A score and tumor-infiltrating immune cells using Spearman’s analysis. The positive and negative correlations are marked with red and blue, respectively. **(E)** Correlations between m^6^A score and the known biological gene signatures using Spearman analysis. The negative correlation was marked with blue and positive correlation with red. **(F)** Kaplan-Meier curve showing overall survival probability between high and low m^6^A score groups.

Interestingly, GSVA enrichment analysis revealed that m^6^A cluster A exhibited significant enrichment in adaptive immune cell infiltration, encompassing memory B cells, activated memory CD4+ T cells, immature B cells, Th1 cells, regulatory T cells (Treg), and stromal activation (**Figures 4C–G**, **5E**). Surprisingly, despite the higher immune cell infiltration, patients with this m^6^A modification pattern did not demonstrate a survival advantage (**Figure 4F**). Prior research has detected an immune-excluded phenotype within tumors, wherein immune cells exist in the stroma encircling nests of tumor cells, yet are unable to penetrate the tumor parenchyma. T-cell suppression is known to occur when the stroma in the TME is activated. Hence, our speculation is that the stromal activation observed in cluster A suppresses the antitumor effect of immune cells in patients with ESCA. The aforementioned conjecture was subsequently substantiated through analyses that demonstrated a marked increase in stromal activity within cluster A, which encompassed the activation of epithelial-mesenchymal transition (EMT), transforming growth factor beta (TGF-β), and WNT pathways, all of which were found to be statistically significant (**Figure 4G**).

We have integrated immune deconvolution tools such as CIBERSORT, EPIC, MCP_COUNTER, QUANTISEQ, TIMER and XCELL to distinct immune microenvironments characterize two m^6^A clusters (**Supplementary Figure S4**). Comparative analysis of immune infiltration patterns between the two m^6^A clusters revealed significant heterogeneity. Cluster A exhibited higher infiltration of immunosuppressive regulatory T cells (Tregs, P < 0.01 by CIBERSORT/QUANTISEO) and exhausted CD8+ T cells (PD-1+Tim-3+, P < 0.05), whereas cluster B showed elevated cytotoxic CD8+ T cells (Granzyme B+, P < 0.001 by TIMER). Pro-tumor M2 macrophages were enriched in cluster A (P < 0.001 across CIBERSORT/QUANTISEO/XCELL), while cluster B had higher M1 macrophages (P <0.05), suggesting divergent macrophage polarization states. CAFs were markedly increased in cluster A (P < 0.001 by EPIC/MCP-counter), correlating with elevated ECM remodeling scores (e.g., collagen cross-linking, P= 0.002). These findings were robust across multiple deconvolution algorithms (CIBERSORT, EPIC, XCELL).

Based on the comprehensive analyses conducted, it is intriguing to note that the two m^6^A modification patterns exhibit distinct characteristics in terms of TME cell infiltration. Cluster A is associated with an immune-excluded phenotype, characterized by the infiltration of adaptive immune cells and stromal activation. On the other hand, cluster B corresponds to an immune-inflamed phenotype, characterized by the infiltration of innate immune cells and metabolic reprogramming (**Figures 4C–G**). These findings suggest that m^6^A methylation modifications may be involved in tumor metabolism, EMT, immune regulation, and have close associations with tumor initiation and progression.

### 3.5 Characteristics of clinical and transcriptome traits in m^6^A-related phenotypes

**Figure 6:**
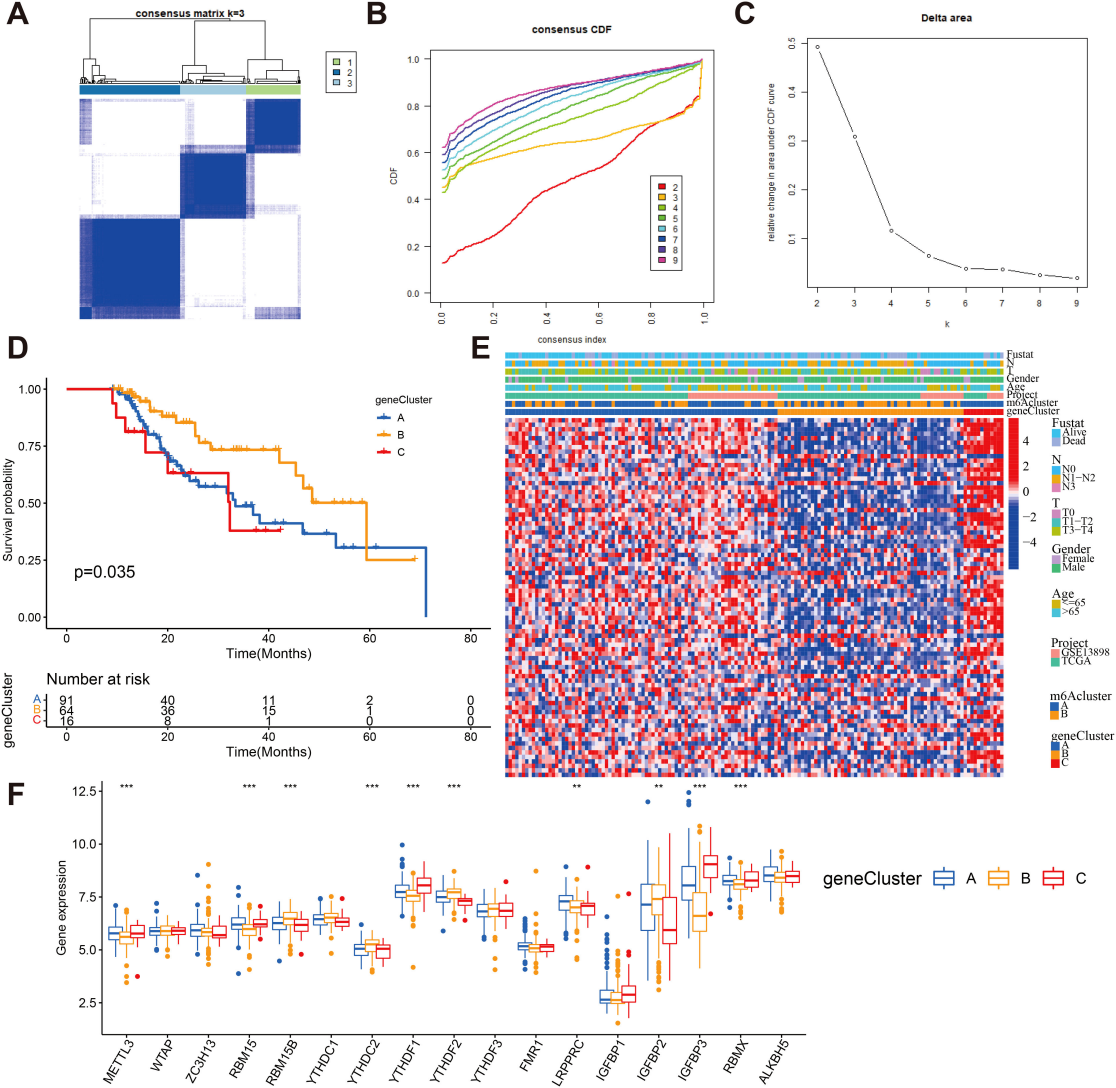
Consensus clustering of m^6^A -related gene subtypes **(A)** Consensus matrices of 80 m^6^A phenotype-related genes according to TCGA and GEO cohort for k = 3. **(B)** Consensus clustering CDF with the number of subtypes k = 2 to 9. **(C)** Consensus clustering cumulative distribution function k = 2 to 9. **(D)** Kaplan-Meier overall survival curves for patients in three m^6^A-related gene clusters. **(E)** Heatmap showing the correlation between the expression levels of the DEGs derived from 3 m^6^A clusters and sex, age, m^6^A clusters, tumor stage, survival status and gene clusters. Red is high expression, blue is low expression. **(F)** The expression of 23 m^6^A regulators in three gene cluster. The upper and lower ends of the boxes represented interquartile range of values (**P < 0.01; ***P < 0.001).

Despite the successful categorization of ESCA patients into two subtypes through a consistent clustering algorithm utilizing m^6^A regulator expression, the genetic alterations responsible for these phenotypes and their prognostic implications remain inadequately comprehended. To gain deeper insights, we conducted univariate Cox regression analysis on the 2599 DEGs identified between the previously established m^6^A clusters. A total of 80 survival-related genes were identified through this analysis, which we referred to as the m^6^A-related signature genes (**Supplementary Table S5**). Through unsupervised clustering analysis using representative m^6^A-associated marker genes, we identified three stable transcriptomic phenotypes, denoted as gene clusters A, B, and C (**Figures 6A–C**; **Supplementary Table S6**). The predictive importance of these gene subgroups was then investigated by fusing transcriptome data with survival data. Based on Kaplan-Meier analysis and log-rank test, it was observed that patients assigned to gene cluster B displayed a favorable prognosis (**Figure 6D**). A heat map was generated to visually depict the clinical characteristics of 80 m^6^A -related signature genes and the expression of m^6^A subgroups in the three gene clusters (**Figure 6E**). Notably, the three m^6^A gene clusters exhibited significant differential expression of m^6^A regulatory factors, which aligns with the methylation modification process and supports the predicted effects of m^6^A (**Figure 6F**).

### 3.6 Establishment of m^6^A score and its association with tumor microenvironmental features

While previous analyzes have yielded valuable insights into the impact of m^6^A methylation on immune cell infiltration status and tumor prognosis, accurate prediction of m^6^A methylation patterns in individual patients remains a challenging task. To address this challenge, the PCA score was employed to compute the m^6^A score, which also provides a quantitative assessment of the modified m^6^A landscape in patients with ESCA. **Figure 5A** illustrates that patients in m^6^A cluster B exhibit lower m^6^A scores compared to those in m^6^A cluster A, and **Figure 5B** demonstrates that patients in gene cluster B have lower m^6^A scores than those in gene clusters A and C. We have depicted the process of m^6^A score construction in a Sankey diagram (**Figure 5C**). In order to evaluate the association between the m^6^A score and tumor-infiltrating immune cells, a Spearman’s analysis was conducted and the outcomes were presented in a heatmap (**Figure 5D**), revealing a positive correlation between the m^6^A score and the presence of immune cells within the tumor microenvironment. Additionally, we examined the correlation between the m^6^A score and known signal pathway signatures. The resulting correlation matrix heatmap demonstrated that the m^6^A score exhibited significant positive associations with signatures related to EMT, stromal activity, DNA repair, antigen processing machinery, and the TGF-β pathway (**Figure 5E**). In addition, we conducted an evaluation of the prognostic relevance of the m^6^A score. Through implementation of the Kaplan-Meier survival analysis, it was determined that patients exhibiting low m^6^A scores experienced a more favorable prognosis in contrast to those with high m^6^A scores (**Figure 5F**). This indicates that the implementation of the m^6^A-score-based computation proficiently delineates the prognosis of patients.

In addition, we also found that in T0, T1-2, or T3–4 stage, N0, N1-2, or N3 stage, M0 or M1 stage, male or female, young or old patients, and patients, lower m^6^A score showed more significant survival advantage, which means that m^6^A score can also be used to access various clinical features of patients, such as age, gender, or clinical stage subgroup (**Supplementary Figure S5**).

### 3.7 Verification and clinical evaluation of m^6^A score

**Figure 7:**
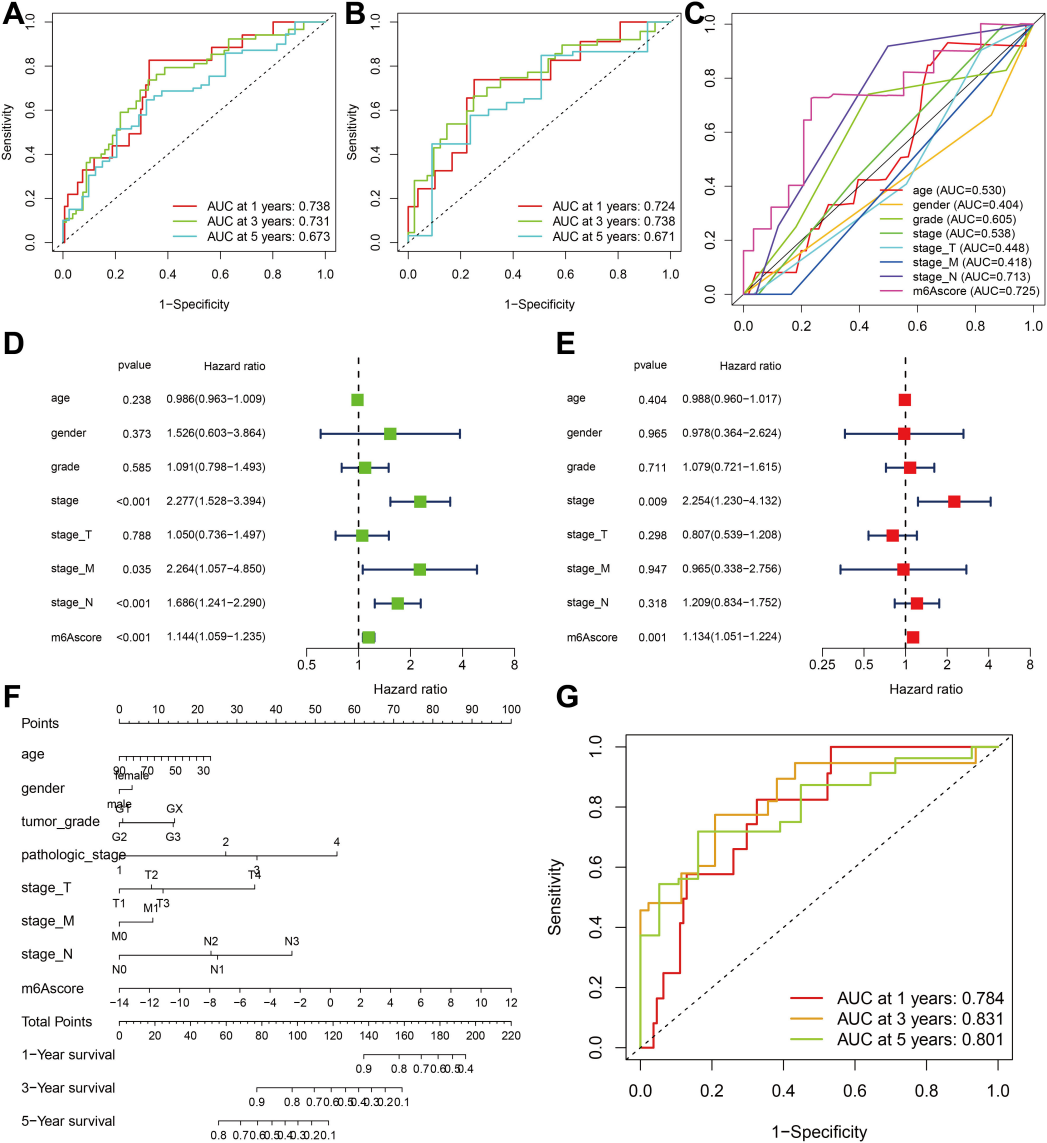
Verification and clinical evaluation of m^6^A score. **(A)** For all samples, the area under the curve (AUC) values of 1-, 3-, and 5-year ROC curves for m^6^A scores have been calculated. **(B)** For TCGA-ESCA cohort, the area under the curve (AUC) values of 1-, 3-, and 5-year ROC curves for m^6^A scores have been calculated. **(C)** Comparison of 1-year ROC curves of the m^6^A score model with other clinical features. **(D)** Univariate COX regression analysis of clinicopathological parameter and m^6^A score. **(E)** Multivariate Cox regression analysis of clinicopathological parameter and m^6^A score. **(F)** The nomogram is used to forecast the probabilities of 1-year, 3-year, and 5-year survival rates. **(G)** The AUC values of the nomogram’s 1-year, 3-year, and 5-year ROC curves.

To validate the m^6^A score, we conducted ROC curve analysis for 1-year, 3-year, and 5-year intervals and calculated the corresponding area under the curve (AUC) values. The results showed that all three ROC curves in the total sample cohort (**Figure 7A**) and the separate TCGA-ESCA cohort (**Figure 7B**) showed AUC values exceeding 0.67. Furthermore, when comparing the m^6^A score with other clinical features, the AUC value of the m^6^A score was found to be the highest (**Figure 7C**).

The findings of the univariate Cox regression analysis indicate that the stage, stage M, stage N, and m^6^A score possess prognostic potential (**Figure 7D**). Additionally, the multivariate Cox regression analysis reveals that both the stage and m^6^A score exhibit independent prognostic value (**Figure 7E**). To quantitatively assess individual risks in the clinical setting, the integration of multiple clinical indicators can be achieved through a nomogram. In this study, we developed a nomogram for predicting patients’ overall survival (OS) at 1-year, 3-year, and 5-year intervals (**Figure 7F**). The predictive performance of the nomogram was evaluated using ROC curve analysis. The present study determined the AUC values of the ROC curves for 1-year, 3-year, and 5-year intervals to be 0.784, 0.831, and 0.801, respectively (**Figure 7G**). These results indicate that the m^6^A score may serve as a promising clinical predictor and, when integrated with other clinical factors, could potentially improve the prognostic precision and clinical outcomes for patients diagnosed with ESCA.

### 3.8 Somatic variation correlates with m^6^A score

The potential of TMB as a tumor marker for immune checkpoint therapy in patients has been demonstrated. Given the clinical significance of TMB, an analysis was conducted to investigate the genetic characteristics within each subgroup, as defined by the m^6^A score, and their association with TMB. Patients were divided into two subgroups based on TMB.

**Figure 8:**
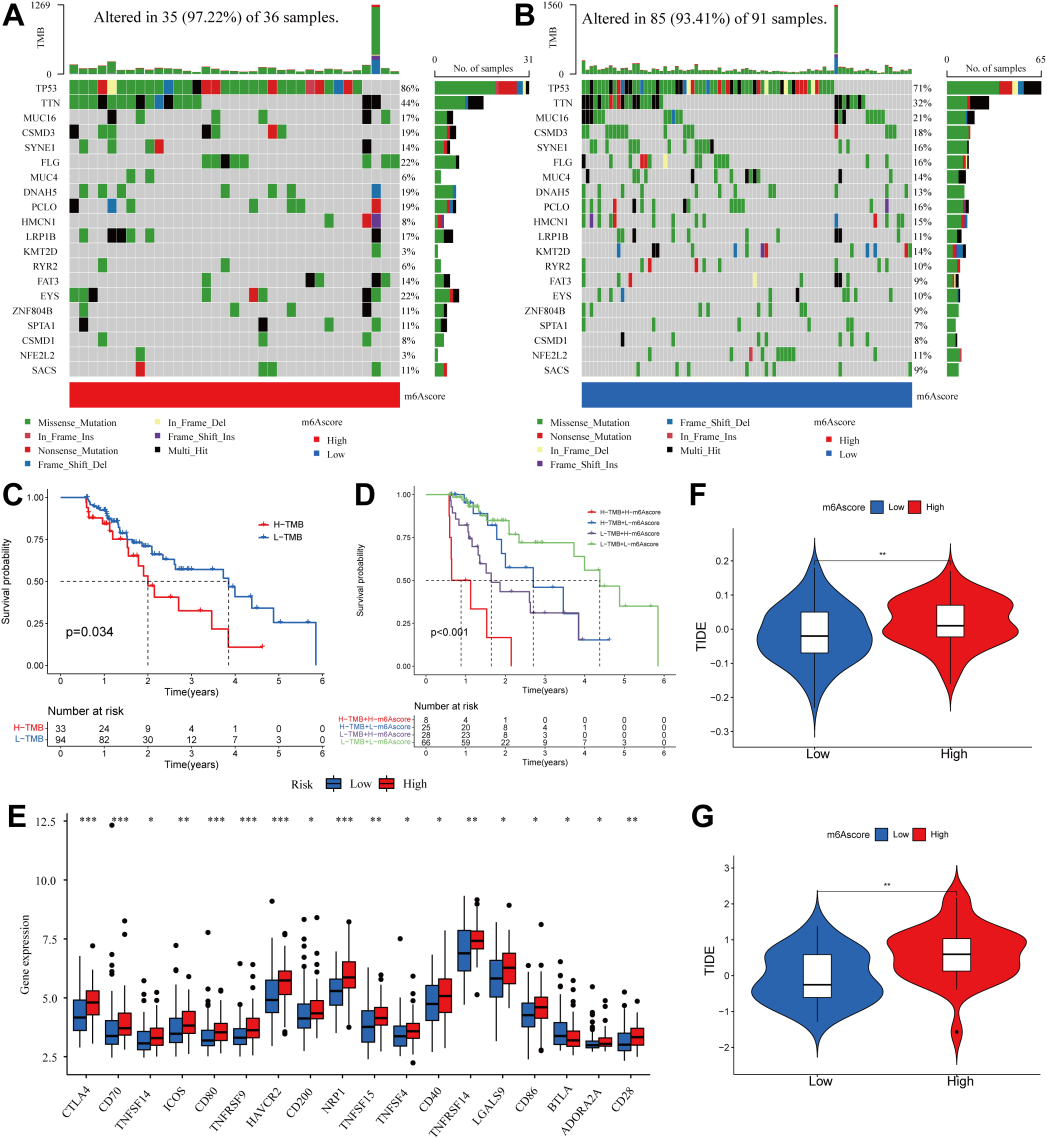
Correlation between m^6^A score and TMB and TIDE in the ESCA cohort. **(A, B)** OncoPrint for gene mutations in high and low m^6^A score groups. in the high m^6^A score group. **(C)** Kaplan-Meier curve showing overall survival probability between high TMB group and low TMB group (P < 0.05). **(D)** TMB and m^6^A scores were used in a stratified survival analysis. **(E)** Violin plot of differential expression of other immune checkpoints between groups with high and low m^6^A scores. (*P < 0.05, **P < 0.01,***P < 0.001) **(F)** TIDE differences in the TCGA cohort between high and low m^6^A score groups. **(G)** TIDE differences in the GSE13898 cohort between high and low m^6^A score groups.

Based on the results depicted in **Figures 8A, B**, it was observed that both TP53 (86% vs. 71%) and TTN (44% vs. 32%) exhibited a higher rate of somatic mutation in the group with a high m^6^A score, suggesting a potential association with the poorer prognosis observed in this group (**Figure 8C**). Subsequently, we assessed the combined prognostic value of these scores in stratifying ESCA patients. Survival analysis revealed that the TMB status did not influence predictions based on the m^6^A score, consistently demonstrating a survival advantage in the low m^6^A score group (**Figure 8D**). The results of this study contribute to a more thorough comprehension of the impact of the m^6^A score on genomic variability, presenting innovative perspectives for investigating potential associations between m^6^A methylation modification and somatic mutations. These findings demonstrated that distinct m^6^A modification patterns significantly influenced tumor immune phenotypes and may serve as predictive biomarkers for anti-PD-1/PD-L1 immunotherapy response efficacy. It has also been revealed that the m^6^A score is indirectly used to predict the success of immunotherapy.

### 3.9 M^6^A score predicts the possibility of benefit from immunotherapy

Subsequently, the differences in the levels of other immune checkpoints between the high and low m^6^A score groups were compared. The high m^6^A score group had higher expression of CTLA4, CD70, TNFSF14, ICOS, CD80, TNFRSF9, HAVCR2, CD200, NRP1, TNFSF15, TNFSF4, CD40, TNFRSF14, LGALS9, CD86, ADORA2A, and CD28, while the low m^6^A score group had higher expression of BTLA (**Figure 8E**).

The use of ICI therapy, specifically CTLA-4/PD-1 inhibitors, has resulted in a significant advancement in antitumor treatment. Alongside established predictors such as TMB, PD-L1, and MSI (56, 57), newly discovered indicators such as TIDE are extensively utilized and highly recommended for assessing immune response. Our analysis further demonstrated a noteworthy reduction in TIDE within the low m^6^A score group, as evidenced by the TIDE distribution in TCGA-ESCA and GSE13898 (both P < 0.01) (**Figures 8F, G**). As a result of these findings, it is inferred that tumor m^6^A modification patterns play an important role in mediating immune responses in tumors.

**Figure 9:**
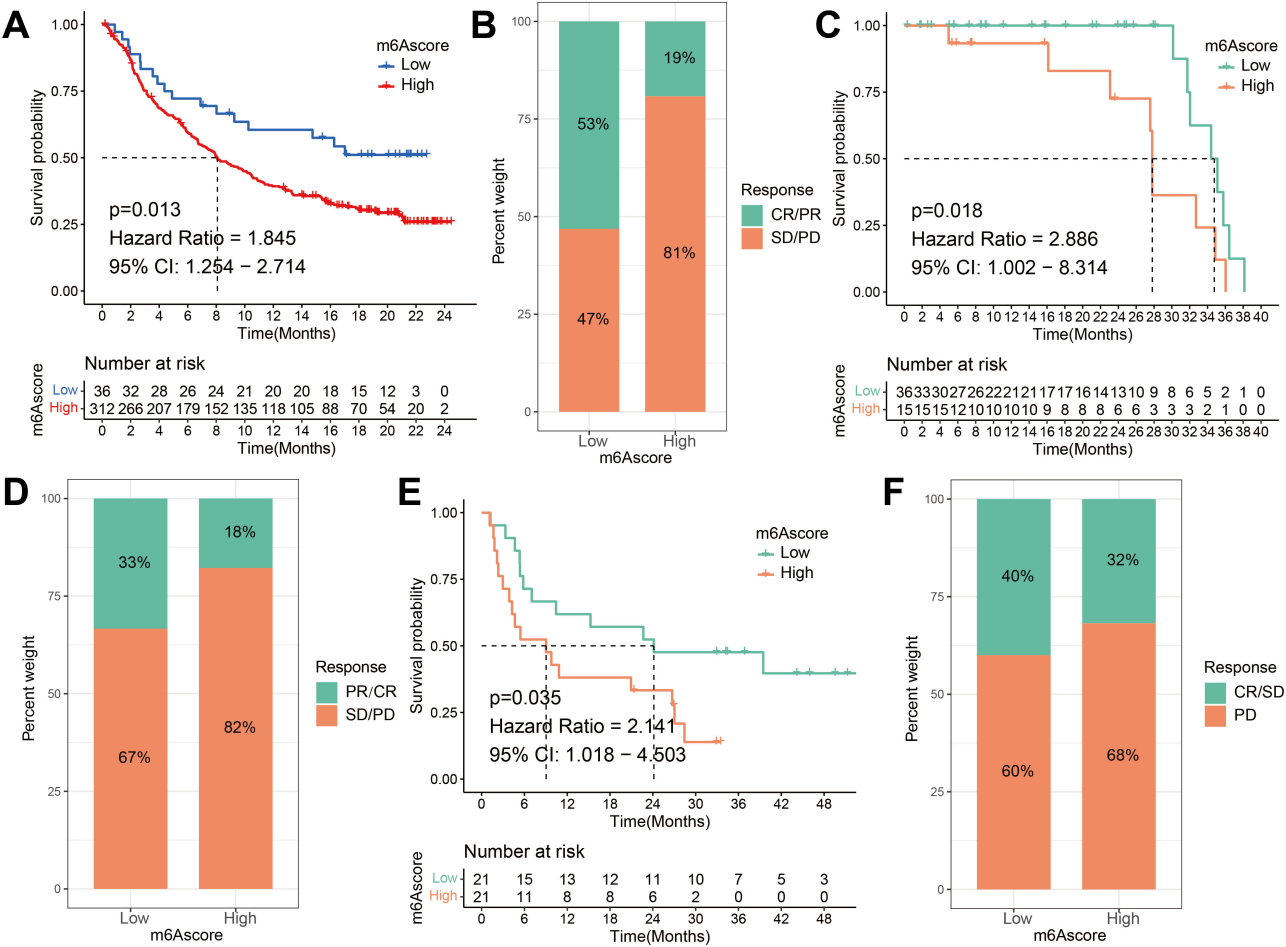
The m^6^A risk score predicts immunotherapeutic benefits. **(A)** Survival difference analysis of patients with high and low m^6^A risk score in the IMvigor210 cohort. P = 0.013. **(B)** Rate of clinical response to anti-PD-L1 immunotherapy in high or low m^6^A risk score groups in the IMvigor210 cohort. **(C)** Kaplan-Meier curves for high and low m^6^A risk score patient groups in the Riaz et al. cohort. Log-rank test, P = 0.018. **(D)** The fraction of patients with clinical response to anti-PD-1 immunotherapy (Riaz et al. cohort) in low or high m^6^A risk score groups. CR/PR vs. SD/PD: 33% vs. 67% in the low m^6^A risk score groups, 18% vs. 82% in the high m^6^A risk score groups. **(E)** Kaplan-Meier curves for high and low m^6^A risk score patient groups in the Vanallen et al. cohort. Log-rank test, P = 0.035. **(F)** The fraction of patients with clinical response to anti-CTLA-4 immunotherapy in low or high m^6^A risk score groups of Vanallen et al. cohort. CR/SD vs. PD: 40% vs. 60% in the low m^6^A risk score groups and 32% vs. 68% in the high m^6^A risk score groups. CR, complete response; PR, partial response; SD, stable disease; PD, progressive disease.

Based on the significant correlation between m^6^A scores and immune responses, our subsequent investigation aimed to assess whether m^6^A modification signatures could serve as predictive markers for patient response to ICI therapy in three separate immunotherapy cohorts. Firstly, a high m^6^A score exhibited significantly shorter survival time (HR, 1.845 [95% CI, 1.254 to 2.714], P = 0.013, **Figure 9A**) and a markedly clinical response in an anti-PD-L1 therapy in a cohort of metastatic urothelial carcinoma (response rate, low vs. high m^6^A score, 53% vs. 19%, **Figure 9B**) (51). This result was also identified in both the anti-PD-1 cohort (49) and anti-CTLA-4 cohort (50). Patients belonging to the high m^6^A score group demonstrated noteworthy clinical drawbacks and a considerably reduced lifespan (anti-PD-1, HR, 2.886 [95% CI, 1.002 to 8.314], P = 0.018. (**Figure 9C**) anti-CTLA-4, HR, 2.141 [95% CI, 1.018 to 4.503], P = 0.035, **Figure 9E**). The significant therapeutic benefits and immune response to ICI treatment were confirmed in patients with a low m^6^A score compared to those with a high m^6^A score (anti-PD-1, response rate, low vs. high m^6^A score, 33% vs. 18%, **Figure 9D**; anti-CTLA-4, response rate, low vs. high m^6^A score, 40% vs. 32%, **Figure 9F**). The m^6^A score of the GSE165252 cohort was further validated, the significant therapeutic benefits and immune response to anti-PD-1 treatment were confirmed in patients with a low m^6^A score compared to those with a high m^6^A score (anti-PD-1, response rate, low vs. high m^6^A score, 39% vs. 18%) (**Supplementary Figure S6**). The m^6^A score is also associated with patient response to immunotherapy and can be used to predict patients’ prognoses. In conclusion, the m^6^A score serves as a promising prognostic indicator in ESCA and may also provide guidance for ICI treatment in clinical practice.

### 3.10 Sensitivity analysis of patients with ESCA to different small molecule drugs based on m^6^A risk score

**Figure 10:**
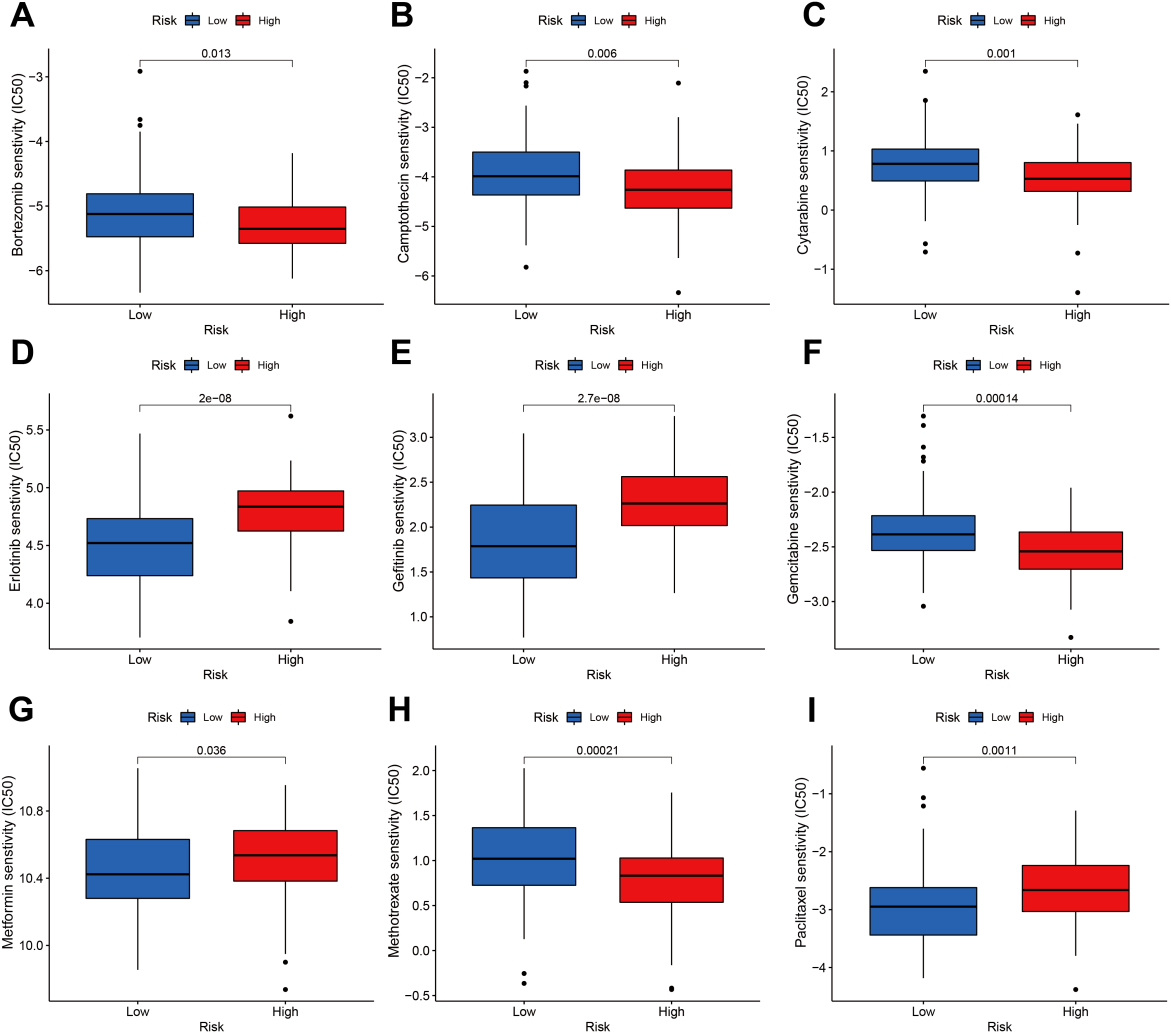
Sensitivity of the m^6^A risk score to different chemotherapy drugs and small molecule anticancer drugs was analyzed based on the GDSC database. **(A)** Bortezomib. **(B)** Camptothecin. **(C)** Cytarabine. **(D)** Erlotinib. **(E)** Gefitinib. **(F)** Gemcitabine. **(G)** Metformin. **(H)** Methotrexate. **(I)** Paclitaxel.

We performed an estimation of IC50 values and assessed the drug sensitivities of chemotherapeutic drugs for a cohort of 186 ESCA patients, utilizing data from the TCGA and GEO databases. The estimation process employed the “pRRophetic” R package, which utilized the expression profiles of the patients. Then, IC50 values were compared between the groups with high and low m^6^A scores. The IC50 values are utilized to assess the cellular response of various cell lines to a total of 138 distinct chemotherapeutic and small molecule anticancer drugs. The research found statistically significant differences (P < 0.05) between patients with high and low m^6^A risk scores in the IC50 values of several chemotherapeutic drugs and small molecule anticancer medicines. Notably, Bortezomib, Camptothecin, Cytarabine, Erlotinib, Gefitinib, Gemcitabine, Metformin, Methotrexate, and Paclitaxel exhibited particularly noteworthy differences (**Figures 10A–I**; **Supplementary Figure S7**).

### 3.11 RBMX’s impact on ESCC cell proliferation and migration

**Figure 11:**
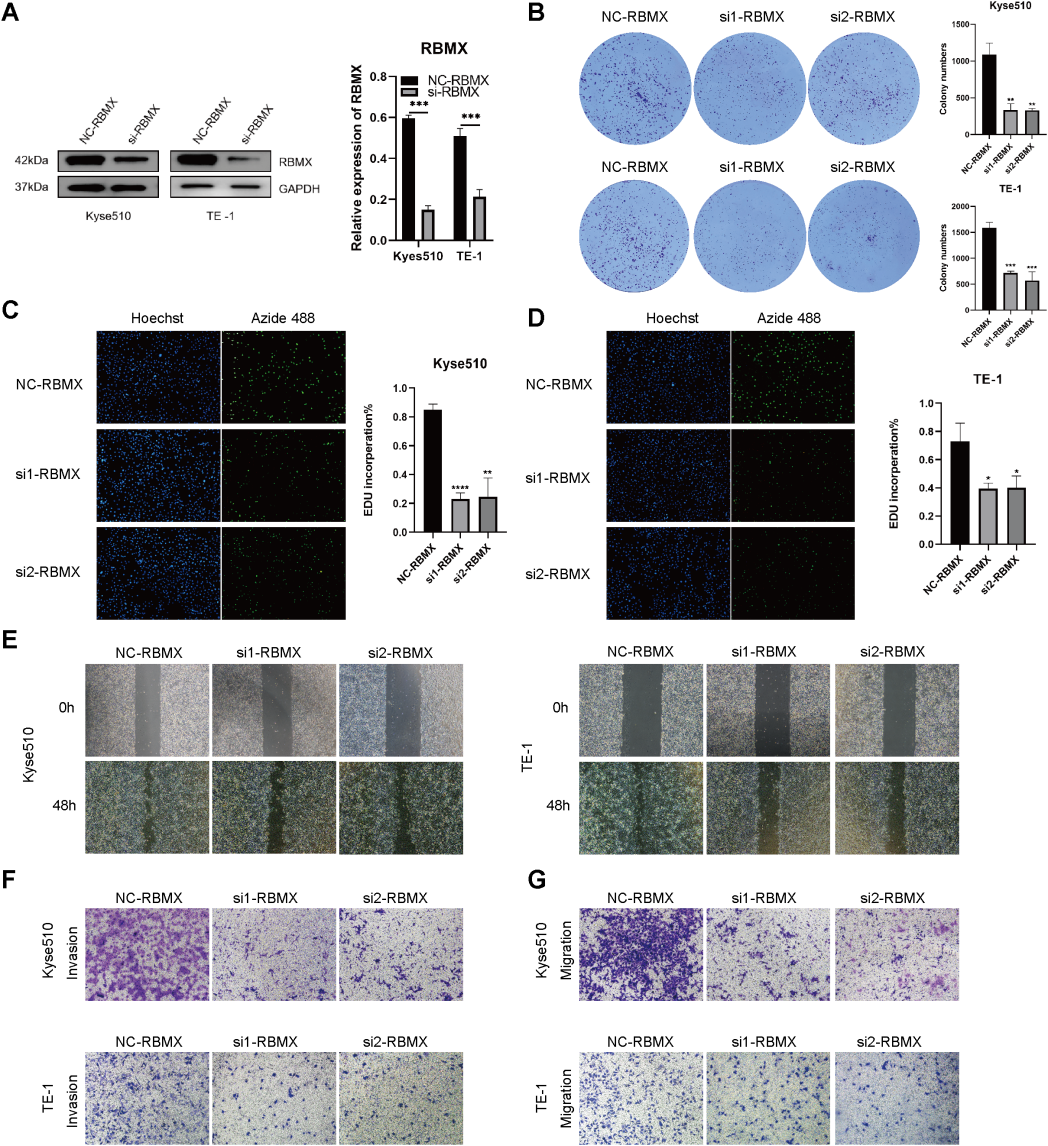
Impact of altered RBMX expression on proliferation and invasion of esophageal cancer cells. **(A)** RBMX protein expression levels in Kyse510 and TE-1 cells. **(B)** plate clone formation experiments assays in transfected Kyse510 cells and TE-1 cells. **(C)** EDU assays in transfected Kyse510 cells **(D)** and TE-1 cells. **(E)** Wound healing assays in transfected Kyse510 cells and TE-1 cells. **(F)** Invasion assay in transfected Kyse510 cells and TE-1 cells. **(G)** migration assay in transfected Kyse510 cells and TE-1 cells (*P < 0.05; **P < 0.01; ***P < 0.001, ****P < 0.0001).

To establish the mechanistic link between m^6^A modification and malignant progression in ESCC, we prioritized RBMX for functional interrogation based on its central position in the m^6^A regulatory network. Bioinformatics analysis identified RBMX as a hub gene in protein-protein-interaction network and co-expressed with key m^6^A regulators (METTL3, FTO, YTHDF2). RBMX expression levels were quantified in the ESCC cell lines KYSE510 and TE-1, revealing a notable reduction in protein expression following RBMX knockdown (**Figure 11A**). The clone formation assay demonstrated that the knockdown of RBMX significantly impeded the proliferative capacity of ESCC cells (**Figure 11B**). EDU staining corroborated these findings, indicating a significant decrease in the proliferative activity of si-RBMX-transfected KYSE510 and TE-1 cells (**Figures 11C, D**). The wound healing assay further illustrated that, after 48 hours, the wound closure ability of si-RBMX-transfected KYSE510 and TE-1 cells was markedly diminished compared to the Si-NC control group (**Figure 11E**). Additionally, migration and invasion assays were conducted to evaluate the impact of RBMX on ESCC cell motility. The knockdown of RBMX in KYSE510 and TE-1 cells led to a significant reduction in both the invasive and migratory capabilities of the cells (**Figures 11F, G**).

## 4 Discussion

Accumulating evidence from various studies emphasizes the important role of m^6^A methylation modification in the immune process of organisms. Further investigation is necessary to achieve a thorough comprehension of the immune cell infiltration within the TME in ESCA that is mediated by multiple m^6^A regulators. Therefore, it is crucial to clarify the characteristics of immune cell infiltration in relation to diverse m^6^A modification patterns. This will enhance our understanding of the TME and antitumor immune responses within it, and offer approaches for risk stratification and clinical management of patients with esophageal cancer. This study identified two distinct modification patterns with the assistance of 23 m^6^A regulators. The mRNA transcriptome differences observed between these patterns were found to be significantly associated with T cell activation, regulation of immune effector processes, neutrophil-mediated immunity, mesenchyme development, mesenchymal cell differentiation, leukocyte transendothelial migration, the Chemokine signaling pathway, and the VEGF signaling pathway (**Figures 4A, B**). The two patterns exhibited markedly different TME cell-infiltrating characteristics. Cluster A was categorized as an immune-excluded phenotype, marked by the infiltration of adaptive immune cells and stromal activation. On the other hand, cluster B was classified as an immune-inflamed phenotype, characterized by the infiltration of innate immune cells and metabolic reprogramming. The immune-inflamed phenotype, also known as “hot tumors,” is distinguished by substantial immune cell infiltration within the TME (24, 58, 59). Despite the presence of a significant number of immune cells in the immune-excluded phenotype, their distribution is limited to the stromal compartment surrounding the tumor cell nests, rather than infiltrating the tumor parenchyma. The stromal compartment may be localized to the tumor periphery or may extend into the tumor, potentially leading to the misinterpretation that immune cells are present within the tumor (60, 61). Consistent with the established definitions, our findings revealed that cluster A exhibited a pronounced stromal activation status, including elevated expression of EMT and TGF-β pathways (**Figures 4D, E, G**), which are associated with T-cell suppression. The observed TME cell-infiltrating characteristics in each cluster reinforce the validity of our immune phenotype classification based on distinct m^6^A modification patterns. Consequently, after comprehensively exploring the TME cell-infiltrating characteristics induced by distinct m^6^A modification patterns, it is not surprising that cluster A, despite having activated innate immunity, exhibited poorer prognosis.

The stromal activation in cluster A (e.g., TGF-β, EMT) and its link to immune exclusion are supported by recent studies on CAF subtypes (60–62). Cluster A exhibits an immunosuppressive stromal microenvironment owing to the enrichment of TGF-β and multiple EMT-related pathways and (**Figures 4D, G**). The tumor stroma, particularly CAFs and their remodeled extracellular matrix (ECM), plays a pivotal role in shaping the immunosuppressive TME by regulating T cell infiltration and function through both physical barrier and molecular mechanisms (60). The physical barrier prevents cytotoxic T cells from contacting cancer cells, creating an “immune-excluded” microenvironment (60). In addition to structural constraints, CAF heterogeneity further exacerbates immune evasion. Distinct CAF subpopulations may drive divergent stromal remodeling patterns: certain subsets promote the formation of rigid, cross-linked stroma that impedes T cell migration, while others secrete immunosuppressive factors (60, 61). Three functional subtypes of CAFs have been identified in non-small cell lung cancer (NSCLC) based on their heterogeneity (63). These functional disparities among CAFs are driven by their intrinsic TGF-β signaling. This CAF functional classification correlates with patients’ clinical responses to targeted therapies and is also associated with the tumor immune microenvironment (63). Notably, RNA modification “writers” (e.g., m^6^A/m^1^A regulators) appear to influence CAF activation states, as evidenced by the association between high “Writers-Score”, poor prognosis, and suppressive immune infiltration (e.g., M2 macrophages, EMT) (62). These findings suggest that epigenetic reprogramming of CAFs may reinforce immune exclusion by coupling matrix stiffness with broader immunosuppressive signals, such as PD-L1 upregulation. Thus, stromal activation drives CAF heterogeneity and immune exclusion via coordinated ECM remodeling (e.g., collagen cross-linking, fibronectin deposition) and epigenetic reprogramming (e.g., m^6^A-mediated RNA stabilization of TGF-β signaling components). Therapeutically targeting these matrix-driven immunosuppressive mechanisms—such as through ECM-degrading enzymes (e.g., collagenase) or epigenetic inhibitors—could dismantle the stromal-T cell barrier, thereby enhancing the efficacy of T cell–mediated antitumor immunity. The study by Du et al. elucidates that RBMX stabilizes IL-33 mRNA through a liquid-liquid phase separation mechanism, thereby activating the TGF-β signaling pathway. This process orchestrates the bidirectional regulation of tumor plasticity and the immunosuppressive microenvironment, providing a theoretical foundation for developing precision therapeutic strategies targeting the RBMX/TGF-β axis (64). We hypothesize that RBMX, as an m^6^A reader, regulates CAF crosstalk by enhancing RNA stability of the IL-33/TGF-β axis and increasing stromal stiffness, while simultaneously suppressing immune-activating signals (e.g., CXCL10-STAT1) to impair T cell function (61). This dual mechanism aligns with clinical observations in cluster A patients, where despite high immune cell infiltration, T cells are predominantly confined to stromal regions and exhibit significantly reduced survival rates. Such an “immune-excluded” phenotype closely mirrors the stroma-mediated immune privilege phenomenon proposed by Joyce et al. (60).

The immune landscape analysis underscores how m^6^A modification patterns shape tumor-immune interactions (**Supplementary Figure S4**). M^6^A cluster A (immune-excluded): Dominated by Tregs, M2 macrophages, and CAFs, this phenotype aligns with TGF-β-driven stromal activation. The concomitant suppression of cytotoxic lymphocytes (evidenced by low CD8+/NK cell ratios, P < 0.001) may explain poorer immunotherapy responses observed in this subgroup. M^6^A cluster B (immune-inflamed): Enriched for cytotoxic T/NK cells and immunostimulatory dendritic cells, this cluster demonstrates the potential of m^6^A modulation to overcome immune desertification. Notably, the M1/M2 macrophage balance (P < 0.001) mirrors metabolic reprogramming linked to m^6^A-regulated pathways.

The expression and function of m^6^A modulator genes in these cells may play an important role in regulating the tumor microenvironment. Especially in immune cells, the expression of m^6^A modulator genes may affect the function and activity of immune cells, thereby regulating tumor immune responses. Most of the 23 m^6^A modulator genes are distributed in epithelial cells, B cells, and T cells. As important components of the immune system, B cells and T cells play an important role in the tumor microenvironment. The expression level and functional status of m^6^A modulator genes may affect the activity, proliferation, migration and cell fate decisions of these immune cells, thereby affecting the efficacy of tumor immunotherapy. In addition, the expression of m^6^A modulator genes in tumor cells and epithelial cells may also directly affect tumor development and treatment response. Epithelial cells are often the cells of origin of tumors, and the expression of m^6^A modulator genes in these cells may regulate the proliferation, invasion, and metastasis capabilities of tumor cells. Recent advancements such as spatial transcriptomics and proteomics, exemplified by works utilizing techniques like spatial CITE-seq (65), multimodal tri-omics (66), and spatially resolved CRISPR screens (67), offer powerful methodologies for dissecting complex interactions within the tumor microenvironment. These technologies could provide novel insights into the spatial and functional dynamics of m^6^A methylation modifications and their impact on immune infiltration and cancer progression, potentially unveiling new therapeutic avenues.

M^6^A related characteristic genes were identified as DEGs associated with the prognosis of ESCA. By employing the m^6^A signature genes, we have successfully categorized the samples into three distinct subtypes of m^6^A -related genes, which exhibit significant associations with stromal and immune activations. Therefore, it is imperative to conduct a comprehensive evaluation of m^6^A modification patterns to enhance our understanding of TME cell infiltration characterization. To mitigate inter-individual variations, quantification of the m^6^A modification pattern among m^6^A -modified tumors is necessary. To this end, we have devised a set of scoring systems, referred to as the m^6^A gene signature, to evaluate the m^6^A modification pattern. The m^6^A modification pattern associated with the immune-excluded phenotype demonstrated a higher m^6^A score, whereas the immune-inflamed phenotype exhibited a lower m^6^A score.

Our findings align with previous studies on the TME, supporting the notion that m^6^A methylation modifications play a vital role in influencing distinct immune properties within the TME. Scoring models constructed using specific biomarkers modified by m^6^A have been successfully used in gastric cancer and colorectal cancer, providing improved clinical treatment selection and prognosis assessment for cancer patients (62, 68, 69). The findings suggest that the m^6^A score possesses the capacity to serve as a comprehensive metric for assessing the m^6^A modification pattern of individual tumors, and may be employed in the investigation of tumor immunophenotype and TME immune cell infiltration. Additionally, the validation of the m^6^A score through the TCGA-ESCA cohort highlights its considerable potential as a prognostic indicator for patients afflicted with ESCA. The nomogram, incorporating the m^6^A score along with other clinical variables, demonstrated effective predictive capabilities for patient prognosis.

Furthermore, our m^6^A score demonstrated a superior predictive capability in the context of immunotherapy for esophageal cancer. These findings were robustly corroborated in the IMvigor210 cohort, as well as in cohorts receiving anti-PD-1 and anti-CTLA-4 treatments, where the immune phenotype had been established (49, 50, 56). We could also predict the efficacy of adjuvant chemotherapy and the patients’ clinical response to anti-PD-1/PD-L1 immunotherapy through the m^6^A score.

The evaluation of genes that may drive mutations in tumors is an essential method for exploring the fundamental mechanisms of tumorigenesis and progression. Furthermore, it contributes to the rational selection of cancer diagnosis and treatment strategies. In our study, we observed a significant increase in the mutation rates of TP53 and TTN in the high m^6^A score group. TP53 mutations are prevalent in various cancer types and play a critical role in cancer progression. Loss or mutation of TP53 in cancer cells can disrupt T cell recruitment and impair T cell activity, aiding immune evasion and accelerating cancer growth in the process. Research on esophageal cancer has revealed that the absence of TP53, which encodes the P53 protein, Consequently, there is an augmentation of regulatory T cell (Treg) infiltration in both paracancerous and intratumoral tissues (70). On the other hand, TTN mutations have been associated with poor immune infiltration and worse prognosis in liver hepatocellular carcinoma, colorectal cancer, and ovarian serous cystadenocarcinoma (71–73). Notably, TTN mutations are frequently detected in solid tumors and have been correlated with increased TMB. Moreover, TTN mutations have been found to be associated with the objective response to immune checkpoint blockade (ICB) therapy (74).These findings highlight the potential impact of TP53 and TTN mutations in modulating the immune response within the tumor microenvironment and their relevance to clinical outcomes. Understanding the role of these mutations in tumor biology can provide valuable insights for the development of targeted therapies and immunotherapeutic strategies in cancer treatment.

The study elucidates the role of RBMX in ESCC, focusing specifically on its impact on cell proliferation and migration. The findings suggest that the expression levels of RBMX are critical for the malignant behavior of ESCC cells. In the KYSE510 and TE-1 ESCC cell lines, significant reductions in RBMX protein expression were observed following knockdown. This indicates that RBMX may play a crucial role in maintaining the cancerous state of these cells. The plate colony formation assay revealed that RBMX knockdown significantly impaired the proliferative capacity of ESCC cells, highlighting its potential as a therapeutic target. EDU staining, which assesses DNA synthesis during the S phase, confirmed the reduced proliferative activity in cells with lower RBMX expression. These findings support the hypothesis that RBMX is a key regulator of cell cycle progression in ESCC. The wound healing assay demonstrated that RBMX knockdown significantly diminished the wound closure ability of ESCC cells, underscoring its role in cell migration, which is crucial for cancer invasion and metastasis. Migration and invasion assays further indicated significant reductions in both the invasive and migratory capabilities of ESCC cells following RBMX knockdown. These observations suggest that RBMX is central to ESCC cell motility, a key factor in the metastatic spread of cancer. In summary, this study provides evidence that RBMX has multiple influences on ESCC, impacting both cell proliferation and migration. These findings indicate that RBMX may serve as a promising target for therapeutic intervention in ESCC. Additional research is needed to elucidate the molecular mechanisms through which RBMX exerts its effects and to investigate the potential of RBMX-targeted therapies for treating ESCC.

The role of RBMX in tumors is highly tissue-specific. In hepatocellular carcinoma (HCC) and T-cell lymphoma, elevated RBMX expression enhances tumor progression and chemoresistance by stabilizing oncogenic long non-coding RNAs (lncRNAs), such as BLACAT1, or modulating RNA metabolism (75, 76). In contrast, in bladder cancer, RBMX exhibits an oncogenic effect by inhibiting hnRNP A1-mediated PKM splicing (77). This paradox indicates that the function of RBMX may rely on the tissue-specific expression of its interacting partners, such as hnRNP A1 and specific lncRNAs. RBMX has been linked to chemoresistance in both T-cell lymphoma and small-cell lung cancer (75, 78), suggesting that it may affect treatment responses in esophageal cancer, particularly in platinum-resistant ESCC, by modulating DNA damage repair and apoptotic pathways, such as those involving the BCL2 family.

Research by Tuersun and Bei has emphasized that RBMX is a significant prognostic biomarker in various cancers, including esophageal cancer, where its expression correlates with tumor progression and poor clinical outcomes (79, 80). Investigating how RBMX influences alternative splicing and m6A methylation, particularly in relation to other RNA-binding proteins such as TRA2A, may reveal new insights into the biology of esophageal cancer and resistance to therapies like sorafenib (80). The interaction of RBMX with splicing factors such as TRA2A and hnRNP A1 offers deeper insights into the regulatory networks governing esophageal cancer progression. RBMX’s role in m^6^A methylation may contribute to the dynamic regulation of oncogenic lncRNAs, thereby influencing tumor biology. Future investigations should examine the mechanistic pathways by which RBMX influences alternative splicing and m^6^A modification across a broader range of cancers. Longitudinal studies are needed to assess its prognostic value over extended periods.

Our research has several limitations that should be acknowledged. Firstly, although we included 23 well-known m^6^A regulators reported in the literature, the significance of incorporating recently identified regulators to enhance the precision of m^6^A methylation pattern identification is incontrovertible. Incorporating additional regulators into the model can potentially improve the comprehensive understanding of m^6^A modifications. Secondly, while immunotherapy has shown significant benefits for some patients with low m^6^A scores, it is important to recognize that not all patients with low scores derive equal benefit. To enhance the predictive accuracy, it would be valuable to integrate additional clinicopathological features into the analysis. By incorporating these features, we can better identify patients who are more likely to respond favorably to immunotherapy. Thirdly, although we obtained a relatively large sample size of 186 ESCA patients from various cohorts, it is important to acknowledge that a larger and independent prospective cohort of ESCA patients who have undergone immunotherapy is required to validate our findings. Prospective trials with a substantial patient cohort are required to provide a more definitive demonstration of the prognostic value of the m^6^A score in relation to the response to immunotherapy. Furthermore, our study focused on a holistic analysis of the tumor microenvironment without further distinguishing between tumor, immune, and stromal components. This lack of component-specific analysis may mask certain subtype-specific information, which is a limitation of our study. Future investigations should consider dissecting the tumor microenvironment into its individual components to gain deeper insights into the interactions and contributions of different cell types. Lastly, we primarily aimed to propose molecular subtypes associated with m^6^A methylation across the tumor microenvironment and subsequently develop a scoring system. Furthermore, clinical analysis revealed that the m^6^A score, when combined with other clinical indicators, can serve as a valuable adjunct to existing variables and effectively predict patient prognosis. Addressing these limitations through further research and validation will enhance the scientific significance and clinical applicability of our findings.

This study offers novel insights into the clinical application of immunotherapy, presenting potential implications for its use in the field. One potential avenue for the development of novel immunotherapy drugs or treatment strategies involves the modulation of m^6^A modification patterns through the targeting of m^6^A regulators or m^6^A -related marker genes. This approach may serve to reverse unfavorable immune cell infiltration in the tumor microenvironment, thereby converting immune cold tumors into hot tumors (81). These findings aid in the identification of distinct immune phenotypes, thereby enhancing our understanding of patient response to immunotherapy. This information may help with the clinical use of customized immunotherapy for the treatment of cancer (82). We also demonstrated that patients with high m^6^A scores had increased resistance to immunotherapy, which may lead to different treatment effects of classical chemotherapeutics in different patients.

## 5 Conclusions

We assessed the landscape of m^6^A methylation modifications mediated by 23 regulators based on 186 ESCA samples. The variety and complexity of immune infiltration in the TME are closely connected to m^6^A methylation modifications. An m^6^A score has been developed to offer a comprehensive evaluation of the m^6^A modification pattern and immune infiltration features within a singular tumor. This score also helps determine the tumor’s immune phenotype, providing new insights and directions for identifying potential therapeutic targets.

## Data Availability

All code and data related to this study are available via GitHub: https://github.com/mintsun0035/m6aTMEESCARBMX.

## Supplementary material

Supplementary Figure 1 Integrated analysis and study design flowchart.

Supplementary Figure 2 The Metascape enrichment network is visually represented through a visualization that highlights similarities both within and between clusters of terms. The clustering of terms is indicated by consistent color coding.

Supplementary Figure 3 Kaplan-Meier survival curves of overall survival in ESCA cohort according to the expression value of YTHDF2, YTHDF1, RBMX, LRPPRC, IGFBP3, IGFBP1, FMR1 or ALKBH5 mRNA level in each tumor sample, the optimal value in each cohort was chosen as the cut-off point.

Supplementary Figure 4 Comparative immune landscape analysis of m^6^A modification clusters in esophageal cancer. Heatmap depicting immune cell infiltration patterns between m^6^A cluster A (left) and cluster B (right) as quantified by four deconvolution algorithms (CIBERSORT, EPIC, MCP_COUNTER, QUANTISEQ, TIMER and XCELL). Rows represent immune cell subsets grouped by lineage (T cells, B cells, myeloid cells, stromal cells), while columns represent individual samples. Color scale indicates relative abundance (z-score normalized).

Supplementary Figure 5 Relationship between the m^6^A score and different clinical characteristics. Kaplan-Meier curves showing the differences in survival depending on the m^6^A score and different clinical characteristics. **(A)** T0; **(B)** T1–2; **(C)** T 3–4; **(D)** N0; **(E)** N1–2; **(F)** N3; **(G)** M0; **(H)** M1; **(I)** male; **(J)** female; **(K)** age less than or equal to 65 years; **(L)** age above 65 years.

Supplementary Figure 6 The m^6^A risk score predicts immunotherapeutic benefits in the GSE165252 cohort. The fraction of patients with clinical response to anti-PD-1 immunotherapy in low or high m^6^A risk score groups.

Supplementary Figure 7 Sensitivity of the m^6^A risk score to different chemotherapy drugs and small molecule anticancer drugs was analyzed based on the GDSC database. **(A)** Axitinib, **(B)** Bexarotene, **(C)** Bicalutamide, **(D)** Vinblastine, **(E)** Bosutinib, **(F)** Bryostatin.1, **(G)** Lapatinib, **(H)** Imatinib, **(I)** Elesclomol.
