# Supplementary figures and images for: Cross-talk of m6A methylation modification and the tumor microenvironment composition in esophageal cancer
Source: Front Immunol. 2025 Jul 7;16:1572810. doi: 10.3389/fimmu.2025.1572810 (PMC12277809; doi:10.3389/fimmu.2025.1572810)

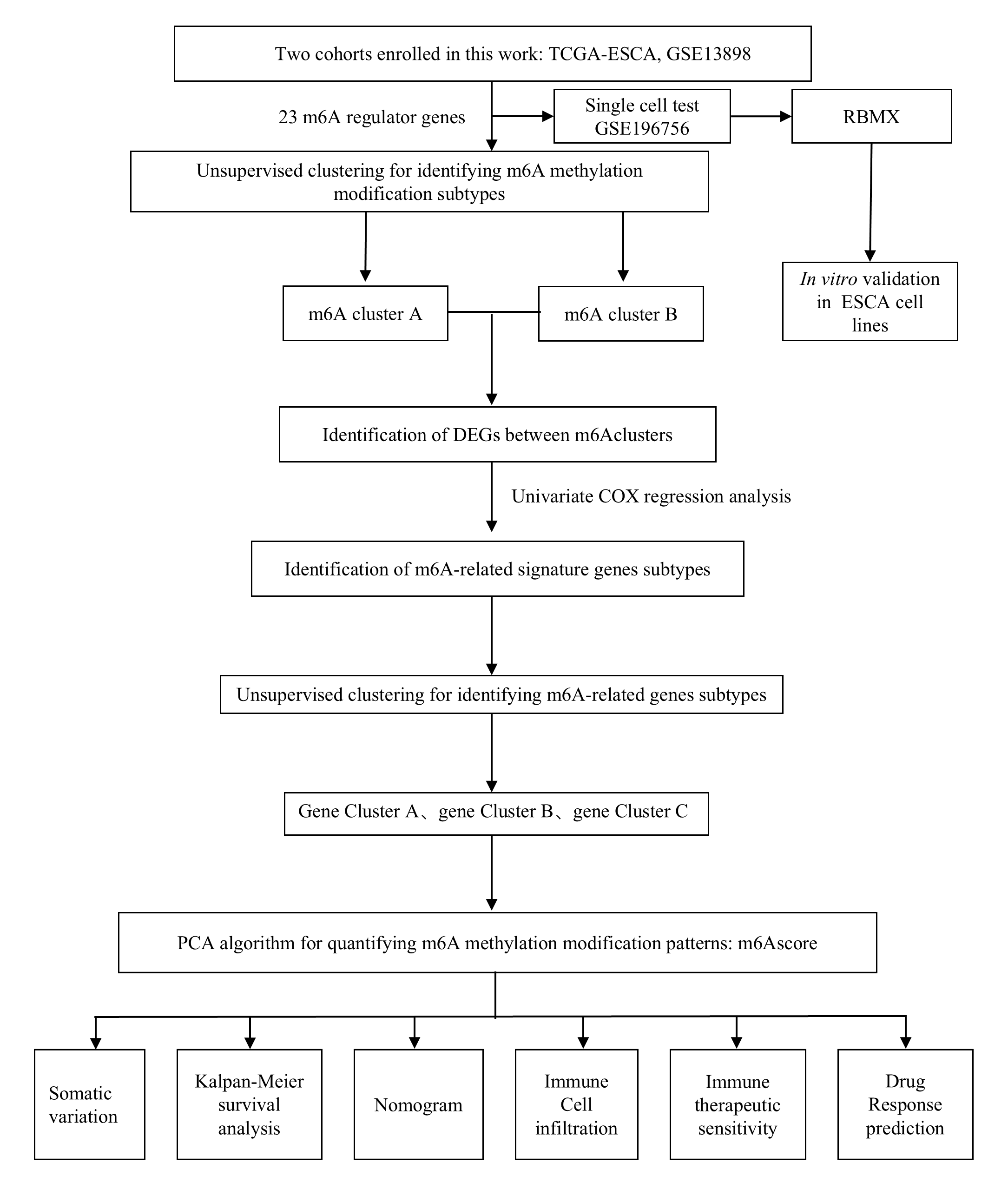

Supplement: Supplementary Figure 1 — Integrated analysis and study design flowchart. [file Image1.tif]

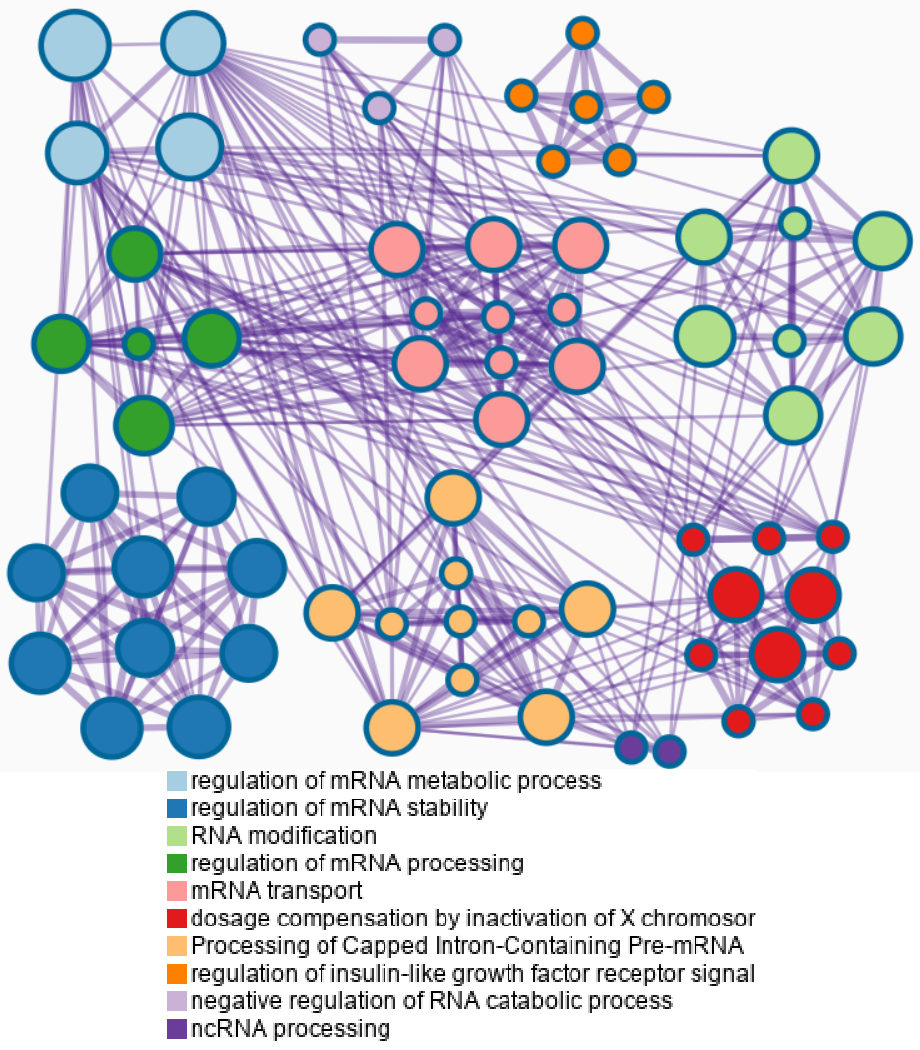

Supplement: Supplementary Figure 2 — The Metascape enrichment network is visually represented through a visualization that highlights similarities both within and between clusters of terms. The clustering of terms is indicated by consistent color coding. [file Image2.tif]

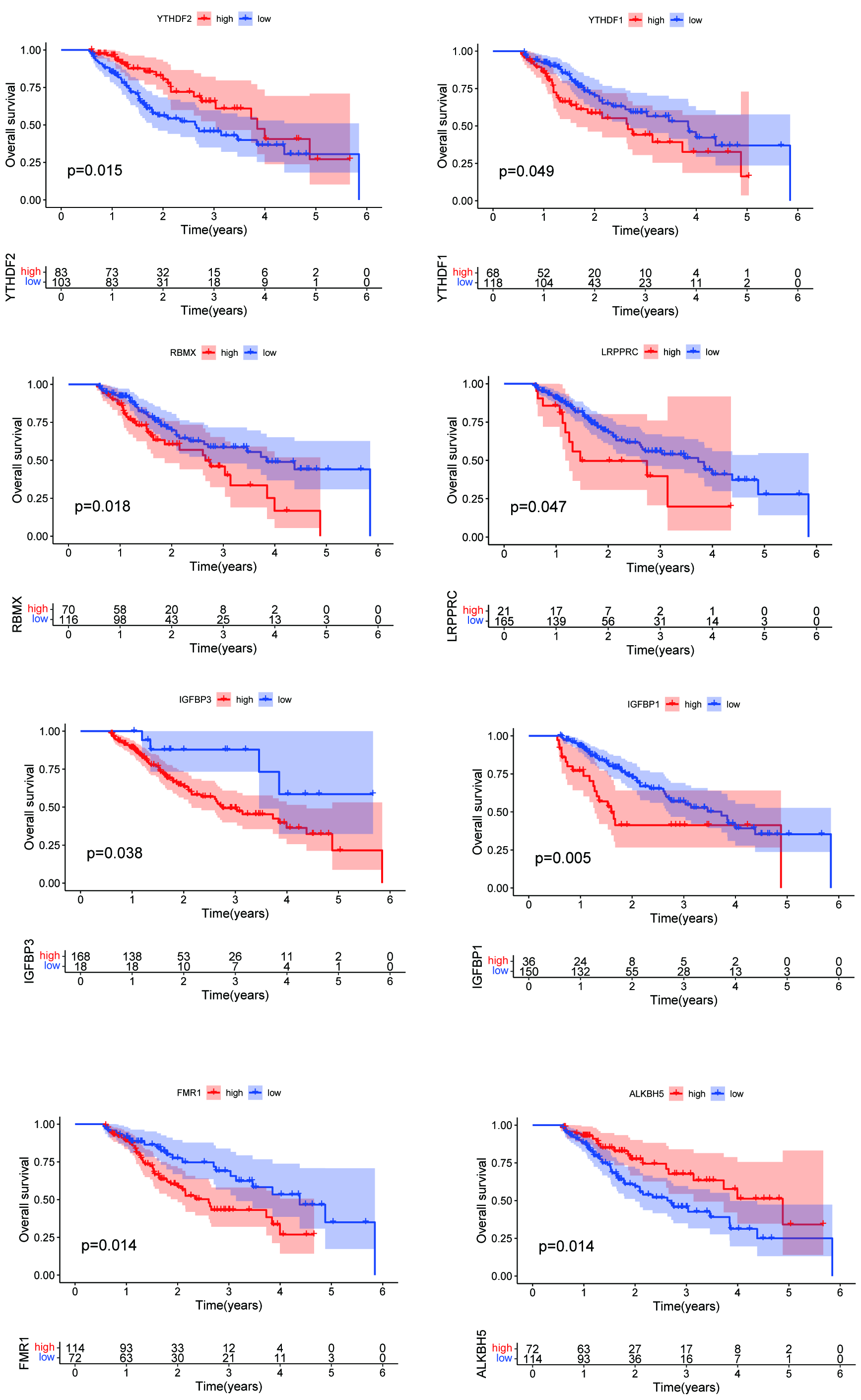

Supplement: Supplementary Figure 3 — Kaplan-Meier survival curves of overall survival in ESCA cohort according to the expression value of YTHDF2, YTHDF1, RBMX, LRPPRC, IGFBP3, IGFBP1, FMR1 or ALKBH5 mRNA level in each tumor sample, the optimal value in each cohort was chosen as the cut-off point. [file Image3.tif]

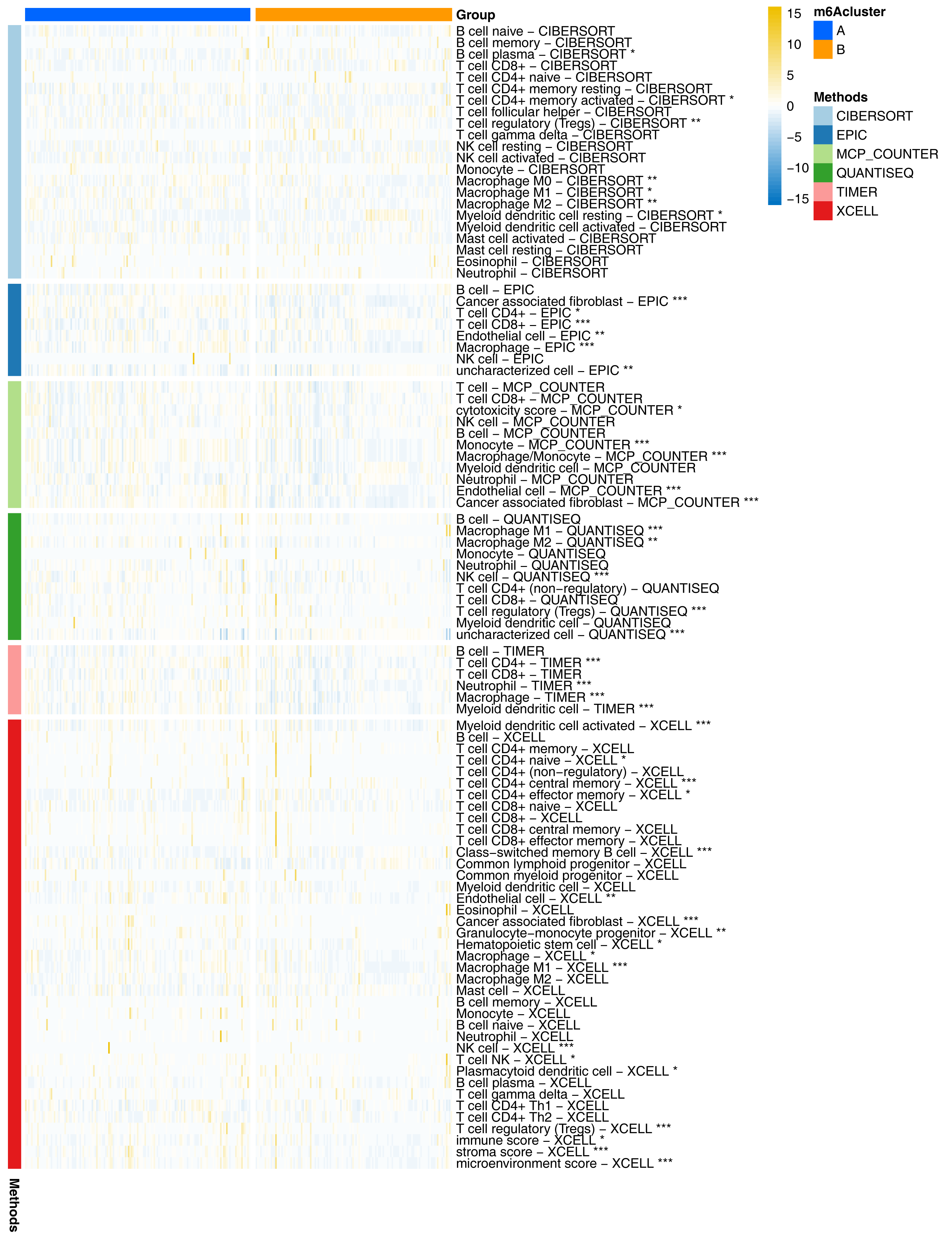

Supplement: Supplementary Figure 4 — Comparative immune landscape analysis of m6A modification clusters in esophageal cancer. Heatmap depicting immune cell infiltration patterns between m6A cluster A (left) and cluster B (right) as quantified by four deconvolution algorithms (CIBERSORT, EPIC, MCP_COUNTER, QUANTISEQ, TIMER and XCELL). Rows represent immune cell subsets grouped by lineage (T cells, B cells, myeloid cells, stromal cells), while columns represent individual samples. Color scale indicates relative abundance (z-score normalized). [file Image4.tif]

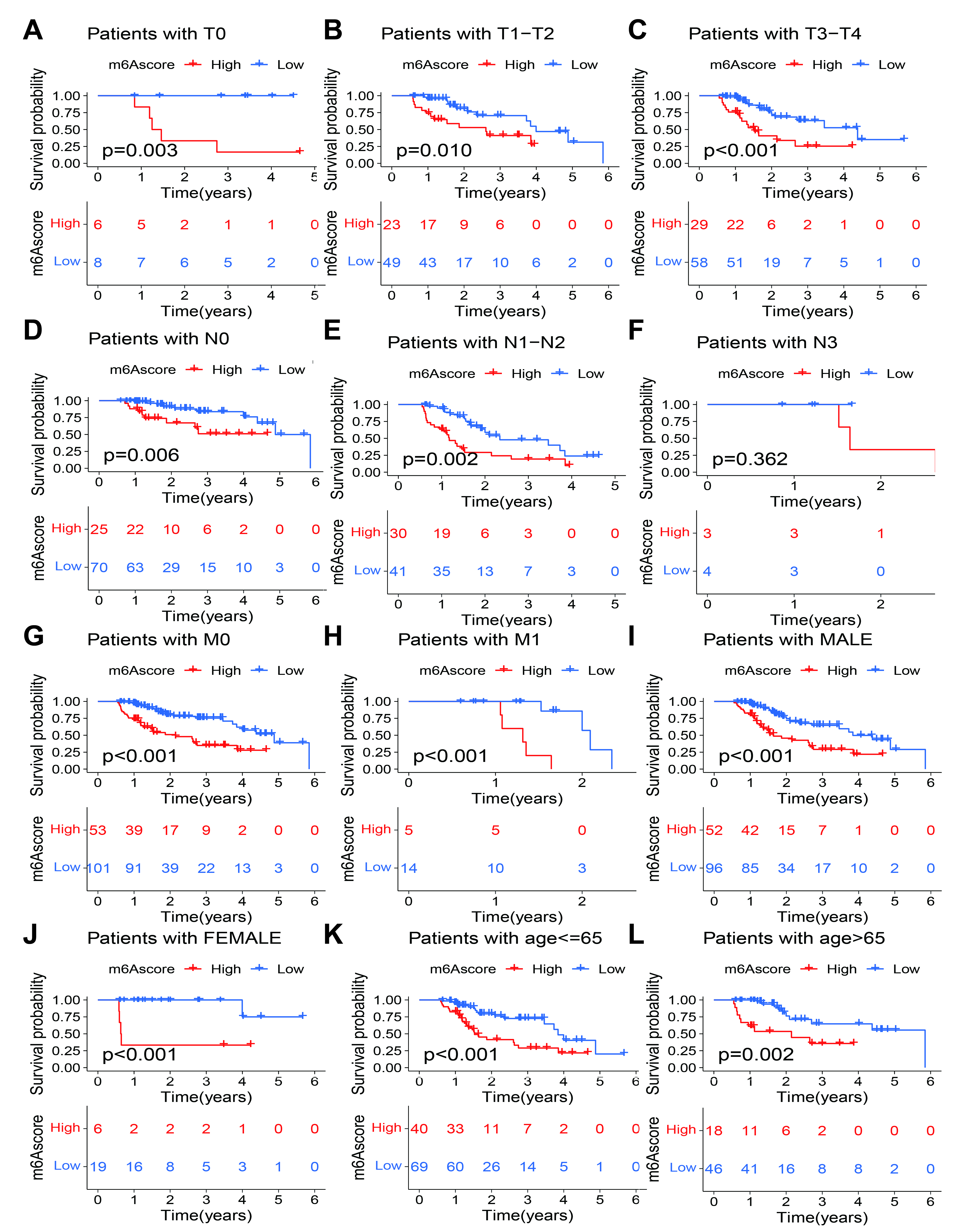

Supplement: Supplementary Figure 5 — Relationship between the m6A score and different clinical characteristics. Kaplan-Meier curves showing the differences in survival depending on the m6A score and different clinical characteristics. (A) T0; (B) T1–2; (C) T 3–4; (D) N0; (E) N1–2; (F) N3; (G) M0; (H) M1; (I) male; (J) female; (K) age less than or equal to 65 years; (L) age above 65 years. [file Image5.tif]

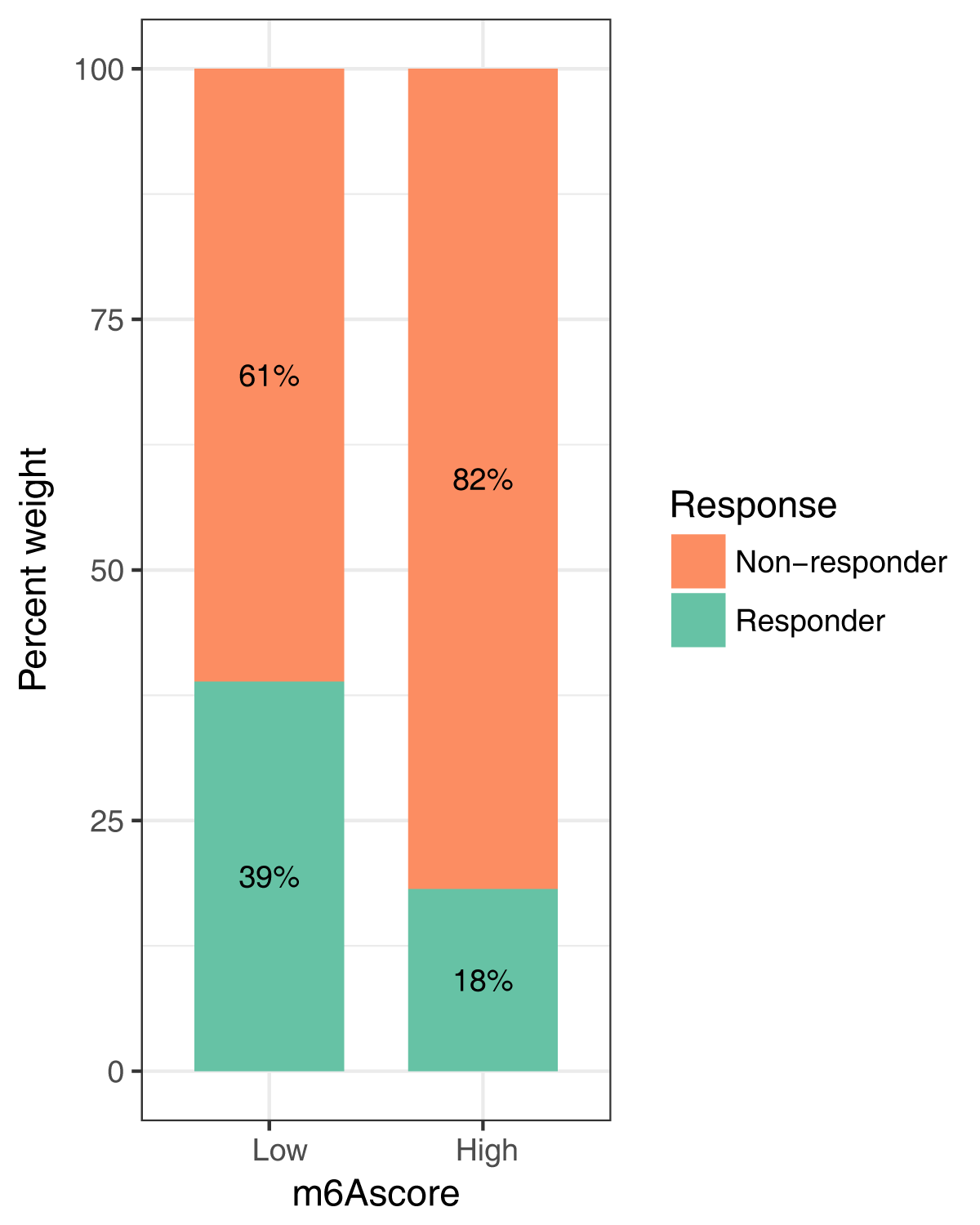

Supplement: Supplementary Figure 6 — The m6A risk score predicts immunotherapeutic benefits in the GSE165252 cohort. The fraction of patients with clinical response to anti-PD-1 immunotherapy in low or high m6A risk score groups. [file Image6.tif]

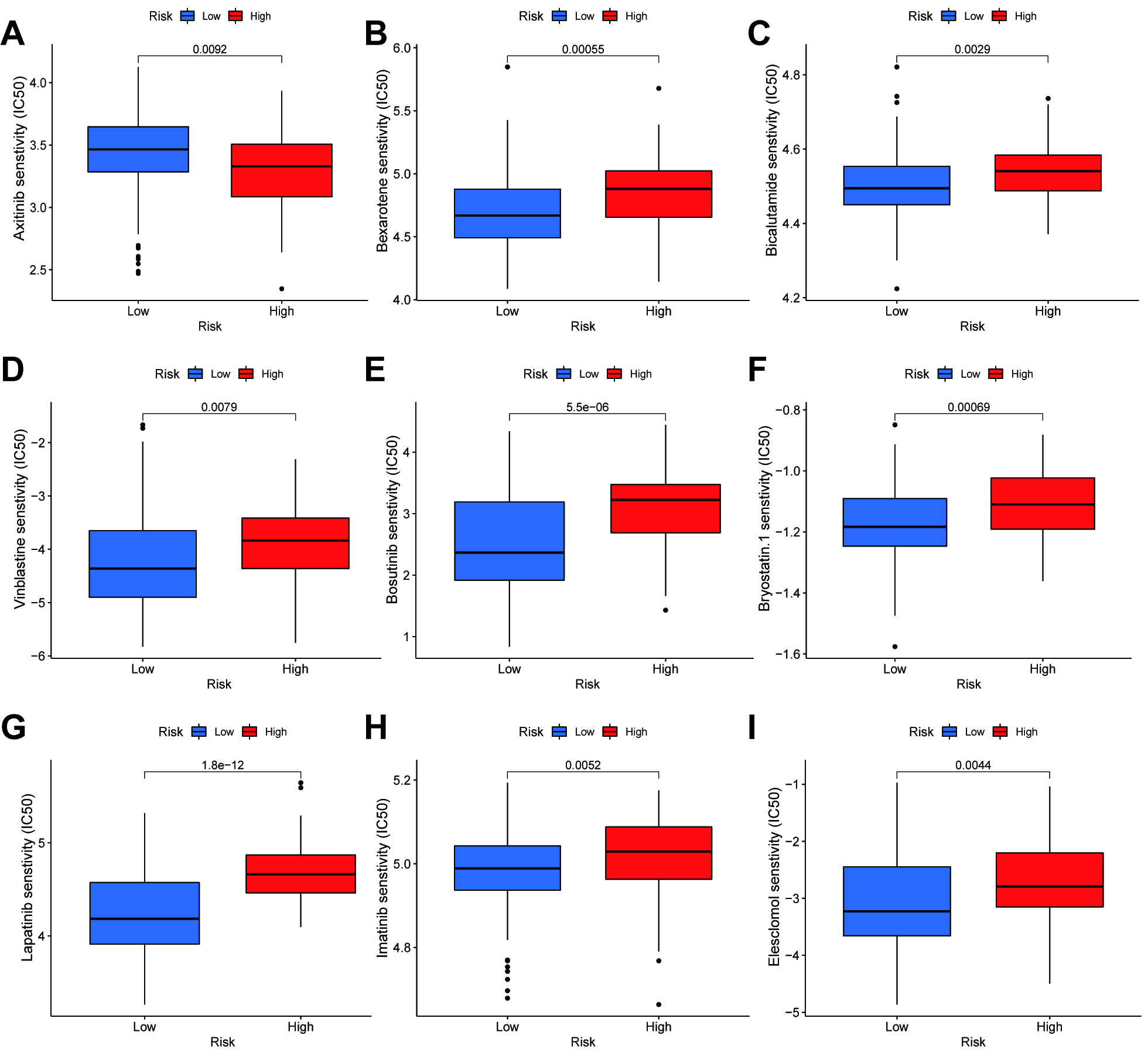

Supplement: Supplementary Figure 7 — Sensitivity of the m6A risk score to different chemotherapy drugs and small molecule anticancer drugs was analyzed based on the GDSC database. (A) Axitinib, (B) Bexarotene, (C) Bicalutamide, (D) Vinblastine, (E) Bosutinib, (F) Bryostatin.1, (G) Lapatinib, (H) Imatinib, (I) Elesclomol. [file Image7.tif]
