# Supplementary material for: Cross-talk of m6A methylation modification and the tumor microenvironment composition in esophageal cancer
Source: Front Immunol. 2025 Jul 7;16:1572810. doi: 10.3389/fimmu.2025.1572810 (PMC12277809; doi:10.3389/fimmu.2025.1572810)
Supplement: Supplementary file 8 [file Table1.docx]

**Supplementary Table S1.** Prognostic analysis of 17 m6A regulators in esophagus cancer.

| Gene symbol | HR | Lower bound of the confidence interval | Upper bound of the confidence interval | P value of Univariate Cox regression analysis | P value of Kaplan-Meier survival analysis |
| --- | --- | --- | --- | --- | --- |
| METTL3 | 1.100 | 0.679 | 1.781 | 0.697 | 0.106 |
| WTAP | 1.157 | 0.595 | 2.249 | 0.667 | 0.052 |
| ZC3H13 | 0.767 | 0.482 | 1.220 | 0.264 | 0.109 |
| RBM15 | 1.211 | 0.750 | 1.956 | 0.432 | 0.201 |
| RBM15B | 0.768 | 0.455 | 1.297 | 0.325 | 0.131 |
| YTHDC1 | 0.933 | 0.507 | 1.717 | 0.824 | 0.221 |
| YTHDC2 | 0.805 | 0.432 | 1.499 | 0.494 | 0.073 |
| YTHDF1 | 1.248 | 0.808 | 1.928 | 0.317 | 0.049 |
| YTHDF2 | 0.701 | 0.385 | 1.276 | 0.245 | 0.015 |
| YTHDF3 | 0.912 | 0.572 | 1.455 | 0.701 | 0.088 |
| FMR1 | 1.290 | 0.835 | 1.994 | 0.250 | 0.013 |
| LRPPRC | 1.399 | 0.874 | 2.238 | 0.160 | 0.047 |
| IGFBP1 | 1.210 | 0.948 | 1.544 | 0.124 | 0.005 |
| IGFBP2 | 1.021 | 0.874 | 1.192 | 0.791 | 0.084 |
| IGFBP3 | 1.080 | 0.920 | 1.267 | 0.343 | 0.037 |
| RBMX | 1.348 | 0.779 | 2.332 | 0.284 | 0.017 |
| ALKBH5 | 0.629 | 0.401 | 0.986 | 0.043 | 0.013 |

HR: hazard ratio.
