# Supplementary material for: Cross-talk of m6A methylation modification and the tumor microenvironment composition in esophageal cancer
Source: Front Immunol. 2025 Jul 7;16:1572810. doi: 10.3389/fimmu.2025.1572810 (PMC12277809; doi:10.3389/fimmu.2025.1572810)
Supplement: Supplementary file 9 [file Table2.docx]

Supplementary Table S2**.** 23 regulators-mediated m^6^A methylation modification subtypes

| ID | m6Acluster |
| --- | --- |
| TCGA_TCGA-LN-A7HZ | A |
| TCGA_TCGA-V5-A7RC | A |
| TCGA_TCGA-R6-A6L4 | B |
| TCGA_TCGA-LN-A49O | A |
| TCGA_TCGA-R6-A8W5 | B |
| TCGA_TCGA-LN-A9FQ | A |
| TCGA_TCGA-LN-A49P | A |
| TCGA_TCGA-LN-A9FR | A |
| TCGA_TCGA-LN-A4A8 | B |
| TCGA_TCGA-IG-A4QS | B |
| TCGA_TCGA-L5-A8NM | B |
| TCGA_TCGA-LN-A7HV | B |
| TCGA_TCGA-VR-A8EZ | A |
| TCGA_TCGA-IG-A3YC | B |
| TCGA_TCGA-Z6-AAPN | A |
| TCGA_TCGA-LN-A49W | A |
| TCGA_TCGA-L5-A8NE | B |
| TCGA_TCGA-Z6-A9VB | A |
| TCGA_TCGA-L5-A8NV | B |
| TCGA_TCGA-R6-A6Y0 | B |
| TCGA_TCGA-IG-A625 | A |
| TCGA_TCGA-R6-A6XQ | B |
| TCGA_TCGA-2H-A9GG | A |
| TCGA_TCGA-2H-A9GN | A |
| TCGA_TCGA-VR-A8EX | B |
| TCGA_TCGA-IG-A5S3 | A |
| TCGA_TCGA-Z6-A8JD | A |
| TCGA_TCGA-2H-A9GQ | A |
| TCGA_TCGA-LN-A9FO | A |
| TCGA_TCGA-L5-A8NJ | B |
| TCGA_TCGA-IG-A7DP | B |
| TCGA_TCGA-VR-A8EQ | B |
| TCGA_TCGA-LN-A49S | B |
| TCGA_TCGA-LN-A8HZ | A |
| TCGA_TCGA-VR-A8EW | A |
| TCGA_TCGA-LN-A49X | B |
| TCGA_TCGA-R6-A6DN | A |
| TCGA_TCGA-IG-A5B8 | B |
| TCGA_TCGA-LN-A7HW | A |
| TCGA_TCGA-L5-A8NH | B |
| TCGA_TCGA-L5-A88T | B |
| TCGA_TCGA-VR-A8EO | B |
| TCGA_TCGA-L5-A4OE | B |
| TCGA_TCGA-JY-A6FA | A |
| TCGA_TCGA-2H-A9GJ | B |
| TCGA_TCGA-L5-A4OU | B |
| TCGA_TCGA-Z6-A8JE | B |
| TCGA_TCGA-Q9-A6FW | A |
| TCGA_TCGA-R6-A8WC | A |
| TCGA_TCGA-V5-AASW | B |
| TCGA_TCGA-RE-A7BO | A |
| TCGA_TCGA-ZR-A9CJ | A |
| TCGA_TCGA-L5-A4OT | A |
| TCGA_TCGA-IG-A6QS | A |
| TCGA_TCGA-LN-A5U6 | A |
| TCGA_TCGA-XP-A8T6 | A |
| TCGA_TCGA-KH-A6WC | B |
| TCGA_TCGA-L5-A893 | B |
| TCGA_TCGA-JY-A6FG | B |
| TCGA_TCGA-VR-AA4D | B |
| TCGA_TCGA-S8-A6BW | A |
| TCGA_TCGA-IG-A3YA | B |
| TCGA_TCGA-LN-A4MQ | A |
| TCGA_TCGA-L5-A4OP | B |
| TCGA_TCGA-L5-A891 | A |
| TCGA_TCGA-L5-A4OM | A |
| TCGA_TCGA-LN-A7HX | B |
| TCGA_TCGA-2H-A9GL | A |
| TCGA_TCGA-LN-A4A4 | A |
| TCGA_TCGA-JY-A6FE | B |
| TCGA_TCGA-JY-A6FH | A |
| TCGA_TCGA-R6-A8WG | A |
| TCGA_TCGA-L5-A8NT | A |
| TCGA_TCGA-L5-A4OH | A |
| TCGA_TCGA-L5-A8NI | A |
| TCGA_TCGA-L5-A8NQ | B |
| TCGA_TCGA-VR-A8ET | B |
| TCGA_TCGA-L5-A43J | A |
| TCGA_TCGA-2H-A9GR | A |
| TCGA_TCGA-L5-A8NU | B |
| TCGA_TCGA-L5-A4OX | A |
| TCGA_TCGA-L5-A8NS | A |
| TCGA_TCGA-L7-A6VZ | B |
| TCGA_TCGA-LN-A49Y | B |
| TCGA_TCGA-VR-AA7I | A |
| TCGA_TCGA-LN-A5U7 | A |
| TCGA_TCGA-JY-A93D | A |
| TCGA_TCGA-R6-A6XG | B |
| TCGA_TCGA-L5-A4OW | A |
| TCGA_TCGA-JY-A6FB | B |
| TCGA_TCGA-JY-A938 | A |
| TCGA_TCGA-2H-A9GO | B |
| TCGA_TCGA-L5-A4OI | A |
| TCGA_TCGA-2H-A9GI | B |
| TCGA_TCGA-L7-A56G | A |
| TCGA_TCGA-XP-A8T8 | A |
| TCGA_TCGA-LN-A4A1 | A |
| TCGA_TCGA-JY-A93F | B |
| TCGA_TCGA-LN-A7HY | A |
| TCGA_TCGA-LN-A4A9 | A |
| TCGA_TCGA-L5-A8NN | B |
| TCGA_TCGA-2H-A9GK | A |
| TCGA_TCGA-JY-A93E | A |
| TCGA_TCGA-L5-A8NF | B |
| TCGA_TCGA-IG-A97I | A |
| TCGA_TCGA-L5-A8NR | A |
| TCGA_TCGA-VR-AA4G | A |
| TCGA_TCGA-VR-A8Q7 | B |
| TCGA_TCGA-LN-A8I0 | B |
| TCGA_TCGA-VR-A8EU | A |
| TCGA_TCGA-LN-A4A3 | B |
| TCGA_TCGA-VR-A8EP | B |
| TCGA_TCGA-L5-A4OJ | B |
| TCGA_TCGA-L5-A4OG | B |
| TCGA_TCGA-V5-AASX | B |
| TCGA_TCGA-S8-A6BV | A |
| TCGA_TCGA-L5-A4ON | A |
| TCGA_TCGA-IC-A6RF | B |
| TCGA_TCGA-IG-A3QL | A |
| TCGA_TCGA-VR-A8ER | A |
| TCGA_TCGA-L5-A8NL | A |
| TCGA_TCGA-LN-A8I1 | A |
| TCGA_TCGA-LN-A9FP | A |
| TCGA_TCGA-L5-A43C | A |
| TCGA_TCGA-V5-A7RE | B |
| TCGA_TCGA-L5-A88S | A |
| TCGA_TCGA-LN-A4A5 | A |
| TCGA_TCGA-IG-A3I8 | A |
| TCGA_TCGA-V5-A7RB | B |
| TCGA_TCGA-L5-A8NG | A |
| TCGA_TCGA-M9-A5M8 | B |
| TCGA_TCGA-L5-A4OS | A |
| TCGA_TCGA-IC-A6RE | B |
| TCGA_TCGA-L5-A88V | B |
| TCGA_TCGA-LN-A49M | B |
| TCGA_TCGA-VR-A8EY | B |
| TCGA_TCGA-JY-A93C | A |
| TCGA_TCGA-L5-A88W | B |
| TCGA_TCGA-R6-A6KZ | A |
| TCGA_TCGA-2H-A9GF | A |
| TCGA_TCGA-JY-A6FD | B |
| TCGA_TCGA-IG-A8O2 | A |
| TCGA_TCGA-IG-A4P3 | A |
| TCGA_TCGA-LN-A49U | B |
| TCGA_TCGA-2H-A9GH | A |
| TCGA_TCGA-LN-A5U5 | A |
| TCGA_TCGA-IG-A50L | A |
| TCGA_TCGA-IG-A3YB | A |
| TCGA_TCGA-JY-A939 | B |
| TCGA_TCGA-L5-A43E | B |
| TCGA_TCGA-IG-A97H | A |
| TCGA_TCGA-2H-A9GM | B |
| TCGA_TCGA-L5-A4OO | A |
| TCGA_TCGA-L5-A8NW | B |
| TCGA_TCGA-L5-A88Y | B |
| TCGA_TCGA-R6-A6DQ | B |
| TCGA_TCGA-L5-A88Z | B |
| TCGA_TCGA-IG-A51D | B |
| TCGA_TCGA-R6-A8W8 | A |
| TCGA_TCGA-L5-A8NK | B |
| TCGA_TCGA-V5-AASV | A |
| GSE13898_GSM349960 | B |
| GSE13898_GSM349961 | B |
| GSE13898_GSM349962 | B |
| GSE13898_GSM349963 | B |
| GSE13898_GSM349964 | B |
| GSE13898_GSM349965 | B |
| GSE13898_GSM349966 | B |
| GSE13898_GSM349967 | B |
| GSE13898_GSM349968 | B |
| GSE13898_GSM349969 | B |
| GSE13898_GSM349970 | B |
| GSE13898_GSM349971 | B |
| GSE13898_GSM349972 | B |
| GSE13898_GSM349973 | B |
| GSE13898_GSM349974 | B |
| GSE13898_GSM349975 | B |
| GSE13898_GSM349976 | A |
| GSE13898_GSM349977 | B |
| GSE13898_GSM349978 | B |
| GSE13898_GSM349979 | B |
| GSE13898_GSM349980 | B |
| GSE13898_GSM349981 | B |
| GSE13898_GSM349982 | B |
| GSE13898_GSM349983 | B |
| GSE13898_GSM349984 | B |
| GSE13898_GSM349985 | B |
| GSE13898_GSM349986 | B |
| GSE13898_GSM349987 | B |
| GSE13898_GSM349988 | A |
| GSE13898_GSM349989 | B |
| GSE13898_GSM349990 | B |
| GSE13898_GSM349991 | B |
| GSE13898_GSM349992 | B |
| GSE13898_GSM349993 | B |
| GSE13898_GSM349994 | A |
| GSE13898_GSM349995 | A |
| GSE13898_GSM349996 | A |
| GSE13898_GSM349997 | B |
| GSE13898_GSM349998 | A |
| GSE13898_GSM349999 | B |
| GSE13898_GSM350000 | A |
| GSE13898_GSM350001 | B |
| GSE13898_GSM350002 | B |
| GSE13898_GSM350003 | A |
| GSE13898_GSM350004 | A |
| GSE13898_GSM350005 | A |
| GSE13898_GSM350006 | A |
| GSE13898_GSM350007 | A |
| GSE13898_GSM350008 | B |
| GSE13898_GSM350009 | A |
| GSE13898_GSM350010 | A |
| GSE13898_GSM350011 | A |
| GSE13898_GSM350012 | A |
| GSE13898_GSM350013 | A |
| GSE13898_GSM350014 | B |
| GSE13898_GSM350015 | B |
| GSE13898_GSM350016 | A |
| GSE13898_GSM350017 | A |
| GSE13898_GSM350018 | A |
| GSE13898_GSM350019 | B |
| GSE13898_GSM350020 | B |
| GSE13898_GSM350021 | A |
| GSE13898_GSM350022 | A |
| GSE13898_GSM350023 | B |
| GSE13898_GSM350024 | A |
| GSE13898_GSM350025 | A |
| GSE13898_GSM350026 | B |
| GSE13898_GSM350027 | A |
| GSE13898_GSM350028 | B |
| GSE13898_GSM350029 | B |
| GSE13898_GSM350030 | B |
| GSE13898_GSM350031 | A |
| GSE13898_GSM350032 | A |
| GSE13898_GSM350033 | A |
| GSE13898_GSM350034 | A |
| GSE13898_GSM350035 | A |
| GSE13898_GSM350036 | A |
| GSE13898_GSM350037 | A |
| GSE13898_GSM350038 | A |
| GSE13898_GSM350039 | A |
| GSE13898_GSM350040 | A |
| GSE13898_GSM350041 | A |
| GSE13898_GSM350042 | A |
| GSE13898_GSM350043 | A |
| GSE13898_GSM350044 | A |
| GSE13898_GSM350045 | A |
| GSE13898_GSM350046 | A |
| GSE13898_GSM350047 | A |
| GSE13898_GSM350048 | A |
| GSE13898_GSM350049 | A |
| GSE13898_GSM350050 | A |
| GSE13898_GSM350051 | A |
| GSE13898_GSM350052 | A |
| GSE13898_GSM350053 | A |
| GSE13898_GSM350054 | A |
| GSE13898_GSM350055 | A |
| GSE13898_GSM350056 | B |
| GSE13898_GSM350057 | B |
| GSE13898_GSM350058 | A |
| GSE13898_GSM350059 | A |
| GSE13898_GSM350060 | A |
| GSE13898_GSM350061 | B |
| GSE13898_GSM350062 | B |
| GSE13898_GSM350063 | B |
| GSE13898_GSM350064 | A |
| GSE13898_GSM350065 | B |
| GSE13898_GSM350066 | B |
| GSE13898_GSM350067 | A |
| GSE13898_GSM350068 | B |
| GSE13898_GSM350069 | B |
| GSE13898_GSM350070 | A |
| GSE13898_GSM350071 | A |
| GSE13898_GSM350072 | A |
| GSE13898_GSM350073 | A |
| GSE13898_GSM350074 | A |
| GSE13898_GSM350075 | B |
| GSE13898_GSM350076 | A |
| GSE13898_GSM350077 | B |
