# Supplementary material for: Cross-talk of m6A methylation modification and the tumor microenvironment composition in esophageal cancer
Source: Front Immunol. 2025 Jul 7;16:1572810. doi: 10.3389/fimmu.2025.1572810 (PMC12277809; doi:10.3389/fimmu.2025.1572810)
Supplement: Supplementary file 10 [file Table3.docx]

**Supplementary Table S3.** GO analysis of differentially expressed genes between two m^6^A modified subtypes.

| ONTOLOGY | ID | Description | pvalue | p.adjust | qvalue |
| --- | --- | --- | --- | --- | --- |
| BP | GO:0045229 | external encapsulating structure organization | 1.47E-47 | 9.00E-44 | 6.58E-44 |
| BP | GO:0030198 | extracellular matrix organization | 1.12E-46 | 3.44E-43 | 2.52E-43 |
| BP | GO:0043062 | extracellular structure organization | 1.69E-46 | 3.45E-43 | 2.52E-43 |
| BP | GO:0031589 | cell-substrate adhesion | 2.40E-21 | 3.68E-18 | 2.69E-18 |
| BP | GO:0060485 | mesenchyme development | 2.40E-15 | 2.95E-12 | 2.16E-12 |
| BP | GO:0001503 | ossification | 5.02E-15 | 5.14E-12 | 3.76E-12 |
| BP | GO:0003158 | endothelium development | 6.50E-15 | 5.70E-12 | 4.17E-12 |
| BP | GO:0001667 | ameboidal-type cell migration | 9.27E-15 | 7.12E-12 | 5.20E-12 |
| BP | GO:0001501 | skeletal system development | 1.35E-14 | 9.20E-12 | 6.72E-12 |
| BP | GO:0045446 | endothelial cell differentiation | 7.27E-14 | 4.47E-11 | 3.27E-11 |
| BP | GO:0090130 | tissue migration | 9.64E-14 | 5.38E-11 | 3.94E-11 |
| BP | GO:0030199 | collagen fibril organization | 1.23E-13 | 6.30E-11 | 4.61E-11 |
| BP | GO:0007265 | Ras protein signal transduction | 2.04E-13 | 9.63E-11 | 7.04E-11 |
| BP | GO:0010631 | epithelial cell migration | 3.92E-13 | 1.72E-10 | 1.26E-10 |
| BP | GO:0090132 | epithelium migration | 6.75E-13 | 2.76E-10 | 2.02E-10 |
| BP | GO:0060562 | epithelial tube morphogenesis | 7.65E-13 | 2.94E-10 | 2.15E-10 |
| BP | GO:0045785 | positive regulation of cell adhesion | 1.98E-12 | 7.17E-10 | 5.24E-10 |
| BP | GO:0048762 | mesenchymal cell differentiation | 3.20E-12 | 1.09E-09 | 7.97E-10 |
| BP | GO:0010810 | regulation of cell-substrate adhesion | 4.55E-12 | 1.47E-09 | 1.08E-09 |
| BP | GO:0007409 | axonogenesis | 6.40E-12 | 1.97E-09 | 1.44E-09 |
| BP | GO:0003170 | heart valve development | 8.10E-12 | 2.37E-09 | 1.73E-09 |
| BP | GO:0007160 | cell-matrix adhesion | 2.25E-11 | 6.27E-09 | 4.59E-09 |
| BP | GO:0003179 | heart valve morphogenesis | 3.13E-11 | 8.36E-09 | 6.11E-09 |
| BP | GO:0072001 | renal system development | 3.58E-11 | 9.15E-09 | 6.69E-09 |
| BP | GO:0007162 | negative regulation of cell adhesion | 4.99E-11 | 1.23E-08 | 8.97E-09 |
| BP | GO:0001822 | kidney development | 5.54E-11 | 1.31E-08 | 9.56E-09 |
| BP | GO:0043393 | regulation of protein binding | 7.06E-11 | 1.61E-08 | 1.17E-08 |
| BP | GO:0061138 | morphogenesis of a branching epithelium | 8.77E-11 | 1.92E-08 | 1.41E-08 |
| BP | GO:0001655 | urogenital system development | 1.00E-10 | 2.12E-08 | 1.55E-08 |
| BP | GO:0034329 | cell junction assembly | 1.27E-10 | 2.61E-08 | 1.91E-08 |
| BP | GO:0048771 | tissue remodeling | 1.43E-10 | 2.84E-08 | 2.08E-08 |
| BP | GO:0022604 | regulation of cell morphogenesis | 2.91E-10 | 5.58E-08 | 4.08E-08 |
| BP | GO:0048568 | embryonic organ development | 4.80E-10 | 8.94E-08 | 6.53E-08 |
| BP | GO:0001763 | morphogenesis of a branching structure | 6.05E-10 | 1.09E-07 | 7.99E-08 |
| BP | GO:0002283 | neutrophil activation involved in immune response | 6.49E-10 | 1.12E-07 | 8.21E-08 |
| BP | GO:0043542 | endothelial cell migration | 6.58E-10 | 1.12E-07 | 8.21E-08 |
| BP | GO:0034446 | substrate adhesion-dependent cell spreading | 7.73E-10 | 1.28E-07 | 9.39E-08 |
| BP | GO:0043312 | neutrophil degranulation | 9.20E-10 | 1.49E-07 | 1.09E-07 |
| BP | GO:0048705 | skeletal system morphogenesis | 9.64E-10 | 1.52E-07 | 1.11E-07 |
| BP | GO:0003007 | heart morphogenesis | 1.07E-09 | 1.64E-07 | 1.20E-07 |
| BP | GO:0002446 | neutrophil mediated immunity | 1.25E-09 | 1.87E-07 | 1.37E-07 |
| BP | GO:0033627 | cell adhesion mediated by integrin | 1.34E-09 | 1.96E-07 | 1.43E-07 |
| BP | GO:0042119 | neutrophil activation | 1.41E-09 | 2.02E-07 | 1.47E-07 |
| BP | GO:0022407 | regulation of cell-cell adhesion | 1.44E-09 | 2.02E-07 | 1.47E-07 |
| BP | GO:0007229 | integrin-mediated signaling pathway | 1.61E-09 | 2.20E-07 | 1.61E-07 |
| BP | GO:0048754 | branching morphogenesis of an epithelial tube | 1.95E-09 | 2.58E-07 | 1.89E-07 |
| BP | GO:0050673 | epithelial cell proliferation | 1.98E-09 | 2.58E-07 | 1.89E-07 |
| BP | GO:0051098 | regulation of binding | 2.62E-09 | 3.25E-07 | 2.38E-07 |
| BP | GO:0008361 | regulation of cell size | 2.62E-09 | 3.25E-07 | 2.38E-07 |
| BP | GO:0045765 | regulation of angiogenesis | 2.65E-09 | 3.25E-07 | 2.38E-07 |
| BP | GO:0001885 | endothelial cell development | 2.89E-09 | 3.48E-07 | 2.54E-07 |
| BP | GO:0010632 | regulation of epithelial cell migration | 3.36E-09 | 3.96E-07 | 2.90E-07 |
| BP | GO:0032092 | positive regulation of protein binding | 3.91E-09 | 4.53E-07 | 3.31E-07 |
| BP | GO:1905314 | semi-lunar valve development | 4.32E-09 | 4.85E-07 | 3.55E-07 |
| BP | GO:0008360 | regulation of cell shape | 4.34E-09 | 4.85E-07 | 3.55E-07 |
| BP | GO:1901342 | regulation of vasculature development | 6.42E-09 | 7.04E-07 | 5.15E-07 |
| BP | GO:0001837 | epithelial to mesenchymal transition | 6.96E-09 | 7.50E-07 | 5.48E-07 |
| BP | GO:0003197 | endocardial cushion development | 7.58E-09 | 8.03E-07 | 5.87E-07 |
| BP | GO:0003181 | atrioventricular valve morphogenesis | 8.37E-09 | 8.71E-07 | 6.37E-07 |
| BP | GO:0045216 | cell-cell junction organization | 8.85E-09 | 9.06E-07 | 6.62E-07 |
| BP | GO:0032102 | negative regulation of response to external stimulus | 9.00E-09 | 9.06E-07 | 6.63E-07 |
| BP | GO:0007159 | leukocyte cell-cell adhesion | 9.34E-09 | 9.25E-07 | 6.76E-07 |
| BP | GO:0097485 | neuron projection guidance | 9.66E-09 | 9.31E-07 | 6.81E-07 |
| BP | GO:0060560 | developmental growth involved in morphogenesis | 9.70E-09 | 9.31E-07 | 6.81E-07 |
| BP | GO:0002064 | epithelial cell development | 1.61E-08 | 1.52E-06 | 1.11E-06 |
| BP | GO:0071900 | regulation of protein serine/threonine kinase activity | 1.73E-08 | 1.61E-06 | 1.18E-06 |
| BP | GO:0072006 | nephron development | 1.88E-08 | 1.73E-06 | 1.26E-06 |
| BP | GO:0007411 | axon guidance | 1.95E-08 | 1.76E-06 | 1.29E-06 |
| BP | GO:0050920 | regulation of chemotaxis | 2.07E-08 | 1.82E-06 | 1.33E-06 |
| BP | GO:0060348 | bone development | 2.10E-08 | 1.82E-06 | 1.33E-06 |
| BP | GO:0018212 | peptidyl-tyrosine modification | 2.10E-08 | 1.82E-06 | 1.33E-06 |
| BP | GO:0007266 | Rho protein signal transduction | 2.27E-08 | 1.93E-06 | 1.41E-06 |
| BP | GO:0060759 | regulation of response to cytokine stimulus | 2.60E-08 | 2.19E-06 | 1.60E-06 |
| BP | GO:0002831 | regulation of response to biotic stimulus | 2.75E-08 | 2.29E-06 | 1.67E-06 |
| BP | GO:0002683 | negative regulation of immune system process | 2.87E-08 | 2.35E-06 | 1.72E-06 |
| BP | GO:0018108 | peptidyl-tyrosine phosphorylation | 2.99E-08 | 2.42E-06 | 1.77E-06 |
| BP | GO:0021915 | neural tube development | 3.13E-08 | 2.49E-06 | 1.82E-06 |
| BP | GO:0090066 | regulation of anatomical structure size | 3.77E-08 | 2.97E-06 | 2.17E-06 |
| BP | GO:0040013 | negative regulation of locomotion | 4.03E-08 | 3.13E-06 | 2.29E-06 |
| BP | GO:0072132 | mesenchyme morphogenesis | 4.09E-08 | 3.14E-06 | 2.30E-06 |
| BP | GO:0003171 | atrioventricular valve development | 4.32E-08 | 3.27E-06 | 2.39E-06 |
| BP | GO:0001649 | osteoblast differentiation | 4.38E-08 | 3.28E-06 | 2.40E-06 |
| BP | GO:0048675 | axon extension | 5.55E-08 | 4.11E-06 | 3.00E-06 |
| BP | GO:0048844 | artery morphogenesis | 6.26E-08 | 4.58E-06 | 3.35E-06 |
| BP | GO:0051271 | negative regulation of cellular component movement | 6.37E-08 | 4.60E-06 | 3.37E-06 |
| BP | GO:0010975 | regulation of neuron projection development | 7.19E-08 | 5.13E-06 | 3.75E-06 |
| BP | GO:0002685 | regulation of leukocyte migration | 7.48E-08 | 5.28E-06 | 3.86E-06 |
| BP | GO:0030111 | regulation of Wnt signaling pathway | 7.82E-08 | 5.46E-06 | 3.99E-06 |
| BP | GO:0050727 | regulation of inflammatory response | 8.88E-08 | 6.07E-06 | 4.44E-06 |
| BP | GO:0022409 | positive regulation of cell-cell adhesion | 8.92E-08 | 6.07E-06 | 4.44E-06 |
| BP | GO:0003176 | aortic valve development | 9.00E-08 | 6.07E-06 | 4.44E-06 |
| BP | GO:1903039 | positive regulation of leukocyte cell-cell adhesion | 1.14E-07 | 7.55E-06 | 5.52E-06 |
| BP | GO:0097529 | myeloid leukocyte migration | 1.14E-07 | 7.55E-06 | 5.52E-06 |
| BP | GO:0050678 | regulation of epithelial cell proliferation | 1.18E-07 | 7.69E-06 | 5.62E-06 |
| BP | GO:0001959 | regulation of cytokine-mediated signaling pathway | 1.33E-07 | 8.61E-06 | 6.30E-06 |
| BP | GO:0007178 | transmembrane receptor protein serine/threonine kinase signaling pathway | 1.38E-07 | 8.83E-06 | 6.45E-06 |
| BP | GO:0030336 | negative regulation of cell migration | 1.48E-07 | 9.30E-06 | 6.80E-06 |
| BP | GO:1903037 | regulation of leukocyte cell-cell adhesion | 1.48E-07 | 9.30E-06 | 6.80E-06 |
| BP | GO:0060840 | artery development | 1.64E-07 | 1.02E-05 | 7.46E-06 |
| BP | GO:0032963 | collagen metabolic process | 1.69E-07 | 1.04E-05 | 7.60E-06 |
| BP | GO:0006023 | aminoglycan biosynthetic process | 1.81E-07 | 1.10E-05 | 8.07E-06 |
| BP | GO:0061298 | retina vasculature development in camera-type eye | 1.93E-07 | 1.16E-05 | 8.45E-06 |
| BP | GO:0045088 | regulation of innate immune response | 1.94E-07 | 1.16E-05 | 8.45E-06 |
| BP | GO:1990138 | neuron projection extension | 2.10E-07 | 1.24E-05 | 9.06E-06 |
| BP | GO:0050921 | positive regulation of chemotaxis | 2.16E-07 | 1.25E-05 | 9.16E-06 |
| BP | GO:0003279 | cardiac septum development | 2.16E-07 | 1.25E-05 | 9.16E-06 |
| BP | GO:2000146 | negative regulation of cell motility | 2.35E-07 | 1.35E-05 | 9.88E-06 |
| BP | GO:0030178 | negative regulation of Wnt signaling pathway | 2.50E-07 | 1.42E-05 | 1.04E-05 |
| BP | GO:0060317 | cardiac epithelial to mesenchymal transition | 2.68E-07 | 1.51E-05 | 1.10E-05 |
| BP | GO:0032535 | regulation of cellular component size | 2.97E-07 | 1.66E-05 | 1.21E-05 |
| BP | GO:0043405 | regulation of MAP kinase activity | 3.02E-07 | 1.67E-05 | 1.22E-05 |
| BP | GO:0010466 | negative regulation of peptidase activity | 3.18E-07 | 1.74E-05 | 1.27E-05 |
| BP | GO:0051056 | regulation of small GTPase mediated signal transduction | 3.29E-07 | 1.79E-05 | 1.31E-05 |
| BP | GO:0006024 | glycosaminoglycan biosynthetic process | 3.34E-07 | 1.80E-05 | 1.32E-05 |
| BP | GO:0018149 | peptide cross-linking | 3.52E-07 | 1.88E-05 | 1.37E-05 |
| BP | GO:0032970 | regulation of actin filament-based process | 3.54E-07 | 1.88E-05 | 1.37E-05 |
| BP | GO:0048732 | gland development | 3.74E-07 | 1.96E-05 | 1.43E-05 |
| BP | GO:0060541 | respiratory system development | 3.84E-07 | 2.00E-05 | 1.46E-05 |
| BP | GO:0030324 | lung development | 4.64E-07 | 2.39E-05 | 1.75E-05 |
| BP | GO:0050866 | negative regulation of cell activation | 4.88E-07 | 2.50E-05 | 1.83E-05 |
| BP | GO:0060349 | bone morphogenesis | 5.13E-07 | 2.60E-05 | 1.90E-05 |
| BP | GO:0033002 | muscle cell proliferation | 5.27E-07 | 2.65E-05 | 1.94E-05 |
| BP | GO:0032956 | regulation of actin cytoskeleton organization | 5.58E-07 | 2.79E-05 | 2.04E-05 |
| BP | GO:1990778 | protein localization to cell periphery | 5.67E-07 | 2.81E-05 | 2.05E-05 |
| BP | GO:0035107 | appendage morphogenesis | 6.17E-07 | 3.01E-05 | 2.20E-05 |
| BP | GO:0035108 | limb morphogenesis | 6.17E-07 | 3.01E-05 | 2.20E-05 |
| BP | GO:0010951 | negative regulation of endopeptidase activity | 6.22E-07 | 3.01E-05 | 2.20E-05 |
| BP | GO:0051099 | positive regulation of binding | 6.50E-07 | 3.12E-05 | 2.28E-05 |
| BP | GO:0060284 | regulation of cell development | 7.16E-07 | 3.41E-05 | 2.49E-05 |
| BP | GO:0032496 | response to lipopolysaccharide | 7.99E-07 | 3.74E-05 | 2.74E-05 |
| BP | GO:0003203 | endocardial cushion morphogenesis | 8.04E-07 | 3.74E-05 | 2.74E-05 |
| BP | GO:0035909 | aorta morphogenesis | 8.04E-07 | 3.74E-05 | 2.74E-05 |
| BP | GO:0051051 | negative regulation of transport | 8.11E-07 | 3.75E-05 | 2.74E-05 |
| BP | GO:0150115 | cell-substrate junction organization | 8.36E-07 | 3.83E-05 | 2.80E-05 |
| BP | GO:0030516 | regulation of axon extension | 8.73E-07 | 3.94E-05 | 2.88E-05 |
| BP | GO:1901655 | cellular response to ketone | 8.73E-07 | 3.94E-05 | 2.88E-05 |
| BP | GO:0003205 | cardiac chamber development | 8.90E-07 | 3.99E-05 | 2.92E-05 |
| BP | GO:0010594 | regulation of endothelial cell migration | 9.06E-07 | 4.03E-05 | 2.95E-05 |
| BP | GO:0030323 | respiratory tube development | 9.72E-07 | 4.17E-05 | 3.05E-05 |
| BP | GO:0048736 | appendage development | 9.72E-07 | 4.17E-05 | 3.05E-05 |
| BP | GO:0060173 | limb development | 9.72E-07 | 4.17E-05 | 3.05E-05 |
| BP | GO:0071772 | response to BMP | 9.72E-07 | 4.17E-05 | 3.05E-05 |
| BP | GO:0071773 | cellular response to BMP stimulus | 9.72E-07 | 4.17E-05 | 3.05E-05 |
| BP | GO:0061448 | connective tissue development | 1.04E-06 | 4.43E-05 | 3.24E-05 |
| BP | GO:0030168 | platelet activation | 1.05E-06 | 4.43E-05 | 3.24E-05 |
| BP | GO:0002697 | regulation of immune effector process | 1.09E-06 | 4.53E-05 | 3.31E-05 |
| BP | GO:0045861 | negative regulation of proteolysis | 1.09E-06 | 4.53E-05 | 3.31E-05 |
| BP | GO:0048608 | reproductive structure development | 1.10E-06 | 4.53E-05 | 3.31E-05 |
| BP | GO:0085029 | extracellular matrix assembly | 1.10E-06 | 4.53E-05 | 3.31E-05 |
| BP | GO:0048588 | developmental cell growth | 1.22E-06 | 5.00E-05 | 3.66E-05 |
| BP | GO:1901888 | regulation of cell junction assembly | 1.25E-06 | 5.10E-05 | 3.73E-05 |
| BP | GO:0046578 | regulation of Ras protein signal transduction | 1.33E-06 | 5.36E-05 | 3.92E-05 |
| BP | GO:0001569 | branching involved in blood vessel morphogenesis | 1.34E-06 | 5.36E-05 | 3.92E-05 |
| BP | GO:0052547 | regulation of peptidase activity | 1.35E-06 | 5.38E-05 | 3.93E-05 |
| BP | GO:0002687 | positive regulation of leukocyte migration | 1.41E-06 | 5.60E-05 | 4.09E-05 |
| BP | GO:0002576 | platelet degranulation | 1.42E-06 | 5.60E-05 | 4.09E-05 |
| BP | GO:0061458 | reproductive system development | 1.51E-06 | 5.92E-05 | 4.33E-05 |
| BP | GO:0030509 | BMP signaling pathway | 1.57E-06 | 6.00E-05 | 4.39E-05 |
| BP | GO:1902107 | positive regulation of leukocyte differentiation | 1.57E-06 | 6.00E-05 | 4.39E-05 |
| BP | GO:1903708 | positive regulation of hemopoiesis | 1.57E-06 | 6.00E-05 | 4.39E-05 |
| BP | GO:0051897 | positive regulation of protein kinase B signaling | 1.57E-06 | 6.00E-05 | 4.39E-05 |
| BP | GO:0061387 | regulation of extent of cell growth | 1.62E-06 | 6.16E-05 | 4.50E-05 |
| BP | GO:0031214 | biomineral tissue development | 1.81E-06 | 6.82E-05 | 4.99E-05 |
| BP | GO:0003180 | aortic valve morphogenesis | 1.84E-06 | 6.90E-05 | 5.04E-05 |
| BP | GO:0007044 | cell-substrate junction assembly | 1.97E-06 | 7.33E-05 | 5.36E-05 |
| BP | GO:0150063 | visual system development | 2.00E-06 | 7.40E-05 | 5.41E-05 |
| BP | GO:0048880 | sensory system development | 2.02E-06 | 7.42E-05 | 5.43E-05 |
| BP | GO:0002703 | regulation of leukocyte mediated immunity | 2.06E-06 | 7.53E-05 | 5.50E-05 |
| BP | GO:0002237 | response to molecule of bacterial origin | 2.16E-06 | 7.80E-05 | 5.70E-05 |
| BP | GO:0007599 | hemostasis | 2.16E-06 | 7.80E-05 | 5.70E-05 |
| BP | GO:0035904 | aorta development | 2.19E-06 | 7.85E-05 | 5.74E-05 |
| BP | GO:1903053 | regulation of extracellular matrix organization | 2.43E-06 | 8.66E-05 | 6.33E-05 |
| BP | GO:0110148 | biomineralization | 2.58E-06 | 9.16E-05 | 6.70E-05 |
| BP | GO:0007596 | blood coagulation | 2.68E-06 | 9.44E-05 | 6.90E-05 |
| BP | GO:0150116 | regulation of cell-substrate junction organization | 2.95E-06 | 0.000104 | 7.58E-05 |
| BP | GO:0060760 | positive regulation of response to cytokine stimulus | 3.07E-06 | 0.000107 | 7.83E-05 |
| BP | GO:0031349 | positive regulation of defense response | 3.09E-06 | 0.000107 | 7.83E-05 |
| BP | GO:0050870 | positive regulation of T cell activation | 3.23E-06 | 0.000111 | 8.10E-05 |
| BP | GO:2000050 | regulation of non-canonical Wnt signaling pathway | 3.24E-06 | 0.000111 | 8.10E-05 |
| BP | GO:0030326 | embryonic limb morphogenesis | 3.27E-06 | 0.000111 | 8.10E-05 |
| BP | GO:0035113 | embryonic appendage morphogenesis | 3.27E-06 | 0.000111 | 8.10E-05 |
| BP | GO:0071559 | response to transforming growth factor beta | 3.32E-06 | 0.000112 | 8.20E-05 |
| BP | GO:0035567 | non-canonical Wnt signaling pathway | 3.37E-06 | 0.000113 | 8.27E-05 |
| BP | GO:0051216 | cartilage development | 3.39E-06 | 0.000113 | 8.28E-05 |
| BP | GO:0033628 | regulation of cell adhesion mediated by integrin | 3.53E-06 | 0.000117 | 8.56E-05 |
| BP | GO:0035987 | endodermal cell differentiation | 3.64E-06 | 0.00012 | 8.78E-05 |
| BP | GO:0010634 | positive regulation of epithelial cell migration | 3.85E-06 | 0.000126 | 9.24E-05 |
| BP | GO:0051017 | actin filament bundle assembly | 4.02E-06 | 0.000131 | 9.61E-05 |
| BP | GO:0001886 | endothelial cell morphogenesis | 4.48E-06 | 0.000146 | 0.000107 |
| BP | GO:0048660 | regulation of smooth muscle cell proliferation | 4.56E-06 | 0.000147 | 0.000108 |
| BP | GO:0050817 | coagulation | 4.68E-06 | 0.000151 | 0.00011 |
| BP | GO:0070661 | leukocyte proliferation | 4.87E-06 | 0.000156 | 0.000114 |
| BP | GO:0051090 | regulation of DNA-binding transcription factor activity | 4.98E-06 | 0.000159 | 0.000116 |
| BP | GO:0032835 | glomerulus development | 5.21E-06 | 0.000165 | 0.000121 |
| BP | GO:0048041 | focal adhesion assembly | 5.31E-06 | 0.000167 | 0.000122 |
| BP | GO:0090287 | regulation of cellular response to growth factor stimulus | 5.83E-06 | 0.000183 | 0.000134 |
| BP | GO:0031663 | lipopolysaccharide-mediated signaling pathway | 5.88E-06 | 0.000183 | 0.000134 |
| BP | GO:0050770 | regulation of axonogenesis | 5.99E-06 | 0.000186 | 0.000136 |
| BP | GO:0002832 | negative regulation of response to biotic stimulus | 6.02E-06 | 0.000186 | 0.000136 |
| BP | GO:1902105 | regulation of leukocyte differentiation | 6.13E-06 | 0.000187 | 0.000136 |
| BP | GO:0001704 | formation of primary germ layer | 6.14E-06 | 0.000187 | 0.000136 |
| BP | GO:0003206 | cardiac chamber morphogenesis | 6.14E-06 | 0.000187 | 0.000136 |
| BP | GO:0048659 | smooth muscle cell proliferation | 6.39E-06 | 0.000193 | 0.000141 |
| BP | GO:0001933 | negative regulation of protein phosphorylation | 6.53E-06 | 0.000197 | 0.000144 |
| BP | GO:0007015 | actin filament organization | 6.78E-06 | 0.000202 | 0.000148 |
| BP | GO:0030100 | regulation of endocytosis | 6.79E-06 | 0.000202 | 0.000148 |
| BP | GO:0050863 | regulation of T cell activation | 7.04E-06 | 0.000209 | 0.000153 |
| BP | GO:0007369 | gastrulation | 7.30E-06 | 0.000214 | 0.000157 |
| BP | GO:0090090 | negative regulation of canonical Wnt signaling pathway | 7.30E-06 | 0.000214 | 0.000157 |
| BP | GO:0010811 | positive regulation of cell-substrate adhesion | 7.40E-06 | 0.000217 | 0.000158 |
| BP | GO:0007043 | cell-cell junction assembly | 7.49E-06 | 0.000218 | 0.000159 |
| BP | GO:2000379 | positive regulation of reactive oxygen species metabolic process | 7.51E-06 | 0.000218 | 0.000159 |
| BP | GO:0072009 | nephron epithelium development | 7.59E-06 | 0.000218 | 0.000159 |
| BP | GO:0022612 | gland morphogenesis | 7.59E-06 | 0.000218 | 0.000159 |
| BP | GO:1904062 | regulation of cation transmembrane transport | 7.97E-06 | 0.000227 | 0.000166 |
| BP | GO:0061572 | actin filament bundle organization | 8.00E-06 | 0.000227 | 0.000166 |
| BP | GO:1902903 | regulation of supramolecular fiber organization | 8.04E-06 | 0.000227 | 0.000166 |
| BP | GO:0070663 | regulation of leukocyte proliferation | 8.07E-06 | 0.000227 | 0.000166 |
| BP | GO:0001819 | positive regulation of cytokine production | 8.19E-06 | 0.00023 | 0.000168 |
| BP | GO:0051250 | negative regulation of lymphocyte activation | 8.46E-06 | 0.000236 | 0.000173 |
| BP | GO:0001654 | eye development | 8.93E-06 | 0.000247 | 0.000181 |
| BP | GO:0042493 | response to drug | 8.93E-06 | 0.000247 | 0.000181 |
| BP | GO:0061299 | retina vasculature morphogenesis in camera-type eye | 9.09E-06 | 0.000248 | 0.000181 |
| BP | GO:0045766 | positive regulation of angiogenesis | 9.14E-06 | 0.000248 | 0.000181 |
| BP | GO:1904018 | positive regulation of vasculature development | 9.14E-06 | 0.000248 | 0.000181 |
| BP | GO:0071222 | cellular response to lipopolysaccharide | 9.20E-06 | 0.000248 | 0.000181 |
| BP | GO:0045807 | positive regulation of endocytosis | 9.21E-06 | 0.000248 | 0.000181 |
| BP | GO:0032231 | regulation of actin filament bundle assembly | 9.34E-06 | 0.000248 | 0.000181 |
| BP | GO:0042116 | macrophage activation | 9.34E-06 | 0.000248 | 0.000181 |
| BP | GO:0003281 | ventricular septum development | 9.41E-06 | 0.000248 | 0.000181 |
| BP | GO:0051893 | regulation of focal adhesion assembly | 9.41E-06 | 0.000248 | 0.000181 |
| BP | GO:0060411 | cardiac septum morphogenesis | 9.41E-06 | 0.000248 | 0.000181 |
| BP | GO:0090109 | regulation of cell-substrate junction assembly | 9.41E-06 | 0.000248 | 0.000181 |
| BP | GO:0048562 | embryonic organ morphogenesis | 9.71E-06 | 0.000255 | 0.000186 |
| BP | GO:0043277 | apoptotic cell clearance | 1.01E-05 | 0.000263 | 0.000192 |
| BP | GO:0006469 | negative regulation of protein kinase activity | 1.05E-05 | 0.000272 | 0.000199 |
| BP | GO:0002573 | myeloid leukocyte differentiation | 1.13E-05 | 0.000293 | 0.000214 |
| BP | GO:0010720 | positive regulation of cell development | 1.14E-05 | 0.000293 | 0.000214 |
| BP | GO:0042692 | muscle cell differentiation | 1.22E-05 | 0.000313 | 0.000229 |
| BP | GO:0050730 | regulation of peptidyl-tyrosine phosphorylation | 1.30E-05 | 0.000333 | 0.000243 |
| BP | GO:1905523 | positive regulation of macrophage migration | 1.32E-05 | 0.000336 | 0.000246 |
| BP | GO:0003018 | vascular process in circulatory system | 1.35E-05 | 0.000343 | 0.000251 |
| BP | GO:0051492 | regulation of stress fiber assembly | 1.38E-05 | 0.000348 | 0.000254 |
| BP | GO:0090092 | regulation of transmembrane receptor protein serine/threonine kinase signaling pathway | 1.39E-05 | 0.000349 | 0.000255 |
| BP | GO:0050819 | negative regulation of coagulation | 1.39E-05 | 0.000349 | 0.000255 |
| BP | GO:0070167 | regulation of biomineral tissue development | 1.41E-05 | 0.000352 | 0.000257 |
| BP | GO:0001890 | placenta development | 1.46E-05 | 0.000363 | 0.000266 |
| BP | GO:0072659 | protein localization to plasma membrane | 1.53E-05 | 0.000379 | 0.000277 |
| BP | GO:0002698 | negative regulation of immune effector process | 1.59E-05 | 0.000391 | 0.000286 |
| BP | GO:0071674 | mononuclear cell migration | 1.67E-05 | 0.000408 | 0.000298 |
| BP | GO:0062197 | cellular response to chemical stress | 1.67E-05 | 0.000408 | 0.000298 |
| BP | GO:0003177 | pulmonary valve development | 1.73E-05 | 0.00042 | 0.000307 |
| BP | GO:0016049 | cell growth | 1.73E-05 | 0.00042 | 0.000307 |
| BP | GO:0050818 | regulation of coagulation | 1.81E-05 | 0.000437 | 0.00032 |
| BP | GO:0007224 | smoothened signaling pathway | 1.83E-05 | 0.00044 | 0.000322 |
| BP | GO:0001961 | positive regulation of cytokine-mediated signaling pathway | 2.14E-05 | 0.000507 | 0.000371 |
| BP | GO:1900047 | negative regulation of hemostasis | 2.14E-05 | 0.000507 | 0.000371 |
| BP | GO:0051495 | positive regulation of cytoskeleton organization | 2.15E-05 | 0.000507 | 0.000371 |
| BP | GO:1900046 | regulation of hemostasis | 2.15E-05 | 0.000507 | 0.000371 |
| BP | GO:0030510 | regulation of BMP signaling pathway | 2.15E-05 | 0.000507 | 0.000371 |
| BP | GO:0014812 | muscle cell migration | 2.17E-05 | 0.000507 | 0.000371 |
| BP | GO:0048010 | vascular endothelial growth factor receptor signaling pathway | 2.17E-05 | 0.000507 | 0.000371 |
| BP | GO:0110149 | regulation of biomineralization | 2.17E-05 | 0.000507 | 0.000371 |
| BP | GO:0071219 | cellular response to molecule of bacterial origin | 2.25E-05 | 0.000524 | 0.000383 |
| BP | GO:0002819 | regulation of adaptive immune response | 2.28E-05 | 0.000527 | 0.000385 |
| BP | GO:0003002 | regionalization | 2.28E-05 | 0.000527 | 0.000385 |
| BP | GO:0001974 | blood vessel remodeling | 2.37E-05 | 0.000544 | 0.000398 |
| BP | GO:0110053 | regulation of actin filament organization | 2.40E-05 | 0.000549 | 0.000401 |
| BP | GO:1901654 | response to ketone | 2.55E-05 | 0.000582 | 0.000426 |
| BP | GO:0051346 | negative regulation of hydrolase activity | 2.60E-05 | 0.000591 | 0.000432 |
| BP | GO:0071216 | cellular response to biotic stimulus | 2.61E-05 | 0.000592 | 0.000433 |
| BP | GO:0060412 | ventricular septum morphogenesis | 2.67E-05 | 0.000603 | 0.000441 |
| BP | GO:0045667 | regulation of osteoblast differentiation | 2.73E-05 | 0.000613 | 0.000448 |
| BP | GO:0001738 | morphogenesis of a polarized epithelium | 2.74E-05 | 0.000614 | 0.000449 |
| BP | GO:0045926 | negative regulation of growth | 2.76E-05 | 0.000616 | 0.00045 |
| BP | GO:0007568 | aging | 2.80E-05 | 0.000623 | 0.000455 |
| BP | GO:0006898 | receptor-mediated endocytosis | 2.81E-05 | 0.000623 | 0.000455 |
| BP | GO:0007389 | pattern specification process | 2.86E-05 | 0.000632 | 0.000462 |
| BP | GO:0003151 | outflow tract morphogenesis | 2.99E-05 | 0.000659 | 0.000482 |
| BP | GO:0050777 | negative regulation of immune response | 3.02E-05 | 0.000663 | 0.000485 |
| BP | GO:0022408 | negative regulation of cell-cell adhesion | 3.19E-05 | 0.000697 | 0.00051 |
| BP | GO:0110020 | regulation of actomyosin structure organization | 3.29E-05 | 0.000717 | 0.000524 |
| BP | GO:0010171 | body morphogenesis | 3.30E-05 | 0.000717 | 0.000524 |
| BP | GO:0001666 | response to hypoxia | 3.39E-05 | 0.000732 | 0.000535 |
| BP | GO:0016331 | morphogenesis of embryonic epithelium | 3.43E-05 | 0.00074 | 0.000541 |
| BP | GO:0003231 | cardiac ventricle development | 3.71E-05 | 0.000797 | 0.000582 |
| BP | GO:0031348 | negative regulation of defense response | 3.78E-05 | 0.000809 | 0.000591 |
| BP | GO:0030038 | contractile actin filament bundle assembly | 3.95E-05 | 0.000839 | 0.000613 |
| BP | GO:0043149 | stress fiber assembly | 3.95E-05 | 0.000839 | 0.000613 |
| BP | GO:0071711 | basement membrane organization | 3.97E-05 | 0.00084 | 0.000614 |
| BP | GO:0010543 | regulation of platelet activation | 4.02E-05 | 0.000848 | 0.00062 |
| BP | GO:0035023 | regulation of Rho protein signal transduction | 4.05E-05 | 0.000852 | 0.000623 |
| BP | GO:0001952 | regulation of cell-matrix adhesion | 4.06E-05 | 0.000852 | 0.000623 |
| BP | GO:0031346 | positive regulation of cell projection organization | 4.15E-05 | 0.000867 | 0.000634 |
| BP | GO:0001935 | endothelial cell proliferation | 4.20E-05 | 0.000871 | 0.000637 |
| BP | GO:0031032 | actomyosin structure organization | 4.20E-05 | 0.000871 | 0.000637 |
| BP | GO:0006909 | phagocytosis | 4.27E-05 | 0.000882 | 0.000645 |
| BP | GO:0030574 | collagen catabolic process | 4.55E-05 | 0.000938 | 0.000686 |
| BP | GO:0033673 | negative regulation of kinase activity | 4.61E-05 | 0.000944 | 0.00069 |
| BP | GO:0051145 | smooth muscle cell differentiation | 4.62E-05 | 0.000944 | 0.00069 |
| BP | GO:0006979 | response to oxidative stress | 4.63E-05 | 0.000944 | 0.00069 |
| BP | GO:0042326 | negative regulation of phosphorylation | 4.79E-05 | 0.000974 | 0.000712 |
| BP | GO:0010717 | regulation of epithelial to mesenchymal transition | 4.91E-05 | 0.000995 | 0.000728 |
| BP | GO:0003198 | epithelial to mesenchymal transition involved in endocardial cushion formation | 4.96E-05 | 0.001003 | 0.000733 |
| BP | GO:0019915 | lipid storage | 5.04E-05 | 0.001013 | 0.00074 |
| BP | GO:0061326 | renal tubule development | 5.05E-05 | 0.001013 | 0.00074 |
| BP | GO:0003183 | mitral valve morphogenesis | 5.14E-05 | 0.001028 | 0.000752 |
| BP | GO:0043010 | camera-type eye development | 5.24E-05 | 0.001046 | 0.000764 |
| BP | GO:0030879 | mammary gland development | 5.39E-05 | 0.001072 | 0.000784 |
| BP | GO:1905521 | regulation of macrophage migration | 5.56E-05 | 0.001089 | 0.000796 |
| BP | GO:0060841 | venous blood vessel development | 5.56E-05 | 0.001089 | 0.000796 |
| BP | GO:1902285 | semaphorin-plexin signaling pathway involved in neuron projection guidance | 5.56E-05 | 0.001089 | 0.000796 |
| BP | GO:0030193 | regulation of blood coagulation | 5.57E-05 | 0.001089 | 0.000796 |
| BP | GO:0048863 | stem cell differentiation | 5.58E-05 | 0.001089 | 0.000796 |
| BP | GO:0071560 | cellular response to transforming growth factor beta stimulus | 5.58E-05 | 0.001089 | 0.000796 |
| BP | GO:0002695 | negative regulation of leukocyte activation | 5.73E-05 | 0.001114 | 0.000815 |
| BP | GO:0007517 | muscle organ development | 5.78E-05 | 0.001119 | 0.000818 |
| BP | GO:0071902 | positive regulation of protein serine/threonine kinase activity | 5.80E-05 | 0.001121 | 0.00082 |
| BP | GO:0036293 | response to decreased oxygen levels | 5.96E-05 | 0.001147 | 0.000838 |
| BP | GO:0030195 | negative regulation of blood coagulation | 6.20E-05 | 0.001182 | 0.000864 |
| BP | GO:0061028 | establishment of endothelial barrier | 6.20E-05 | 0.001182 | 0.000864 |
| BP | GO:0022617 | extracellular matrix disassembly | 6.20E-05 | 0.001182 | 0.000864 |
| BP | GO:0043434 | response to peptide hormone | 6.23E-05 | 0.001182 | 0.000864 |
| BP | GO:0060021 | roof of mouth development | 6.24E-05 | 0.001182 | 0.000864 |
| BP | GO:0001936 | regulation of endothelial cell proliferation | 6.25E-05 | 0.001182 | 0.000864 |
| BP | GO:0050729 | positive regulation of inflammatory response | 6.36E-05 | 0.001197 | 0.000875 |
| BP | GO:0002822 | regulation of adaptive immune response based on somatic recombination of immune receptors built from immunoglobulin superfamily domains | 6.52E-05 | 0.001225 | 0.000896 |
| BP | GO:0006022 | aminoglycan metabolic process | 6.81E-05 | 0.001276 | 0.000933 |
| BP | GO:0042110 | T cell activation | 6.87E-05 | 0.001283 | 0.000938 |
| BP | GO:0090100 | positive regulation of transmembrane receptor protein serine/threonine kinase signaling pathway | 6.96E-05 | 0.001295 | 0.000947 |
| BP | GO:0043087 | regulation of GTPase activity | 7.05E-05 | 0.001304 | 0.000953 |
| BP | GO:0048638 | regulation of developmental growth | 7.05E-05 | 0.001304 | 0.000953 |
| BP | GO:0150146 | cell junction disassembly | 7.08E-05 | 0.001306 | 0.000955 |
| BP | GO:0010812 | negative regulation of cell-substrate adhesion | 7.13E-05 | 0.001311 | 0.000959 |
| BP | GO:0001706 | endoderm formation | 7.18E-05 | 0.001311 | 0.000959 |
| BP | GO:1905517 | macrophage migration | 7.18E-05 | 0.001311 | 0.000959 |
| BP | GO:0003015 | heart process | 7.19E-05 | 0.001311 | 0.000959 |
| BP | GO:0050867 | positive regulation of cell activation | 7.40E-05 | 0.001345 | 0.000983 |
| BP | GO:0060041 | retina development in camera-type eye | 7.59E-05 | 0.001375 | 0.001005 |
| BP | GO:0071526 | semaphorin-plexin signaling pathway | 7.83E-05 | 0.001415 | 0.001035 |
| BP | GO:0051348 | negative regulation of transferase activity | 7.96E-05 | 0.001432 | 0.001047 |
| BP | GO:0030282 | bone mineralization | 7.97E-05 | 0.001432 | 0.001047 |
| BP | GO:0052548 | regulation of endopeptidase activity | 8.38E-05 | 0.001495 | 0.001093 |
| BP | GO:0009612 | response to mechanical stimulus | 8.39E-05 | 0.001495 | 0.001093 |
| BP | GO:0060070 | canonical Wnt signaling pathway | 8.40E-05 | 0.001495 | 0.001093 |
| BP | GO:0035136 | forelimb morphogenesis | 8.51E-05 | 0.001511 | 0.001105 |
| BP | GO:0031532 | actin cytoskeleton reorganization | 8.70E-05 | 0.001535 | 0.001122 |
| BP | GO:0032526 | response to retinoic acid | 8.70E-05 | 0.001535 | 0.001122 |
| BP | GO:0016525 | negative regulation of angiogenesis | 8.84E-05 | 0.001555 | 0.001137 |
| BP | GO:0043491 | protein kinase B signaling | 8.86E-05 | 0.001555 | 0.001137 |
| BP | GO:0044331 | cell-cell adhesion mediated by cadherin | 8.98E-05 | 0.001569 | 0.001147 |
| BP | GO:0002688 | regulation of leukocyte chemotaxis | 8.99E-05 | 0.001569 | 0.001147 |
| BP | GO:0043254 | regulation of protein-containing complex assembly | 9.18E-05 | 0.001597 | 0.001168 |
| BP | GO:0010759 | positive regulation of macrophage chemotaxis | 9.33E-05 | 0.001614 | 0.00118 |
| BP | GO:0030903 | notochord development | 9.33E-05 | 0.001614 | 0.00118 |
| BP | GO:0072080 | nephron tubule development | 9.43E-05 | 0.001627 | 0.00119 |
| BP | GO:0001838 | embryonic epithelial tube formation | 9.50E-05 | 0.001631 | 0.001192 |
| BP | GO:1902905 | positive regulation of supramolecular fiber organization | 9.51E-05 | 0.001631 | 0.001192 |
| BP | GO:0003012 | muscle system process | 9.69E-05 | 0.001658 | 0.001212 |
| BP | GO:1902414 | protein localization to cell junction | 9.98E-05 | 0.001701 | 0.001244 |
| BP | GO:0072175 | epithelial tube formation | 0.0001 | 0.001701 | 0.001244 |
| BP | GO:0030203 | glycosaminoglycan metabolic process | 0.000101 | 0.001714 | 0.001253 |
| BP | GO:0070482 | response to oxygen levels | 0.000106 | 0.001797 | 0.001314 |
| BP | GO:0050767 | regulation of neurogenesis | 0.000108 | 0.001825 | 0.001334 |
| BP | GO:0061041 | regulation of wound healing | 0.00011 | 0.001844 | 0.001348 |
| BP | GO:0072073 | kidney epithelium development | 0.00011 | 0.001844 | 0.001348 |
| BP | GO:0050764 | regulation of phagocytosis | 0.000112 | 0.001879 | 0.001374 |
| BP | GO:1903510 | mucopolysaccharide metabolic process | 0.000113 | 0.001882 | 0.001376 |
| BP | GO:0050731 | positive regulation of peptidyl-tyrosine phosphorylation | 0.000115 | 0.001915 | 0.0014 |
| BP | GO:0060326 | cell chemotaxis | 0.000118 | 0.001956 | 0.00143 |
| BP | GO:2000181 | negative regulation of blood vessel morphogenesis | 0.000119 | 0.001967 | 0.001438 |
| BP | GO:0035455 | response to interferon-alpha | 0.000119 | 0.001967 | 0.001438 |
| BP | GO:0036303 | lymph vessel morphogenesis | 0.000119 | 0.001967 | 0.001438 |
| BP | GO:0007584 | response to nutrient | 0.000123 | 0.002015 | 0.001473 |
| BP | GO:0003174 | mitral valve development | 0.000125 | 0.002044 | 0.001494 |
| BP | GO:0060837 | blood vessel endothelial cell differentiation | 0.000125 | 0.002044 | 0.001494 |
| BP | GO:0061314 | Notch signaling involved in heart development | 0.000125 | 0.002044 | 0.001494 |
| BP | GO:0051235 | maintenance of location | 0.000127 | 0.002065 | 0.00151 |
| BP | GO:0010595 | positive regulation of endothelial cell migration | 0.000129 | 0.002083 | 0.001523 |
| BP | GO:0035633 | maintenance of blood-brain barrier | 0.00013 | 0.002104 | 0.001538 |
| BP | GO:0006939 | smooth muscle contraction | 0.000131 | 0.002107 | 0.00154 |
| BP | GO:0030099 | myeloid cell differentiation | 0.000133 | 0.002134 | 0.00156 |
| BP | GO:1904019 | epithelial cell apoptotic process | 0.000133 | 0.00214 | 0.001565 |
| BP | GO:0060325 | face morphogenesis | 0.000135 | 0.002161 | 0.00158 |
| BP | GO:0060337 | type I interferon signaling pathway | 0.000136 | 0.002166 | 0.001584 |
| BP | GO:1901343 | negative regulation of vasculature development | 0.000138 | 0.002188 | 0.0016 |
| BP | GO:0014033 | neural crest cell differentiation | 0.00014 | 0.002223 | 0.001625 |
| BP | GO:0071675 | regulation of mononuclear cell migration | 0.000141 | 0.002238 | 0.001636 |
| BP | GO:0002696 | positive regulation of leukocyte activation | 0.000143 | 0.002253 | 0.001647 |
| BP | GO:0045123 | cellular extravasation | 0.000144 | 0.002267 | 0.001657 |
| BP | GO:0000302 | response to reactive oxygen species | 0.000144 | 0.002267 | 0.001657 |
| BP | GO:1990823 | response to leukemia inhibitory factor | 0.000145 | 0.002275 | 0.001664 |
| BP | GO:0051668 | localization within membrane | 0.000146 | 0.002275 | 0.001664 |
| BP | GO:0043406 | positive regulation of MAP kinase activity | 0.000146 | 0.002276 | 0.001664 |
| BP | GO:1903522 | regulation of blood circulation | 0.000147 | 0.002282 | 0.001669 |
| BP | GO:0010718 | positive regulation of epithelial to mesenchymal transition | 0.000147 | 0.002282 | 0.001669 |
| BP | GO:0051091 | positive regulation of DNA-binding transcription factor activity | 0.00015 | 0.002313 | 0.001691 |
| BP | GO:0071357 | cellular response to type I interferon | 0.000164 | 0.002523 | 0.001845 |
| BP | GO:0032147 | activation of protein kinase activity | 0.000166 | 0.002552 | 0.001866 |
| BP | GO:0030856 | regulation of epithelial cell differentiation | 0.000168 | 0.00256 | 0.001872 |
| BP | GO:0010463 | mesenchymal cell proliferation | 0.000168 | 0.00256 | 0.001872 |
| BP | GO:0010742 | macrophage derived foam cell differentiation | 0.000168 | 0.00256 | 0.001872 |
| BP | GO:0030204 | chondroitin sulfate metabolic process | 0.000168 | 0.00256 | 0.001872 |
| BP | GO:0090077 | foam cell differentiation | 0.000168 | 0.00256 | 0.001872 |
| BP | GO:0002690 | positive regulation of leukocyte chemotaxis | 0.00017 | 0.002573 | 0.001881 |
| BP | GO:0071230 | cellular response to amino acid stimulus | 0.00017 | 0.002575 | 0.001883 |
| BP | GO:0007179 | transforming growth factor beta receptor signaling pathway | 0.000171 | 0.002575 | 0.001883 |
| BP | GO:0001736 | establishment of planar polarity | 0.000173 | 0.002589 | 0.001893 |
| BP | GO:0007164 | establishment of tissue polarity | 0.000173 | 0.002589 | 0.001893 |
| BP | GO:1903409 | reactive oxygen species biosynthetic process | 0.000173 | 0.002589 | 0.001893 |
| BP | GO:0030225 | macrophage differentiation | 0.000173 | 0.002589 | 0.001893 |
| BP | GO:0055123 | digestive system development | 0.000175 | 0.002603 | 0.001903 |
| BP | GO:0030308 | negative regulation of cell growth | 0.000179 | 0.002663 | 0.001947 |
| BP | GO:0007492 | endoderm development | 0.00018 | 0.002672 | 0.001954 |
| BP | GO:0035148 | tube formation | 0.000187 | 0.00277 | 0.002025 |
| BP | GO:0050679 | positive regulation of epithelial cell proliferation | 0.000192 | 0.002828 | 0.002067 |
| BP | GO:0051496 | positive regulation of stress fiber assembly | 0.000192 | 0.002828 | 0.002067 |
| BP | GO:0060350 | endochondral bone morphogenesis | 0.000192 | 0.002828 | 0.002067 |
| BP | GO:0001818 | negative regulation of cytokine production | 0.000193 | 0.002833 | 0.002071 |
| BP | GO:0050804 | modulation of chemical synaptic transmission | 0.000198 | 0.002886 | 0.00211 |
| BP | GO:0060828 | regulation of canonical Wnt signaling pathway | 0.000199 | 0.002886 | 0.00211 |
| BP | GO:0060674 | placenta blood vessel development | 0.000199 | 0.002886 | 0.00211 |
| BP | GO:0070168 | negative regulation of biomineral tissue development | 0.000199 | 0.002886 | 0.00211 |
| BP | GO:0034142 | toll-like receptor 4 signaling pathway | 0.000202 | 0.002914 | 0.00213 |
| BP | GO:0061383 | trabecula morphogenesis | 0.000202 | 0.002914 | 0.00213 |
| BP | GO:0001945 | lymph vessel development | 0.000203 | 0.002914 | 0.00213 |
| BP | GO:0061437 | renal system vasculature development | 0.000203 | 0.002914 | 0.00213 |
| BP | GO:0061440 | kidney vasculature development | 0.000203 | 0.002914 | 0.00213 |
| BP | GO:0048015 | phosphatidylinositol-mediated signaling | 0.000208 | 0.002969 | 0.002171 |
| BP | GO:2000377 | regulation of reactive oxygen species metabolic process | 0.000208 | 0.002969 | 0.002171 |
| BP | GO:0001558 | regulation of cell growth | 0.000215 | 0.00305 | 0.00223 |
| BP | GO:0099177 | regulation of trans-synaptic signaling | 0.000215 | 0.00305 | 0.00223 |
| BP | GO:0032233 | positive regulation of actin filament bundle assembly | 0.000216 | 0.003066 | 0.002241 |
| BP | GO:0043534 | blood vessel endothelial cell migration | 0.000217 | 0.003068 | 0.002243 |
| BP | GO:2000095 | regulation of Wnt signaling pathway, planar cell polarity pathway | 0.000218 | 0.003076 | 0.002249 |
| BP | GO:2001044 | regulation of integrin-mediated signaling pathway | 0.000218 | 0.003076 | 0.002249 |
| BP | GO:1901224 | positive regulation of NIK/NF-kappaB signaling | 0.000221 | 0.003101 | 0.002267 |
| BP | GO:0043407 | negative regulation of MAP kinase activity | 0.000222 | 0.003101 | 0.002267 |
| BP | GO:0046209 | nitric oxide metabolic process | 0.000222 | 0.003101 | 0.002267 |
| BP | GO:0035924 | cellular response to vascular endothelial growth factor stimulus | 0.000223 | 0.003112 | 0.002275 |
| BP | GO:0043409 | negative regulation of MAPK cascade | 0.000223 | 0.003112 | 0.002275 |
| BP | GO:0043271 | negative regulation of ion transport | 0.000225 | 0.003126 | 0.002285 |
| BP | GO:0035850 | epithelial cell differentiation involved in kidney development | 0.000229 | 0.003166 | 0.002315 |
| BP | GO:2001238 | positive regulation of extrinsic apoptotic signaling pathway | 0.000229 | 0.003166 | 0.002315 |
| BP | GO:1903034 | regulation of response to wounding | 0.00023 | 0.003171 | 0.002318 |
| BP | GO:2000027 | regulation of animal organ morphogenesis | 0.000246 | 0.003388 | 0.002477 |
| BP | GO:0050769 | positive regulation of neurogenesis | 0.000248 | 0.003412 | 0.002495 |
| BP | GO:0051896 | regulation of protein kinase B signaling | 0.000253 | 0.003462 | 0.002531 |
| BP | GO:0003230 | cardiac atrium development | 0.000261 | 0.003568 | 0.002608 |
| BP | GO:0061005 | cell differentiation involved in kidney development | 0.000264 | 0.003599 | 0.002631 |
| BP | GO:0006936 | muscle contraction | 0.000266 | 0.003626 | 0.002651 |
| BP | GO:0060537 | muscle tissue development | 0.00027 | 0.003674 | 0.002686 |
| BP | GO:2001057 | reactive nitrogen species metabolic process | 0.000271 | 0.003679 | 0.00269 |
| BP | GO:0006809 | nitric oxide biosynthetic process | 0.000275 | 0.003724 | 0.002723 |
| BP | GO:0014065 | phosphatidylinositol 3-kinase signaling | 0.000276 | 0.003724 | 0.002723 |
| BP | GO:0034138 | toll-like receptor 3 signaling pathway | 0.000278 | 0.003737 | 0.002732 |
| BP | GO:0051960 | regulation of nervous system development | 0.000278 | 0.003737 | 0.002732 |
| BP | GO:0055074 | calcium ion homeostasis | 0.000279 | 0.003739 | 0.002734 |
| BP | GO:0003382 | epithelial cell morphogenesis | 0.000286 | 0.003821 | 0.002794 |
| BP | GO:0110150 | negative regulation of biomineralization | 0.000286 | 0.003821 | 0.002794 |
| BP | GO:0032943 | mononuclear cell proliferation | 0.000295 | 0.003928 | 0.002872 |
| BP | GO:0030705 | cytoskeleton-dependent intracellular transport | 0.000297 | 0.003949 | 0.002887 |
| BP | GO:0060047 | heart contraction | 0.000298 | 0.003956 | 0.002893 |
| BP | GO:0071622 | regulation of granulocyte chemotaxis | 0.000299 | 0.003962 | 0.002897 |
| BP | GO:0033273 | response to vitamin | 0.000308 | 0.004052 | 0.002962 |
| BP | GO:0046849 | bone remodeling | 0.000308 | 0.004052 | 0.002962 |
| BP | GO:0097306 | cellular response to alcohol | 0.000308 | 0.004052 | 0.002962 |
| BP | GO:1903131 | mononuclear cell differentiation | 0.000318 | 0.004172 | 0.003051 |
| BP | GO:0014031 | mesenchymal cell development | 0.00032 | 0.004196 | 0.003068 |
| BP | GO:0019058 | viral life cycle | 0.000323 | 0.004215 | 0.003081 |
| BP | GO:0031397 | negative regulation of protein ubiquitination | 0.000331 | 0.004302 | 0.003146 |
| BP | GO:1990830 | cellular response to leukemia inhibitory factor | 0.000331 | 0.004302 | 0.003146 |
| BP | GO:0048017 | inositol lipid-mediated signaling | 0.000334 | 0.004327 | 0.003164 |
| BP | GO:0045428 | regulation of nitric oxide biosynthetic process | 0.000334 | 0.004327 | 0.003164 |
| BP | GO:0050922 | negative regulation of chemotaxis | 0.000341 | 0.004405 | 0.003221 |
| BP | GO:0071229 | cellular response to acid chemical | 0.000342 | 0.004409 | 0.003223 |
| BP | GO:0031623 | receptor internalization | 0.000344 | 0.004424 | 0.003235 |
| BP | GO:0034599 | cellular response to oxidative stress | 0.000345 | 0.004435 | 0.003243 |
| BP | GO:0010970 | transport along microtubule | 0.00036 | 0.00461 | 0.00337 |
| BP | GO:0072593 | reactive oxygen species metabolic process | 0.000361 | 0.004614 | 0.003373 |
| BP | GO:0048259 | regulation of receptor-mediated endocytosis | 0.000368 | 0.004697 | 0.003434 |
| BP | GO:0003073 | regulation of systemic arterial blood pressure | 0.000369 | 0.004704 | 0.003439 |
| BP | GO:0051251 | positive regulation of lymphocyte activation | 0.000372 | 0.004734 | 0.003461 |
| BP | GO:0008016 | regulation of heart contraction | 0.000381 | 0.004841 | 0.003539 |
| BP | GO:0034109 | homotypic cell-cell adhesion | 0.000386 | 0.004879 | 0.003567 |
| BP | GO:1901201 | regulation of extracellular matrix assembly | 0.000387 | 0.004879 | 0.003567 |
| BP | GO:0032648 | regulation of interferon-beta production | 0.000388 | 0.004879 | 0.003567 |
| BP | GO:0048260 | positive regulation of receptor-mediated endocytosis | 0.000388 | 0.004879 | 0.003567 |
| BP | GO:0034340 | response to type I interferon | 0.000392 | 0.004912 | 0.003591 |
| BP | GO:0045621 | positive regulation of lymphocyte differentiation | 0.000392 | 0.004912 | 0.003591 |
| BP | GO:0002790 | peptide secretion | 0.000393 | 0.004912 | 0.003591 |
| BP | GO:0046651 | lymphocyte proliferation | 0.000396 | 0.004943 | 0.003614 |
| BP | GO:0042476 | odontogenesis | 0.000402 | 0.005005 | 0.003659 |
| BP | GO:0045787 | positive regulation of cell cycle | 0.000404 | 0.005016 | 0.003667 |
| BP | GO:0010743 | regulation of macrophage derived foam cell differentiation | 0.000404 | 0.005016 | 0.003667 |
| BP | GO:1903428 | positive regulation of reactive oxygen species biosynthetic process | 0.000408 | 0.005057 | 0.003697 |
| BP | GO:0002224 | toll-like receptor signaling pathway | 0.000409 | 0.005057 | 0.003697 |
| BP | GO:0001841 | neural tube formation | 0.000417 | 0.00514 | 0.003758 |
| BP | GO:0006801 | superoxide metabolic process | 0.000421 | 0.005179 | 0.003786 |
| BP | GO:0045824 | negative regulation of innate immune response | 0.000424 | 0.005203 | 0.003804 |
| BP | GO:0042063 | gliogenesis | 0.000434 | 0.005325 | 0.003893 |
| BP | GO:0001958 | endochondral ossification | 0.00044 | 0.005378 | 0.003932 |
| BP | GO:0036075 | replacement ossification | 0.00044 | 0.005378 | 0.003932 |
| BP | GO:1903532 | positive regulation of secretion by cell | 0.000447 | 0.005436 | 0.003974 |
| BP | GO:0032816 | positive regulation of natural killer cell activation | 0.000447 | 0.005436 | 0.003974 |
| BP | GO:0031960 | response to corticosteroid | 0.00045 | 0.005459 | 0.003991 |
| BP | GO:0003272 | endocardial cushion formation | 0.000459 | 0.005562 | 0.004067 |
| BP | GO:0048013 | ephrin receptor signaling pathway | 0.000463 | 0.005601 | 0.004095 |
| BP | GO:0044272 | sulfur compound biosynthetic process | 0.000464 | 0.005604 | 0.004097 |
| BP | GO:0035329 | hippo signaling | 0.000466 | 0.005614 | 0.004105 |
| BP | GO:0032944 | regulation of mononuclear cell proliferation | 0.000475 | 0.005704 | 0.00417 |
| BP | GO:1901653 | cellular response to peptide | 0.000485 | 0.005818 | 0.004253 |
| BP | GO:0048846 | axon extension involved in axon guidance | 0.00049 | 0.005853 | 0.004279 |
| BP | GO:1902284 | neuron projection extension involved in neuron projection guidance | 0.00049 | 0.005853 | 0.004279 |
| BP | GO:0032964 | collagen biosynthetic process | 0.000498 | 0.005935 | 0.004339 |
| BP | GO:0001101 | response to acid chemical | 0.000505 | 0.005998 | 0.004385 |
| BP | GO:0120254 | olefinic compound metabolic process | 0.000505 | 0.005998 | 0.004385 |
| BP | GO:0060071 | Wnt signaling pathway, planar cell polarity pathway | 0.000506 | 0.006 | 0.004387 |
| BP | GO:0003222 | ventricular trabecula myocardium morphogenesis | 0.000514 | 0.006063 | 0.004433 |
| BP | GO:0010469 | regulation of signaling receptor activity | 0.000515 | 0.006063 | 0.004433 |
| BP | GO:0061333 | renal tubule morphogenesis | 0.000515 | 0.006063 | 0.004433 |
| BP | GO:0072028 | nephron morphogenesis | 0.000515 | 0.006063 | 0.004433 |
| BP | GO:0030888 | regulation of B cell proliferation | 0.000524 | 0.006146 | 0.004494 |
| BP | GO:0080164 | regulation of nitric oxide metabolic process | 0.000524 | 0.006146 | 0.004494 |
| BP | GO:0034614 | cellular response to reactive oxygen species | 0.000527 | 0.006157 | 0.004502 |
| BP | GO:0051100 | negative regulation of binding | 0.000527 | 0.006157 | 0.004502 |
| BP | GO:0048565 | digestive tract development | 0.000537 | 0.006264 | 0.00458 |
| BP | GO:0014002 | astrocyte development | 0.000556 | 0.006458 | 0.004722 |
| BP | GO:0043114 | regulation of vascular permeability | 0.000556 | 0.006458 | 0.004722 |
| BP | GO:0032814 | regulation of natural killer cell activation | 0.000561 | 0.006473 | 0.004732 |
| BP | GO:0050869 | negative regulation of B cell activation | 0.000561 | 0.006473 | 0.004732 |
| BP | GO:0060323 | head morphogenesis | 0.000561 | 0.006473 | 0.004732 |
| BP | GO:0008217 | regulation of blood pressure | 0.000562 | 0.006473 | 0.004732 |
| BP | GO:0097530 | granulocyte migration | 0.000563 | 0.006473 | 0.004732 |
| BP | GO:0000187 | activation of MAPK activity | 0.000581 | 0.006652 | 0.004863 |
| BP | GO:0001656 | metanephros development | 0.000582 | 0.006652 | 0.004863 |
| BP | GO:0001843 | neural tube closure | 0.000582 | 0.006652 | 0.004863 |
| BP | GO:0002699 | positive regulation of immune effector process | 0.000585 | 0.006682 | 0.004885 |
| BP | GO:0070507 | regulation of microtubule cytoskeleton organization | 0.000594 | 0.006772 | 0.004951 |
| BP | GO:0071692 | protein localization to extracellular region | 0.000617 | 0.00701 | 0.005125 |
| BP | GO:0035907 | dorsal aorta development | 0.000623 | 0.00701 | 0.005125 |
| BP | GO:0045793 | positive regulation of cell size | 0.000623 | 0.00701 | 0.005125 |
| BP | GO:0097084 | vascular associated smooth muscle cell development | 0.000623 | 0.00701 | 0.005125 |
| BP | GO:0003209 | cardiac atrium morphogenesis | 0.000627 | 0.00701 | 0.005125 |
| BP | GO:0007435 | salivary gland morphogenesis | 0.000627 | 0.00701 | 0.005125 |
| BP | GO:0035115 | embryonic forelimb morphogenesis | 0.000627 | 0.00701 | 0.005125 |
| BP | GO:0061311 | cell surface receptor signaling pathway involved in heart development | 0.000627 | 0.00701 | 0.005125 |
| BP | GO:0048708 | astrocyte differentiation | 0.000628 | 0.00701 | 0.005125 |
| BP | GO:0050891 | multicellular organismal water homeostasis | 0.000628 | 0.00701 | 0.005125 |
| BP | GO:0051298 | centrosome duplication | 0.000628 | 0.00701 | 0.005125 |
| BP | GO:0002886 | regulation of myeloid leukocyte mediated immunity | 0.000633 | 0.007034 | 0.005142 |
| BP | GO:0032608 | interferon-beta production | 0.000633 | 0.007034 | 0.005142 |
| BP | GO:0043113 | receptor clustering | 0.000633 | 0.007034 | 0.005142 |
| BP | GO:0033559 | unsaturated fatty acid metabolic process | 0.000635 | 0.007034 | 0.005143 |
| BP | GO:0051924 | regulation of calcium ion transport | 0.000636 | 0.007034 | 0.005143 |
| BP | GO:0002821 | positive regulation of adaptive immune response | 0.000637 | 0.007034 | 0.005143 |
| BP | GO:0030595 | leukocyte chemotaxis | 0.000646 | 0.007072 | 0.00517 |
| BP | GO:0046777 | protein autophosphorylation | 0.000646 | 0.007072 | 0.00517 |
| BP | GO:0001946 | lymphangiogenesis | 0.000648 | 0.007072 | 0.00517 |
| BP | GO:0003184 | pulmonary valve morphogenesis | 0.000648 | 0.007072 | 0.00517 |
| BP | GO:0010544 | negative regulation of platelet activation | 0.000648 | 0.007072 | 0.00517 |
| BP | GO:0034134 | toll-like receptor 2 signaling pathway | 0.000648 | 0.007072 | 0.00517 |
| BP | GO:0042474 | middle ear morphogenesis | 0.000648 | 0.007072 | 0.00517 |
| BP | GO:0070527 | platelet aggregation | 0.00065 | 0.007075 | 0.005173 |
| BP | GO:0010977 | negative regulation of neuron projection development | 0.000651 | 0.007075 | 0.005173 |
| BP | GO:0097242 | amyloid-beta clearance | 0.000657 | 0.007129 | 0.005212 |
| BP | GO:0014909 | smooth muscle cell migration | 0.000659 | 0.007137 | 0.005218 |
| BP | GO:0072507 | divalent inorganic cation homeostasis | 0.000671 | 0.007258 | 0.005306 |
| BP | GO:0030206 | chondroitin sulfate biosynthetic process | 0.000677 | 0.007293 | 0.005332 |
| BP | GO:0072012 | glomerulus vasculature development | 0.000677 | 0.007293 | 0.005332 |
| BP | GO:1903426 | regulation of reactive oxygen species biosynthetic process | 0.000684 | 0.007356 | 0.005378 |
| BP | GO:0019216 | regulation of lipid metabolic process | 0.000685 | 0.007356 | 0.005378 |
| BP | GO:0043651 | linoleic acid metabolic process | 0.000693 | 0.007397 | 0.005408 |
| BP | GO:0048745 | smooth muscle tissue development | 0.000693 | 0.007397 | 0.005408 |
| BP | GO:2000047 | regulation of cell-cell adhesion mediated by cadherin | 0.000693 | 0.007397 | 0.005408 |
| BP | GO:0043506 | regulation of JUN kinase activity | 0.000696 | 0.007407 | 0.005415 |
| BP | GO:0060606 | tube closure | 0.000696 | 0.007407 | 0.005415 |
| BP | GO:0050806 | positive regulation of synaptic transmission | 0.000704 | 0.007483 | 0.005471 |
| BP | GO:0030177 | positive regulation of Wnt signaling pathway | 0.000708 | 0.007513 | 0.005493 |
| BP | GO:0014066 | regulation of phosphatidylinositol 3-kinase signaling | 0.000712 | 0.007539 | 0.005512 |
| BP | GO:0070372 | regulation of ERK1 and ERK2 cascade | 0.000713 | 0.007539 | 0.005512 |
| BP | GO:0050670 | regulation of lymphocyte proliferation | 0.000718 | 0.007581 | 0.005542 |
| BP | GO:0017157 | regulation of exocytosis | 0.000723 | 0.0076 | 0.005556 |
| BP | GO:0070371 | ERK1 and ERK2 cascade | 0.000724 | 0.0076 | 0.005556 |
| BP | GO:0043535 | regulation of blood vessel endothelial cell migration | 0.000726 | 0.0076 | 0.005556 |
| BP | GO:0050654 | chondroitin sulfate proteoglycan metabolic process | 0.000727 | 0.0076 | 0.005556 |
| BP | GO:0051047 | positive regulation of secretion | 0.000729 | 0.0076 | 0.005556 |
| BP | GO:0001570 | vasculogenesis | 0.00073 | 0.0076 | 0.005556 |
| BP | GO:0050886 | endocrine process | 0.00073 | 0.0076 | 0.005556 |
| BP | GO:0010769 | regulation of cell morphogenesis involved in differentiation | 0.000733 | 0.0076 | 0.005556 |
| BP | GO:0061097 | regulation of protein tyrosine kinase activity | 0.000733 | 0.0076 | 0.005556 |
| BP | GO:1904035 | regulation of epithelial cell apoptotic process | 0.000733 | 0.0076 | 0.005556 |
| BP | GO:1903707 | negative regulation of hemopoiesis | 0.000744 | 0.007702 | 0.005631 |
| BP | GO:0030513 | positive regulation of BMP signaling pathway | 0.000765 | 0.007901 | 0.005776 |
| BP | GO:0035137 | hindlimb morphogenesis | 0.000765 | 0.007901 | 0.005776 |
| BP | GO:0023061 | signal release | 0.000773 | 0.007966 | 0.005824 |
| BP | GO:0043112 | receptor metabolic process | 0.000774 | 0.007966 | 0.005824 |
| BP | GO:0071901 | negative regulation of protein serine/threonine kinase activity | 0.0008 | 0.008219 | 0.006009 |
| BP | GO:0048662 | negative regulation of smooth muscle cell proliferation | 0.000802 | 0.008221 | 0.00601 |
| BP | GO:0044706 | multi-multicellular organism process | 0.000804 | 0.008221 | 0.00601 |
| BP | GO:1901136 | carbohydrate derivative catabolic process | 0.000804 | 0.008221 | 0.00601 |
| BP | GO:0002763 | positive regulation of myeloid leukocyte differentiation | 0.000807 | 0.008225 | 0.006013 |
| BP | GO:2000351 | regulation of endothelial cell apoptotic process | 0.000807 | 0.008225 | 0.006013 |
| BP | GO:0120193 | tight junction organization | 0.000829 | 0.00843 | 0.006164 |
| BP | GO:0010959 | regulation of metal ion transport | 0.000839 | 0.008522 | 0.00623 |
| BP | GO:0002706 | regulation of lymphocyte mediated immunity | 0.000841 | 0.008528 | 0.006235 |
| BP | GO:0150117 | positive regulation of cell-substrate junction organization | 0.000875 | 0.008824 | 0.006452 |
| BP | GO:1902751 | positive regulation of cell cycle G2/M phase transition | 0.000875 | 0.008824 | 0.006452 |
| BP | GO:0030104 | water homeostasis | 0.000875 | 0.008824 | 0.006452 |
| BP | GO:0046718 | viral entry into host cell | 0.000881 | 0.008874 | 0.006488 |
| BP | GO:0050808 | synapse organization | 0.000886 | 0.008899 | 0.006506 |
| BP | GO:0009306 | protein secretion | 0.000887 | 0.008899 | 0.006506 |
| BP | GO:0001894 | tissue homeostasis | 0.000893 | 0.008945 | 0.00654 |
| BP | GO:0003188 | heart valve formation | 0.000913 | 0.009108 | 0.006659 |
| BP | GO:0032488 | Cdc42 protein signal transduction | 0.000913 | 0.009108 | 0.006659 |
| BP | GO:0070672 | response to interleukin-15 | 0.000913 | 0.009108 | 0.006659 |
| BP | GO:0061045 | negative regulation of wound healing | 0.000917 | 0.009131 | 0.006676 |
| BP | GO:0014068 | positive regulation of phosphatidylinositol 3-kinase signaling | 0.000923 | 0.009142 | 0.006684 |
| BP | GO:1903035 | negative regulation of response to wounding | 0.000923 | 0.009142 | 0.006684 |
| BP | GO:0030278 | regulation of ossification | 0.000924 | 0.009142 | 0.006684 |
| BP | GO:0090175 | regulation of establishment of planar polarity | 0.000924 | 0.009142 | 0.006684 |
| BP | GO:0032606 | type I interferon production | 0.000934 | 0.009227 | 0.006746 |
| BP | GO:1902106 | negative regulation of leukocyte differentiation | 0.000936 | 0.009231 | 0.006749 |
| BP | GO:0072088 | nephron epithelium morphogenesis | 0.000954 | 0.009389 | 0.006864 |
| BP | GO:0035592 | establishment of protein localization to extracellular region | 0.000956 | 0.009394 | 0.006868 |
| BP | GO:0043524 | negative regulation of neuron apoptotic process | 0.00097 | 0.009517 | 0.006958 |
| BP | GO:2000810 | regulation of bicellular tight junction assembly | 0.000973 | 0.009529 | 0.006967 |
| BP | GO:0048545 | response to steroid hormone | 0.000975 | 0.009529 | 0.006967 |
| BP | GO:0002761 | regulation of myeloid leukocyte differentiation | 0.000977 | 0.009529 | 0.006967 |
| BP | GO:0003014 | renal system process | 0.000977 | 0.009529 | 0.006967 |
| BP | GO:0072577 | endothelial cell apoptotic process | 0.000982 | 0.009561 | 0.00699 |
| BP | GO:0099111 | microtubule-based transport | 0.000993 | 0.009654 | 0.007058 |
| BP | GO:0048661 | positive regulation of smooth muscle cell proliferation | 0.00101 | 0.009793 | 0.00716 |
| BP | GO:0048872 | homeostasis of number of cells | 0.001011 | 0.009793 | 0.00716 |
| BP | GO:0002281 | macrophage activation involved in immune response | 0.001035 | 0.009977 | 0.007294 |
| BP | GO:0007221 | positive regulation of transcription of Notch receptor target | 0.001035 | 0.009977 | 0.007294 |
| BP | GO:0060973 | cell migration involved in heart development | 0.001035 | 0.009977 | 0.007294 |
| BP | GO:0060343 | trabecula formation | 0.001038 | 0.009992 | 0.007305 |
| BP | GO:0045445 | myoblast differentiation | 0.001044 | 0.010032 | 0.007334 |
| BP | GO:0071375 | cellular response to peptide hormone stimulus | 0.001085 | 0.010408 | 0.007609 |
| BP | GO:1903706 | regulation of hemopoiesis | 0.001093 | 0.010473 | 0.007657 |
| BP | GO:0007254 | JNK cascade | 0.001105 | 0.010575 | 0.007732 |
| BP | GO:0070665 | positive regulation of leukocyte proliferation | 0.001161 | 0.011086 | 0.008105 |
| BP | GO:0001938 | positive regulation of endothelial cell proliferation | 0.001163 | 0.011093 | 0.00811 |
| BP | GO:0006874 | cellular calcium ion homeostasis | 0.001167 | 0.0111 | 0.008116 |
| BP | GO:0051962 | positive regulation of nervous system development | 0.001167 | 0.0111 | 0.008116 |
| BP | GO:0002705 | positive regulation of leukocyte mediated immunity | 0.001172 | 0.011127 | 0.008135 |
| BP | GO:0019932 | second-messenger-mediated signaling | 0.001175 | 0.011138 | 0.008143 |
| BP | GO:2001236 | regulation of extrinsic apoptotic signaling pathway | 0.001182 | 0.011187 | 0.008179 |
| BP | GO:0007431 | salivary gland development | 0.001199 | 0.011309 | 0.008268 |
| BP | GO:0034405 | response to fluid shear stress | 0.001199 | 0.011309 | 0.008268 |
| BP | GO:0045601 | regulation of endothelial cell differentiation | 0.001205 | 0.01135 | 0.008298 |
| BP | GO:0002221 | pattern recognition receptor signaling pathway | 0.001211 | 0.01136 | 0.008306 |
| BP | GO:0035265 | organ growth | 0.001212 | 0.01136 | 0.008306 |
| BP | GO:0043583 | ear development | 0.001219 | 0.01136 | 0.008306 |
| BP | GO:0031098 | stress-activated protein kinase signaling cascade | 0.001221 | 0.01136 | 0.008306 |
| BP | GO:0003157 | endocardium development | 0.001221 | 0.01136 | 0.008306 |
| BP | GO:0071492 | cellular response to UV-A | 0.001221 | 0.01136 | 0.008306 |
| BP | GO:0098883 | synapse pruning | 0.001221 | 0.01136 | 0.008306 |
| BP | GO:1902287 | semaphorin-plexin signaling pathway involved in axon guidance | 0.001221 | 0.01136 | 0.008306 |
| BP | GO:0071384 | cellular response to corticosteroid stimulus | 0.001228 | 0.011412 | 0.008343 |
| BP | GO:0018209 | peptidyl-serine modification | 0.001233 | 0.011436 | 0.008361 |
| BP | GO:0014910 | regulation of smooth muscle cell migration | 0.00124 | 0.011483 | 0.008396 |
| BP | GO:0001755 | neural crest cell migration | 0.001244 | 0.011489 | 0.0084 |
| BP | GO:0031529 | ruffle organization | 0.001244 | 0.011489 | 0.0084 |
| BP | GO:0032886 | regulation of microtubule-based process | 0.001292 | 0.011915 | 0.008711 |
| BP | GO:0001990 | regulation of systemic arterial blood pressure by hormone | 0.001363 | 0.01251 | 0.009146 |
| BP | GO:0042092 | type 2 immune response | 0.001363 | 0.01251 | 0.009146 |
| BP | GO:0010758 | regulation of macrophage chemotaxis | 0.001367 | 0.01251 | 0.009146 |
| BP | GO:0010971 | positive regulation of G2/M transition of mitotic cell cycle | 0.001367 | 0.01251 | 0.009146 |
| BP | GO:0034110 | regulation of homotypic cell-cell adhesion | 0.001367 | 0.01251 | 0.009146 |
| BP | GO:0006029 | proteoglycan metabolic process | 0.001369 | 0.01251 | 0.009146 |
| BP | GO:0034332 | adherens junction organization | 0.001448 | 0.013199 | 0.00965 |
| BP | GO:0045600 | positive regulation of fat cell differentiation | 0.001448 | 0.013199 | 0.00965 |
| BP | GO:0045834 | positive regulation of lipid metabolic process | 0.001473 | 0.013397 | 0.009795 |
| BP | GO:0010001 | glial cell differentiation | 0.001474 | 0.013397 | 0.009795 |
| BP | GO:0007204 | positive regulation of cytosolic calcium ion concentration | 0.001493 | 0.013525 | 0.009888 |
| BP | GO:0018105 | peptidyl-serine phosphorylation | 0.001493 | 0.013525 | 0.009888 |
| BP | GO:0061512 | protein localization to cilium | 0.0015 | 0.013547 | 0.009904 |
| BP | GO:0006575 | cellular modified amino acid metabolic process | 0.001501 | 0.013547 | 0.009904 |
| BP | GO:0070997 | neuron death | 0.001502 | 0.013547 | 0.009904 |
| BP | GO:0031099 | regeneration | 0.00151 | 0.013584 | 0.009932 |
| BP | GO:0023035 | CD40 signaling pathway | 0.001522 | 0.013584 | 0.009932 |
| BP | GO:0030852 | regulation of granulocyte differentiation | 0.001522 | 0.013584 | 0.009932 |
| BP | GO:0038065 | collagen-activated signaling pathway | 0.001522 | 0.013584 | 0.009932 |
| BP | GO:0044406 | adhesion of symbiont to host | 0.001522 | 0.013584 | 0.009932 |
| BP | GO:0070831 | basement membrane assembly | 0.001522 | 0.013584 | 0.009932 |
| BP | GO:2000052 | positive regulation of non-canonical Wnt signaling pathway | 0.001522 | 0.013584 | 0.009932 |
| BP | GO:0048483 | autonomic nervous system development | 0.001528 | 0.013622 | 0.009959 |
| BP | GO:0010883 | regulation of lipid storage | 0.001535 | 0.013643 | 0.009975 |
| BP | GO:0044273 | sulfur compound catabolic process | 0.001535 | 0.013643 | 0.009975 |
| CC | GO:0062023 | collagen-containing extracellular matrix | 2.96E-39 | 2.07E-36 | 1.72E-36 |
| CC | GO:0005788 | endoplasmic reticulum lumen | 1.41E-20 | 4.93E-18 | 4.11E-18 |
| CC | GO:0005925 | focal adhesion | 2.60E-17 | 6.05E-15 | 5.04E-15 |
| CC | GO:0030055 | cell-substrate junction | 1.03E-16 | 1.80E-14 | 1.50E-14 |
| CC | GO:0005581 | collagen trimer | 1.15E-15 | 1.61E-13 | 1.34E-13 |
| CC | GO:0005911 | cell-cell junction | 5.34E-14 | 6.22E-12 | 5.18E-12 |
| CC | GO:0005604 | basement membrane | 4.97E-12 | 4.96E-10 | 4.13E-10 |
| CC | GO:0031252 | cell leading edge | 1.32E-11 | 1.16E-09 | 9.62E-10 |
| CC | GO:0045121 | membrane raft | 8.30E-10 | 5.80E-08 | 4.83E-08 |
| CC | GO:0098857 | membrane microdomain | 8.30E-10 | 5.80E-08 | 4.83E-08 |
| CC | GO:0005912 | adherens junction | 5.57E-09 | 3.54E-07 | 2.95E-07 |
| CC | GO:0001726 | ruffle | 8.22E-08 | 4.79E-06 | 3.99E-06 |
| CC | GO:0043202 | lysosomal lumen | 2.01E-07 | 1.01E-05 | 8.42E-06 |
| CC | GO:0098644 | complex of collagen trimers | 2.03E-07 | 1.01E-05 | 8.42E-06 |
| CC | GO:0030027 | lamellipodium | 2.23E-07 | 1.04E-05 | 8.66E-06 |
| CC | GO:0005775 | vacuolar lumen | 3.97E-07 | 1.73E-05 | 1.44E-05 |
| CC | GO:0005884 | actin filament | 1.28E-06 | 5.27E-05 | 4.39E-05 |
| CC | GO:0098636 | protein complex involved in cell adhesion | 3.28E-06 | 0.000128 | 0.000106 |
| CC | GO:0044853 | plasma membrane raft | 7.09E-06 | 0.000261 | 0.000217 |
| CC | GO:0030139 | endocytic vesicle | 9.52E-06 | 0.000333 | 0.000277 |
| CC | GO:0060205 | cytoplasmic vesicle lumen | 1.09E-05 | 0.000362 | 0.000301 |
| CC | GO:0042383 | sarcolemma | 1.14E-05 | 0.000362 | 0.000301 |
| CC | GO:0034774 | secretory granule lumen | 1.35E-05 | 0.000393 | 0.000328 |
| CC | GO:0031983 | vesicle lumen | 1.35E-05 | 0.000393 | 0.000328 |
| CC | GO:0030667 | secretory granule membrane | 1.48E-05 | 0.000413 | 0.000344 |
| CC | GO:0005583 | fibrillar collagen trimer | 2.36E-05 | 0.00061 | 0.000508 |
| CC | GO:0098643 | banded collagen fibril | 2.36E-05 | 0.00061 | 0.000508 |
| CC | GO:0031594 | neuromuscular junction | 3.43E-05 | 0.000856 | 0.000713 |
| CC | GO:0042641 | actomyosin | 3.62E-05 | 0.000873 | 0.000727 |
| CC | GO:0045335 | phagocytic vesicle | 4.02E-05 | 0.000931 | 0.000775 |
| CC | GO:0019897 | extrinsic component of plasma membrane | 4.13E-05 | 0.000931 | 0.000775 |
| CC | GO:0031234 | extrinsic component of cytoplasmic side of plasma membrane | 4.63E-05 | 0.001001 | 0.000833 |
| CC | GO:0032587 | ruffle membrane | 4.72E-05 | 0.001001 | 0.000833 |
| CC | GO:0042470 | melanosome | 5.45E-05 | 0.001088 | 0.000906 |
| CC | GO:0048770 | pigment granule | 5.45E-05 | 0.001088 | 0.000906 |
| CC | GO:0008305 | integrin complex | 5.82E-05 | 0.001109 | 0.000924 |
| CC | GO:0031091 | platelet alpha granule | 5.87E-05 | 0.001109 | 0.000924 |
| CC | GO:0031256 | leading edge membrane | 7.28E-05 | 0.00134 | 0.001116 |
| CC | GO:0030666 | endocytic vesicle membrane | 8.67E-05 | 0.001554 | 0.001294 |
| CC | GO:0001725 | stress fiber | 0.000105 | 0.001796 | 0.001495 |
| CC | GO:0097517 | contractile actin filament bundle | 0.000105 | 0.001796 | 0.001495 |
| CC | GO:0031253 | cell projection membrane | 0.000111 | 0.00185 | 0.001541 |
| CC | GO:0002116 | semaphorin receptor complex | 0.000123 | 0.001996 | 0.001663 |
| CC | GO:0045177 | apical part of cell | 0.000133 | 0.002117 | 0.001763 |
| CC | GO:0005901 | caveola | 0.000138 | 0.002149 | 0.001789 |
| CC | GO:0030427 | site of polarized growth | 0.000147 | 0.002236 | 0.001862 |
| CC | GO:0009898 | cytoplasmic side of plasma membrane | 0.00015 | 0.002236 | 0.001862 |
| CC | GO:0031093 | platelet alpha granule lumen | 0.000212 | 0.00307 | 0.002557 |
| CC | GO:0070820 | tertiary granule | 0.000215 | 0.00307 | 0.002557 |
| CC | GO:0001527 | microfibril | 0.000262 | 0.003607 | 0.003004 |
| CC | GO:0032432 | actin filament bundle | 0.000263 | 0.003607 | 0.003004 |
| CC | GO:0001533 | cornified envelope | 0.000344 | 0.004531 | 0.003773 |
| CC | GO:0098562 | cytoplasmic side of membrane | 0.000345 | 0.004531 | 0.003773 |
| CC | GO:0034399 | nuclear periphery | 0.00035 | 0.004531 | 0.003773 |
| CC | GO:0045178 | basal part of cell | 0.000384 | 0.004884 | 0.004068 |
| CC | GO:0048786 | presynaptic active zone | 0.000403 | 0.005032 | 0.004191 |
| CC | GO:0002102 | podosome | 0.000428 | 0.005248 | 0.004371 |
| CC | GO:0005796 | Golgi lumen | 0.00044 | 0.005302 | 0.004415 |
| CC | GO:0031225 | anchored component of membrane | 0.00046 | 0.00545 | 0.004539 |
| CC | GO:0030670 | phagocytic vesicle membrane | 0.000581 | 0.006678 | 0.005561 |
| CC | GO:0097542 | ciliary tip | 0.000583 | 0.006678 | 0.005561 |
| CC | GO:0030426 | growth cone | 0.000593 | 0.006686 | 0.005568 |
| CC | GO:0005766 | primary lysosome | 0.000621 | 0.006782 | 0.005648 |
| CC | GO:0042582 | azurophil granule | 0.000621 | 0.006782 | 0.005648 |
| CC | GO:0070821 | tertiary granule membrane | 0.00073 | 0.007848 | 0.006535 |
| CC | GO:0042581 | specific granule | 0.001133 | 0.011999 | 0.009993 |
| MF | GO:0005201 | extracellular matrix structural constituent | 7.93E-28 | 8.96E-25 | 8.00E-25 |
| MF | GO:0005178 | integrin binding | 3.54E-12 | 1.51E-09 | 1.35E-09 |
| MF | GO:0005518 | collagen binding | 4.01E-12 | 1.51E-09 | 1.35E-09 |
| MF | GO:0030020 | extracellular matrix structural constituent conferring tensile strength | 1.94E-11 | 5.49E-09 | 4.90E-09 |
| MF | GO:0019838 | growth factor binding | 1.68E-09 | 3.79E-07 | 3.38E-07 |
| MF | GO:0005539 | glycosaminoglycan binding | 2.05E-09 | 3.86E-07 | 3.44E-07 |
| MF | GO:0061134 | peptidase regulator activity | 3.02E-09 | 4.87E-07 | 4.35E-07 |
| MF | GO:0003779 | actin binding | 4.59E-09 | 6.48E-07 | 5.79E-07 |
| MF | GO:0015026 | coreceptor activity | 5.72E-08 | 7.18E-06 | 6.40E-06 |
| MF | GO:0008201 | heparin binding | 2.83E-07 | 3.08E-05 | 2.75E-05 |
| MF | GO:0004867 | serine-type endopeptidase inhibitor activity | 3.00E-07 | 3.08E-05 | 2.75E-05 |
| MF | GO:0061135 | endopeptidase regulator activity | 4.82E-07 | 4.26E-05 | 3.80E-05 |
| MF | GO:0030414 | peptidase inhibitor activity | 4.90E-07 | 4.26E-05 | 3.80E-05 |
| MF | GO:0001968 | fibronectin binding | 5.99E-07 | 4.83E-05 | 4.31E-05 |
| MF | GO:0097493 | structural molecule activity conferring elasticity | 6.46E-07 | 4.87E-05 | 4.34E-05 |
| MF | GO:0004866 | endopeptidase inhibitor activity | 8.65E-07 | 6.11E-05 | 5.45E-05 |
| MF | GO:0001784 | phosphotyrosine residue binding | 1.42E-06 | 9.43E-05 | 8.42E-05 |
| MF | GO:0002020 | protease binding | 2.74E-06 | 0.000172 | 0.000153 |
| MF | GO:0004713 | protein tyrosine kinase activity | 6.02E-06 | 0.000358 | 0.000319 |
| MF | GO:0019955 | cytokine binding | 7.27E-06 | 0.000411 | 0.000367 |
| MF | GO:0004222 | metalloendopeptidase activity | 8.13E-06 | 0.000438 | 0.00039 |
| MF | GO:1990782 | protein tyrosine kinase binding | 9.24E-06 | 0.000475 | 0.000424 |
| MF | GO:0017124 | SH3 domain binding | 1.07E-05 | 0.000524 | 0.000468 |
| MF | GO:0048407 | platelet-derived growth factor binding | 1.27E-05 | 0.000597 | 0.000532 |
| MF | GO:0050840 | extracellular matrix binding | 1.75E-05 | 0.00079 | 0.000705 |
| MF | GO:0017154 | semaphorin receptor activity | 3.35E-05 | 0.001424 | 0.001271 |
| MF | GO:0019199 | transmembrane receptor protein kinase activity | 3.40E-05 | 0.001424 | 0.001271 |
| MF | GO:0004857 | enzyme inhibitor activity | 5.10E-05 | 0.002058 | 0.001837 |
| MF | GO:0004435 | phosphatidylinositol phospholipase C activity | 5.69E-05 | 0.002215 | 0.001977 |
| MF | GO:0042169 | SH2 domain binding | 6.50E-05 | 0.00245 | 0.002186 |
| MF | GO:0045309 | protein phosphorylated amino acid binding | 8.46E-05 | 0.003036 | 0.002709 |
| MF | GO:0008081 | phosphoric diester hydrolase activity | 8.60E-05 | 0.003036 | 0.002709 |
| MF | GO:0004714 | transmembrane receptor protein tyrosine kinase activity | 9.59E-05 | 0.003285 | 0.002932 |
| MF | GO:0051015 | actin filament binding | 0.000124 | 0.004108 | 0.003666 |
| MF | GO:0004629 | phospholipase C activity | 0.000138 | 0.004448 | 0.003969 |
| MF | GO:0051219 | phosphoprotein binding | 0.000156 | 0.004895 | 0.004368 |
| MF | GO:0008237 | metallopeptidase activity | 0.000202 | 0.006172 | 0.005508 |
| MF | GO:0004715 | non-membrane spanning protein tyrosine kinase activity | 0.000218 | 0.006469 | 0.005773 |
| MF | GO:1901681 | sulfur compound binding | 0.00025 | 0.007249 | 0.006469 |
| MF | GO:0017147 | Wnt-protein binding | 0.000301 | 0.008517 | 0.007601 |
| MF | GO:0043178 | alcohol binding | 0.00034 | 0.00936 | 0.008353 |
