# Supplementary material for: Cross-talk of m6A methylation modification and the tumor microenvironment composition in esophageal cancer
Source: Front Immunol. 2025 Jul 7;16:1572810. doi: 10.3389/fimmu.2025.1572810 (PMC12277809; doi:10.3389/fimmu.2025.1572810)
Supplement: Supplementary file 11 [file Table4.docx]

Supplementary Table S4**.** KEGG analysis of differentially expressed genes between two m^6^A modified subtypes.

| ID | Description | pvalue | p.adjust | qvalue |
| --- | --- | --- | --- | --- |
| hsa04510 | Focal adhesion | 1.69E-14 | 5.60E-12 | 4.20E-12 |
| hsa04933 | AGE-RAGE signaling pathway in diabetic complications | 3.32E-11 | 5.49E-09 | 4.12E-09 |
| hsa05205 | Proteoglycans in cancer | 1.23E-09 | 1.36E-07 | 1.02E-07 |
| hsa04512 | ECM-receptor interaction | 8.24E-09 | 6.82E-07 | 5.12E-07 |
| hsa04151 | PI3K-Akt signaling pathway | 1.36E-08 | 9.03E-07 | 6.78E-07 |
| hsa05133 | Pertussis | 1.17E-07 | 6.47E-06 | 4.86E-06 |
| hsa04670 | Leukocyte transendothelial migration | 1.53E-07 | 6.84E-06 | 5.13E-06 |
| hsa04360 | Axon guidance | 1.65E-07 | 6.84E-06 | 5.13E-06 |
| hsa05146 | Amoebiasis | 6.08E-07 | 2.24E-05 | 1.68E-05 |
| hsa05165 | Human papillomavirus infection | 7.68E-07 | 2.54E-05 | 1.91E-05 |
| hsa04926 | Relaxin signaling pathway | 5.20E-06 | 0.000154 | 0.000116 |
| hsa04270 | Vascular smooth muscle contraction | 5.59E-06 | 0.000154 | 0.000116 |
| hsa04514 | Cell adhesion molecules | 6.59E-06 | 0.000168 | 0.000126 |
| hsa05135 | Yersinia infection | 1.01E-05 | 0.000239 | 0.000179 |
| hsa05144 | Malaria | 1.19E-05 | 0.000264 | 0.000198 |
| hsa05418 | Fluid shear stress and atherosclerosis | 1.48E-05 | 0.000306 | 0.00023 |
| hsa05417 | Lipid and atherosclerosis | 1.95E-05 | 0.00038 | 0.000285 |
| hsa05140 | Leishmaniasis | 2.32E-05 | 0.000426 | 0.00032 |
| hsa05167 | Kaposi sarcoma-associated herpesvirus infection | 3.74E-05 | 0.000652 | 0.000489 |
| hsa04974 | Protein digestion and absorption | 5.20E-05 | 0.000861 | 0.000646 |
| hsa04810 | Regulation of actin cytoskeleton | 6.03E-05 | 0.00095 | 0.000713 |
| hsa04014 | Ras signaling pathway | 7.14E-05 | 0.001074 | 0.000806 |
| hsa04010 | MAPK signaling pathway | 0.000133 | 0.001861 | 0.001397 |
| hsa04145 | Phagosome | 0.000135 | 0.001861 | 0.001397 |
| hsa04350 | TGF-beta signaling pathway | 0.000144 | 0.001908 | 0.001432 |
| hsa04015 | Rap1 signaling pathway | 0.000164 | 0.002082 | 0.001562 |
| hsa04062 | Chemokine signaling pathway | 0.000239 | 0.002936 | 0.002204 |
| hsa05142 | Chagas disease | 0.00027 | 0.003192 | 0.002396 |
| hsa04910 | Insulin signaling pathway | 0.000296 | 0.003376 | 0.002534 |
| hsa04380 | Osteoclast differentiation | 0.000347 | 0.003828 | 0.002873 |
| hsa04611 | Platelet activation | 0.000406 | 0.004335 | 0.003253 |
| hsa05163 | Human cytomegalovirus infection | 0.000516 | 0.00534 | 0.004007 |
| hsa05145 | Toxoplasmosis | 0.000651 | 0.006525 | 0.004897 |
| hsa04935 | Growth hormone synthesis, secretion and action | 0.000887 | 0.008637 | 0.006482 |
| hsa04610 | Complement and coagulation cascades | 0.000985 | 0.00932 | 0.006995 |
| hsa05222 | Small cell lung cancer | 0.001426 | 0.013107 | 0.009837 |
| hsa01521 | EGFR tyrosine kinase inhibitor resistance | 0.001914 | 0.017125 | 0.012852 |
| hsa04022 | cGMP-PKG signaling pathway | 0.002018 | 0.017575 | 0.013191 |
| hsa05166 | Human T-cell leukemia virus 1 infection | 0.002109 | 0.0179 | 0.013435 |
| hsa04917 | Prolactin signaling pathway | 0.002164 | 0.01791 | 0.013441 |
| hsa04012 | ErbB signaling pathway | 0.002307 | 0.018304 | 0.013738 |
| hsa04650 | Natural killer cell mediated cytotoxicity | 0.002323 | 0.018304 | 0.013738 |
| hsa05169 | Epstein-Barr virus infection | 0.002601 | 0.019877 | 0.014918 |
| hsa04142 | Lysosome | 0.002642 | 0.019877 | 0.014918 |
| hsa04668 | TNF signaling pathway | 0.002967 | 0.021826 | 0.016381 |
| hsa04666 | Fc gamma R-mediated phagocytosis | 0.00314 | 0.021887 | 0.016427 |
| hsa05100 | Bacterial invasion of epithelial cells | 0.003166 | 0.021887 | 0.016427 |
| hsa04658 | Th1 and Th2 cell differentiation | 0.003174 | 0.021887 | 0.016427 |
| hsa00604 | Glycosphingolipid biosynthesis - ganglio series | 0.003295 | 0.022261 | 0.016707 |
| hsa04664 | Fc epsilon RI signaling pathway | 0.003639 | 0.024092 | 0.018082 |
| hsa05161 | Hepatitis B | 0.003875 | 0.025147 | 0.018873 |
| hsa05170 | Human immunodeficiency virus 1 infection | 0.004017 | 0.025185 | 0.018902 |
| hsa04620 | Toll-like receptor signaling pathway | 0.004107 | 0.025185 | 0.018902 |
| hsa04370 | VEGF signaling pathway | 0.004109 | 0.025185 | 0.018902 |
| hsa04936 | Alcoholic liver disease | 0.004657 | 0.028029 | 0.021036 |
| hsa05410 | Hypertrophic cardiomyopathy | 0.00504 | 0.029791 | 0.022359 |
| hsa05120 | Epithelial cell signaling in Helicobacter pylori infection | 0.005154 | 0.029928 | 0.022461 |
| hsa04931 | Insulin resistance | 0.006983 | 0.039853 | 0.029911 |
| hsa04722 | Neurotrophin signaling pathway | 0.007303 | 0.040971 | 0.030749 |
| hsa04066 | HIF-1 signaling pathway | 0.007917 | 0.043676 | 0.032779 |
| hsa04064 | NF-kappa B signaling pathway | 0.008222 | 0.044613 | 0.033483 |
| hsa04072 | Phospholipase D signaling pathway | 0.008954 | 0.047804 | 0.035877 |
| hsa02010 | ABC transporters | 0.011138 | 0.0576 | 0.04323 |
| hsa04310 | Wnt signaling pathway | 0.011249 | 0.0576 | 0.04323 |
| hsa05414 | Dilated cardiomyopathy | 0.011413 | 0.0576 | 0.04323 |
| hsa04979 | Cholesterol metabolism | 0.011485 | 0.0576 | 0.04323 |
| hsa05215 | Prostate cancer | 0.012929 | 0.063092 | 0.047351 |
| hsa05220 | Chronic myeloid leukemia | 0.013015 | 0.063092 | 0.047351 |
| hsa04520 | Adherens junction | 0.013329 | 0.063092 | 0.047351 |
| hsa04659 | Th17 cell differentiation | 0.013343 | 0.063092 | 0.047351 |
