# Supplementary material for: Cross-talk of m6A methylation modification and the tumor microenvironment composition in esophageal cancer
Source: Front Immunol. 2025 Jul 7;16:1572810. doi: 10.3389/fimmu.2025.1572810 (PMC12277809; doi:10.3389/fimmu.2025.1572810)
Supplement: Supplementary file 12 [file Table5.docx]

Supplementary Table S5**.** 80 m6A-related signature genes

| Gene symbol | HR | Lower limit of confidence interval | Upper limit of confidence interval | pvalue |
| --- | --- | --- | --- | --- |
| RGS16 | 1.390643 | 1.113461 | 1.736825 | 0.003643 |
| SLC2A3 | 1.243566 | 1.029009 | 1.50286 | 0.024077 |
| IFI30 | 1.41161 | 1.055935 | 1.887087 | 0.019943 |
| EDNRA | 1.289607 | 1.014677 | 1.639029 | 0.037611 |
| STC2 | 1.266691 | 1.045908 | 1.534079 | 0.01555 |
| OLFML2B | 1.264873 | 1.029141 | 1.554601 | 0.025553 |
| ADAMTS4 | 1.246611 | 1.021473 | 1.521371 | 0.030081 |
| ENPEP | 1.527884 | 1.135106 | 2.056573 | 0.005177 |
| TSPO | 0.624674 | 0.427922 | 0.911888 | 0.014774 |
| CHST1 | 1.272139 | 1.046343 | 1.546662 | 0.015763 |
| GLA | 1.612564 | 1.187964 | 2.188925 | 0.002179 |
| SNAI1 | 1.365913 | 1.057868 | 1.763659 | 0.016785 |
| GADD45G | 1.302817 | 1.049314 | 1.617564 | 0.016577 |
| ANGPT2 | 1.270123 | 1.015276 | 1.588941 | 0.036381 |
| ULK3 | 0.54206 | 0.325652 | 0.902279 | 0.018498 |
| CD93 | 1.223056 | 1.009787 | 1.481368 | 0.039438 |
| CD14 | 1.265747 | 1.028314 | 1.558003 | 0.02619 |
| RGS4 | 1.293212 | 1.027183 | 1.628139 | 0.028654 |
| MS4A7 | 1.223244 | 1.003406 | 1.491246 | 0.046195 |
| SDS | 1.371861 | 1.070533 | 1.758007 | 0.012469 |
| MED11 | 0.56619 | 0.320572 | 0.999996 | 0.049999 |
| C5AR1 | 1.267676 | 1.013486 | 1.58562 | 0.037775 |
| DYSF | 1.344678 | 1.033971 | 1.748753 | 0.027164 |
| CD300LF | 1.437427 | 1.046958 | 1.973525 | 0.024851 |
| PGF | 1.283109 | 1.0082 | 1.632978 | 0.042729 |
| DKC1 | 1.737665 | 1.149348 | 2.627125 | 0.008793 |
| PCBP3 | 1.683509 | 1.06483 | 2.661646 | 0.025831 |
| BCL6B | 1.347321 | 1.000063 | 1.81516 | 0.049952 |
| IL6 | 1.198508 | 1.027399 | 1.398115 | 0.02123 |
| SEC14L2 | 0.673283 | 0.45828 | 0.989156 | 0.043851 |
| RHPN1 | 1.290985 | 1.014264 | 1.643204 | 0.037983 |
| SOCS3 | 1.342473 | 1.05105 | 1.714698 | 0.018338 |
| SMYD3 | 1.50148 | 1.036104 | 2.175884 | 0.031766 |
| PLCB2 | 1.347938 | 1.003335 | 1.810899 | 0.047472 |
| EPB41L4A | 0.693838 | 0.489253 | 0.983973 | 0.040305 |
| FANCB | 1.610176 | 1.074846 | 2.412129 | 0.020889 |
| DNMT3B | 1.460498 | 1.033307 | 2.064299 | 0.031908 |
| MTHFD2 | 1.400783 | 1.003546 | 1.95526 | 0.047617 |
| ABI3 | 1.459046 | 1.081042 | 1.969224 | 0.013537 |
| CD300A | 1.333662 | 1.001461 | 1.77606 | 0.048844 |
| IRAK1 | 1.524536 | 1.082034 | 2.148001 | 0.015923 |
| ITGB1BP2 | 1.742565 | 1.053798 | 2.881515 | 0.030452 |
| PCDH12 | 1.463947 | 1.052501 | 2.036234 | 0.02358 |
| GPR4 | 1.466009 | 1.066476 | 2.015217 | 0.018452 |
| ABCD1 | 1.344941 | 1.003431 | 1.802681 | 0.047381 |
| STAMBPL1 | 1.33127 | 1.008049 | 1.75813 | 0.043752 |
| ESM1 | 1.26621 | 1.079873 | 1.484701 | 0.00366 |
| FAM50A | 1.618087 | 1.097341 | 2.385954 | 0.01515 |
| SYTL1 | 0.732694 | 0.570971 | 0.940225 | 0.014511 |
| RAD54L | 1.368154 | 1.001721 | 1.868629 | 0.04875 |
| ELK1 | 1.629782 | 1.00435 | 2.644684 | 0.047981 |
| CETP | 1.932936 | 1.149967 | 3.248999 | 0.01287 |
| GNG2 | 1.43695 | 1.026859 | 2.010816 | 0.034467 |
| KCNF1 | 1.431139 | 1.106789 | 1.850543 | 0.006262 |
| IQGAP3 | 1.44416 | 1.087824 | 1.917221 | 0.011014 |
| CILP2 | 0.67192 | 0.468712 | 0.963227 | 0.030475 |
| GPR65 | 1.4254 | 1.034476 | 1.964051 | 0.030219 |
| TTLL12 | 0.715443 | 0.540555 | 0.946914 | 0.019213 |
| PVT1 | 1.575762 | 1.098063 | 2.261277 | 0.013603 |
| SNAP25 | 1.285956 | 1.025927 | 1.611891 | 0.029107 |
| SLC25A14 | 1.743319 | 1.008291 | 3.014173 | 0.046644 |
| SS18L2 | 1.846713 | 1.181829 | 2.885655 | 0.007069 |
| MKNK2 | 0.694296 | 0.495918 | 0.972031 | 0.03357 |
| NCAM2 | 0.395234 | 0.198177 | 0.788237 | 0.0084 |
| PSMB1 | 2.134688 | 1.235979 | 3.686871 | 0.006531 |
| IBSP | 1.410832 | 1.10324 | 1.804184 | 0.006088 |
| MPP1 | 1.466473 | 1.100549 | 1.954064 | 0.008945 |
| SAAL1 | 1.693878 | 1.059524 | 2.708029 | 0.027701 |
| BCR | 0.507109 | 0.313356 | 0.820661 | 0.005698 |
| CRADD | 0.59446 | 0.360101 | 0.981342 | 0.041991 |
| CALCRL | 1.339016 | 1.028938 | 1.742538 | 0.029838 |
| FCN3 | 1.514243 | 1.023659 | 2.239937 | 0.0378 |
| MPI | 0.512908 | 0.281488 | 0.934586 | 0.029187 |
| ZMAT5 | 0.482201 | 0.276224 | 0.841772 | 0.010291 |
| BATF | 1.260831 | 1.020146 | 1.558302 | 0.031991 |
| TM4SF18 | 1.391218 | 1.060814 | 1.824531 | 0.017 |
| PVR | 1.470954 | 1.075916 | 2.011035 | 0.015583 |
| NUP85 | 1.641349 | 1.047043 | 2.572985 | 0.030743 |
| CCDC90B | 0.485795 | 0.258076 | 0.914446 | 0.02528 |
| FLII | 0.606541 | 0.376415 | 0.977356 | 0.03997 |
