# Supplementary material for: Cross-talk of m6A methylation modification and the tumor microenvironment composition in esophageal cancer
Source: Front Immunol. 2025 Jul 7;16:1572810. doi: 10.3389/fimmu.2025.1572810 (PMC12277809; doi:10.3389/fimmu.2025.1572810)
Supplement: Supplementary file 14 [file Table7.docx]

**Supplemental Experimental Procedures**

**Western blot for protein expression**

Following the isolation of tissue proteins, their concentration was ascertained by adhering to the protocol outlined in the BCA assay kit. The extracted proteins were subsequently incorporated into the loading buffer, and subjected to electrophoresis to effectuate the separation of the proteins. Post-electrophoresis, the proteins were transferred onto a PVDF membrane and then incubated with the corresponding antibodies. The subsequent development of the blots was carried out utilizing a Bio-Rad imaging system (USA). The grayscale values of the bands of interest were quantified using Image J software. To serve as an internal control, the levels of GAPDH were ascertained using a mouse anti-human GAPDH antibody (1:5,000 dilutions, Proteintech, Wuhan, China). The relative expression levels of the target proteins were determined by the ratio of the grayscale value of the target protein bands to that of the internal reference bands within the same sample. Each experimental set was conducted in triplicate to ensure reproducibility and statistical significance.

**Clone Formation Assay**

Cells were cultured in 6-well plates, where they adhered to the plate surface and developed visible clones over the course of the culture period. The clone formation rate is indicative of two critical characteristics: cellular dependence on population and their proliferative potential. By monitoring the emergence of single-cell colonies and determining the clone formation rate, we can accurately assess the cells' proliferative capabilities.

**EdU Assay for Cell Proliferation Detection**

Cells were seeded in 6-well plates, and to each well, 10 μmol/L EdU (5-Ethynyl-2'-deoxyuridine) was introduced. The plates were then incubated at 37°C for 2 hours to allow for EdU incorporation. Following this incubation period, the cells were fixed with a 4% formaldehyde solution for 20 minutes at room temperature. After fixation, the cells were washed with phosphate-buffered saline (PBS) to remove any residual formaldehyde. The Click-iT® EdU kit was utilized for the detection of incorporated EdU, with the reaction proceeding for 30 minutes at room temperature. Subsequently, the cells were stained with Hoechst33342 for 20 minutes to visualize the nuclei. The samples were then examined under a fluorescence microscope to assess the staining. The EdU incorporation rate, indicative of cell proliferation, was determined by calculating the ratio of EdU-positive cells (appearing green) to the total number of Hoechst33342-positive cells (appearing blue).

**Wound Healing Assay for Assessing Cellular Migration**

Cells from each experimental group were first trypsinized and then seeded into 6-well plates. They were incubated overnight in medium containing 10% fetal bovine serum (FBS) to allow for adherence and proliferation. Once the cells achieved a confluence of 90% to 100%, a uniform wound was created by gently scraping the cell monolayer at the bottom of the well with a 10 μL pipette tip. The wells were then rinsed three times with phosphate-buffered saline (PBS) to remove any debris and cell fragments. Subsequently, the cells were cultured in serum-free medium within an incubator to facilitate the observation of migration. The progress of cellular migration into the wounded area was monitored using an inverted microscope at 0 and 24 hours post-wounding. Several random fields of view were selected for documentation through photography. The migration distance of the cells was quantified using Image-Pro Plus 7.0 software. Each group contained two replicate wells, and the experiment was conducted in triplicate to ensure reproducibility and statistical validity.

**Transwell migration/invasion assay**

Before the experiment, the polycarbonate membranes of the Transwell inserts were hydrated with serum-free culture medium at 37°C for 30 minutes to ensure optimal conditions for cell attachment. The cells were enzymatically digested, washed with phosphate-buffered saline (PBS), and then resuspended in serum-free medium to achieve the desired cell density, typically around 5 × 10^5^ cells/ml. The cell suspension was carefully added to the upper chambers of the Transwell, while the lower chambers were filled with culture medium supplemented with an attractant factor, such as 20% fetal bovine serum (FBS). The assembled Transwell units were then placed into an incubator for a duration that varied based on the cell type and experimental objectives, generally ranging from 12 to 48 hours. Following the incubation period, the inserts were incubated with PBS at 37°C for 30 minutes to facilitate cell detachment from the upper surface of the membrane. The inserts were then carefully removed, washed with PBS to remove any non-invading or non-migrating cells, and fixed with methanol or 4% paraformaldehyde. Subsequently, the cells that had traversed the membrane were stained with a vital dye such as crystal violet. The invasive or migratory potential of the cells was quantified by microscopically examining and counting the stained cells on the underside of the membrane, providing a direct measure of the cells' ability to penetrate or migrate through the artificial barrier.

**The information of transfection reagents used in this study**

| **Reagent** | Product Number | Target sequence | Company |
| --- | --- | --- | --- |
| Si-RBMX_001 | stB0001866A | AAGTTCTCGTGATACTAGA | Ribobio, Guangzhou, China |
| Si-RBMX_002 | stB0001866B | CTGTATCACGTGGAAGAGA | Ribobio, Guangzhou, China |
| Si-RBMX_003 | stB0001866C | GACTATCCATCAAGAGGAT | Ribobio, Guangzhou, China |

**The information of antibodies used in this study**

| Antibodies | Company | Product Number | Molecular Weight (kDa) |
| --- | --- | --- | --- |
| RBMX | Cell Signaling Technology  (Beverly, MA, USA ) | 60004-1-Ig | 42 |
| GAPDH | Proteintech (Wuhan, China) | 10494-1-AP | 36 |
